# Supplementary material for: Identification of earlier predictors of pregnancy complications through wearable technologies in a Brazilian multicentre cohort: Maternal Actigraphy Exploratory Study I (MAES-I) study protocol
Source: BMJ Open. 2019 Apr 20;9(4):e023101. doi: 10.1136/bmjopen-2018-023101 (PMC6500316; doi:10.1136/bmjopen-2018-023101)
Supplement: Author's manuscript [file bmjopen-2018-023101.draft_revisions.pdf]

BMJ Open is committed to open peer review. As part of this commitment we make the peer review history of every article we publish publicly available.

When an article is published we post the peer reviewers' comments and the authors' responses online. We also post the versions of the paper that were used during peer review. These are the versions that the peer review comments apply to.

The versions of the paper that follow are the versions that were submitted during the peer review process. They are not the versions of record or the final published versions. They should not be cited or distributed as the published version of this manuscript.

BMJ Open is an open access journal and the full, final, typeset and author-corrected version of record of the manuscript is available on our site with no access controls, subscription charges or pay-per-view fees (http://bmjopen.bmj.com).

If you have any questions on BMJ Open's open peer review process please email editorial.bmjopen@bmj.com

BMJ Open

The identification of early predictors of pregnancy complications through wearable technologies in a Brazilian multicenter cohort: Maternal Actigraphy Exploratory Study I (MAES-I)

Journal:	*BMJ Open*
Manuscript ID	bmjopen-2018-023101
Article Type:	Protocol
Date Submitted by the Author:	30-Mar-2018

Complete List of Authors:	Souza, Renato; Universidade Estadual de Campinas, Obstetrics and Gynecology<br>Cecatti, Jose; University of Campinas, Obstetrics and Gynecology<br>Mayrinki, Jussara; Universidade Estadual de Campinas, Obstetrics and Gynecology<br>Galvão, Rafael; Universidade Estadual de Campinas, Obstetrics and Gynecology<br>Costa, Maria Laura; Universidade Estadual de Campinas, Obstetrics and Gynecology<br>Feitosa, Francisco; Universidade Federal do Ceara, Maternidade Escola<br>Rocha Filho, Edilberto; Universidade Federal de Pernambuco, Obstetrics and Gynecology<br>Leite, Debora; Universidade Federal de Pernambuco, Obstetrics and Gynecology<br>Vettorazzi, Janete; Universidade Federal do Rio Grande do Sul, Obstetrics and Gynecology<br>Tedesco, Ricardo; School of Medicine of Jundiai, Obstetrics and Gynecology<br>Santana, Danielly; Universidade Estadual de Campinas, Obstetrics and Gynecology<br>Souza, Joao Paulo; Universidade de Sao Paulo Faculdade de Medicina de Ribeirao Preto, Social Medicine

Keywords:	wearable technologies, actigraph, physical activity, sleep patterns, pregnancy complications

SCHOLARONE™
Manuscripts

STUDY PROTOCOL

The identification of early predictors of pregnancy complications through wearable technologies in a Brazilian multicenter cohort: Maternal Actigraphy Exploratory Study I (MAES-I)

Renato T. Souza [1], Jose G. Cecatti [1#], Jussara Mayrink [1], Rafael B. Galvao [1], Maria L. Costa [1], Francisco E. Feitosa [2], Edilberto A Rocha Filho [3], Débora F Leite [1,3], Janete Vettorazzi [4], Ricardo P Tedesco [5], Danielly S Santana [1,5], Joao P. Souza [6], for the MAES-I Study Group*

Affiliations

[1] Department of Obstetrics and Gynecology, University of Campinas (UNICAMP) School of Medical Sciences, Campinas, SP, Brazil

[2] MEAC – School Maternity of the Federal University of Ceará, in Fortaleza, CE, Brazil

[3] Department of Maternal and Child Health, Maternity of Clinic Hospital, Federal University of Pernambuco, in Recife, PE, Brazil

[4] Department of Obstetrics and Gynecology, Maternity of the Clinic Hospital, Federal University of RS, Porto Alegre, RS, Brazil

[5] Department of Obstetrics and Gynecology, Jundiaí Medical School, Jundiaí, SP, Brazil

[6] Department of Social Medicine, Ribeirão Preto Medical School, University of Sao Paulo, Ribeirão Preto, SP, Brazil

*Membership of the MAES-I study group is provided in the Acknowledgments.

E-mail addresses: RT Souza: renatotsouzasp@gmail.com; JG Cecatti: cecatti@unicamp.br; J Mayrink: jussaramayrink@gmail.com; RB Galvão: rafaelbfg@gmail.com; ML Costa: mlaura@unicamp.br; FE Feitosa: edson.lucena@hotmail.com; EA Rocha Filho: edilbertorocha@globo.com; DF Leite: debora.leite@ufpe.br; J Vettorazzi: janetev@terra.com.br; RP Tedesco: rp.tedesco@yahoo.com.br; DS Santana: dany.fmj@terra.com.br; JP Souza: jp.souza@usp.br

#Corresponding Author

JG Cecatti

DO&G, University of Campinas

R. Alexander Fleming, 101

Campinas, SP, 13083-881

Brazil

E-mail: cecatti@unicamp.br

For peer review only - http://bmjopen.bmj.com/site/about/guidelines.xhtml

Abstract

Introduction: non-invasive tool capable of identifying predictors of maternal complications would be a step forward on the improvement of maternal and perinatal health. There is association between modification of physical activity (PA) and sleep-wake patterns and the occurrence of inflammatory, metabolic, pathologic conditions as chronic diseases. The actigraph device is validated to estimate PA and sleep-wake patterns and might be valuable to identify predictors for maternal complications, widening the window of opportunity to prevent, diagnose or treat specific conditions prior to the development of typical symptoms or clinical signs, assessing PA and sleep-wake patterns during pregnancy.

Methods and analysis: A cohort will be held in 5 centres from the Brazilian Network for Studies on Reproductive and Perinatal Health. MAES-I will enroll 400 low-risk nulliparous women that will wear the actigraph device on the wrist during day and night (24h/day) uninterruptedly from 19-21 weeks until childbirth. Changes in PA and sleep-wake patterns through pregnancy will be analyzed, considering gestational age ranges, in women with and without maternal complications during pregnancy, such as preeclampsia, preterm birth (spontaneous and provider-initiated), gestational diabetes, maternal haemorrhage and also perinatal outcomes. A predictive model for screening pregnant women for risk of specific adverse maternal and perinatal outcomes is planned to be then developed using the actigraphy data.

Ethics and Dissemination: MAES-I study has been reviewed and approved by each Institutional Review Board (IRB) and also by the National Council for Ethics in Research. Detailed information of the study is provided in the Brazilian Cohort website (www.medscinet.com/samba) and findings will be publicized in scientific literature and Institutional webpages.

Discussion: Multiple benefits might arise from the development of a predictive tool for maternal complications during pregnancy, providing a new concept for antenatal care monitoring programmes.

Keywords: wearable technologies; actigraph; physical activity; sleep patterns; sleep-wake cycle; prediction; pregnancy complications.

For peer review only

For peer review only - http://bmjopen.bmj.com/site/about/guidelines.xhtml

Strengths and limitations of this study

- This multicentre cohort will collect comprehensive data on the main maternal and perinatal complications as pre-eclampsia, small for gestational age/fetal growth restriction, preterm birth and gestational diabetes mellitus.
- Physical activity and sleep patterns will be estimated through an inovative wearable device used in the natural environment of the study subject.
- Physical activity and sleep patterns will be estimated from the beginning of the second half of pregnancy until delivery, covering a wide interval during pregnancy and enabling the study of PA and sleep patterns changes throughout pregnancy.
- One possible limitation refers to the uncovered first half of pregnancy regarding this information.

For peer review only

For peer review only - http://bmjopen.bmj.com/site/about/guidelines.xhtml

Background

Reducing the global maternal mortality ratio to less than 70 per 100,000 live births by 2030 is one of the targets of the new United Nations Sustainable Development Goals [1]. Multiple challenges need to be tackled to achieve this target, but the 2016-2030 health and development agenda goes well beyond mortality reduction. The Global Strategy for Women's, Children's and Adolescent's Health aims to ensure that every newborn, women and child not only survive but thrive. This will only be possible if a transformative agenda, with innovation at the central stage, is put into action [2].

One of the major challenges that need to be addressed is optimizing the recognition of early predictors and identifiers of maternal and perinatal complications. Delays in diagnosing and managing maternal complications have been associated with poor outcomes [3]. The reduced self-perception of clinical signs related to maternal complications, difficulties in accessing the health system and poor quality of care may contribute to the late identification of complications and worsened prognosis. The development of a non-invasive Antenatal Care (ANC) tool capable of identifying maternal sub-clinical signs during pregnancy may provide the window of opportunity for early identification of abnormal patterns of physiologic parameters related to pregnancy complications and enable their prevention or early treatment. Shortening the time between the onset of a complication and the initiation of the appropriate management allow for secondary prevention and reduction of maternal morbidity and mortality [3–7].

Pervasive computing (i.e. the trend towards embedding microprocessors in everyday-life objects so they can generate data) and wearable technology (i.e. clothing and accessories incorporating computer and advanced electronic technologies such as wrist and/or waistband sensors) are ubiquitous and able to generate a new dataset that needs to be correlated with pregnancy outcomes. Preterm birth and preeclampsia, for instance, are two important pregnancy complications that have a relatively long subclinical phase before the appearance of signs or symptoms [8,9]. It is plausible that during this subclinical phase of certain conditions the pattern of physical activity (PA) or sleep-wake rhythm is affected in some way and this change could be captured through wearable devices. Although some studies show that PA patterns and

For peer review only - http://bmjopen.bmj.com/site/about/guidelines.xhtml

circadian rhythm abnormalities (actigraphy) may be related to systemic inflammation and diseases in the general population [10,11], published literature correlating wearable technology data and maternal complications are very scarce.

The human circadian rhythm is ruled by endogenous physiologic mechanisms and environmental stimuli [12]. There is solid evidence showing that modification of circadian rhythm or sleep and PA patterns are an underlying condition related to inflammatory, degenerative and/or metabolic chronic diseases as diabetes, hypertension, and cancer [13]. Circadian misalignment is defined as having inappropriate timed sleep and wake, misplaced feeding periods and modification of activity behavior.

The determination of cause or consequence effect between these modifications and the development of pathological conditions is a complex task. It seems that changes in appetite-stimulating hormones, glucose metabolism, inflammatory markers, and mood are some of the related pathways [13–15]. Leproult *et al.* evaluated the effect of circadian misalignment on metabolic and inflammation markers in cardiovascular disease [15]. The insulin action and release, and also the levels of some inflammatory markers that are predictors for cardiovascular diseases, were abnormal in individuals with circadian misalignment. The mechanisms involved in the association between changes of PA pattern and pathologic conditions seem to be of multiple etiologies. Sani *et al.* assessed the circadian rithm of more than 2,300 African descendant adults. More than evaluating physical activity itself, the authors aimed to identify chronobiologic patterns of adults from different socioeconomic settings. The study identified that chronobiologic behavior can vary depending on individual BMI, socioeconomic background, work type and time of sunlight exposure. Possibly, many other factors are involved in modifications of chronobiologic behavior, such as pathologic conditions. Some metabolic, cognitive, cardiovascular and other chronic degenerative diseases have been associated with particular patterns of PA and sleep [10,11,16–18]. A previous observational study assessed various sleep parameters during pregnancy, e.g. sleep onset latency (SOL), wake after sleep onset (WASO) and total nocturnal sleep time (TST). Difficulty in initiating sleep in early pregnancy was associated with higher body mass index, greater weight gain and higher blood pressure during pregnancy

For peer review only - http://bmjopen.bmj.com/site/about/guidelines.xhtml

[17]. Palagini *et al.* reviewed clinical evidence between chronic sleep loss and pregnancy adverse outcomes, discussing common mechanisms of stress system activation [19]. Low-quality evidence suggests an association between sleep loss and prenatal depression, gestational diabetes, preeclampsia, abnormal length of labour, caesarean delivery, abnormal fetal growth, and preterm birth. Those results corroborate with other findings regarding pregnancy and sleep disorders [20–23].

The assessment of PA and the sleep patterns can be performed using small wrist (or waist) devices similar to a regular watch (actigraphy technology). The type of sensor, batteries, materials and output data have been substantially developed in recent years, enabling low cost, comfort, discretion and performance [24]. Nowadays there are devices that are portable, lightweight and with a large capacity to storage information, including a software with automatic scoring algorithms packages for the detection of wakefulness, sleep periods and PA [24,25]. The actigraphy estimation of PA and sleep patterns is validated as a proxy for chronobiologic behavior [26–29] and 7 to 14 days using the actigraph device provides reliable estimates of PA behavior in older adults [30–32]. Both hip and wrist devices show reliable and acceptable performance in estimating PA and sleep-wake patterns [33–36].

The main advantages of using wearable devices for actigraphy is the non-invasiveness, 24/7 monitoring of the circadian pattern and PA, and informing sleep habits and parameters in the user natural environment [24,25,28]. We propose an innovative and strategic approach to monitor PA and sleep-wake patterns during pregnancy, establishing a large database comprised of clinical, epidemiological, PA and sleep-wake variables potentially capable of composing a prediction model for maternal complications during pregnancy. The main goal of this study is to identify early predictors of pregnancy complications by correlating data generated on PA and sleep patterns through wearable devices (wristband sensors) with maternal and perinatal complications and outcomes.

Methods/Design

Study design

For peer review only - http://bmjopen.bmj.com/site/about/guidelines.xhtml

We will conduct a cohort study of 400 low-risk pregnant women using wrist sensor bands able to capture information on daily physical activity and sleep patterns (exposure). This cohort study will be implemented in 5 ANC clinics linked to obstetric units in 3 different regions of Brazil that are already part of the Brazilian Network for Studies on Reproductive and Perinatal Health [37], as shown in Table 1. During a period of eight months, the ANC clinics will identify eligible cases for using the wristband sensors. Wearable technology data will be correlated with the occurrence of pregnancy and childbirth complications and outcomes, thus understood as an effect.

Eligible women will be identified up to 21 weeks of gestation and invited to participate. A proper consent form will be applied and the women who agree to participate will receive a wristband sensor to be used starting at 19-21 weeks until childbirth, uninterruptedly.

Study setting and population

Brazil is a multi-ethnic mixed-race population of diverse resourced settings [38]. Despite the considered high global overall human development index (HDI 0.727) in 2010, the HDI of Brazilian municipalities ranged from 0,862 to 0,418 [39]. The possibility of considering such mixed population is suitable to explore information regarding maternal patterns of mobility and sleep, maximizing external validity and comparisons to other populations. A few reasons might support the study population focused in low-risk nulliparous women: 1) Previous obstetric history can refer to known risk factors for many maternal complications such as preterm birth, preeclampsia, and diabetes [13,40]. Nulliparous women enable unbiased sampling regarding obstetric history. 2) Women with previous morbidity such as hypertension, diabetes, nephropathy or others chronic/degenerative diseases are more likely to present abnormalities of sleep-wake rhythm or physical activity patterns during pregnancy.

Sampling

The five participating centers are regional referral obstetric units responsible for antenatal care assistance mainly for high-risk pregnant women. Participating centers

For peer review only - http://bmjopen.bmj.com/site/about/guidelines.xhtml

are listed in Table 1. Nevertheless, there are primary health care units strategically linked with these participating centers, enabling the identification and enrollment of low-risk pregnant women. The recruitment strategies include approaching all eligible women in these participating centers and their linked facilities. An informed consent form will be applied for women who agree to participate.

Eligible women: Low-risk pregnant subjects

There is no international consensus on the criteria for low-risk pregnancies, although there are several known factors associated with maternal and perinatal adverse outcomes. A recent study evaluating complications of "low-risk" pregnancies of US Americans (10 million births from 2011 through 2013) showed that 29% of low-risk women had an unexpected complication requiring no routine obstetric/neonatal care [41]. This shows the difficulty in establishing a "low-risk profile" for maternal/perinatal complications. As an exploratory study, we will exclude potential known confounders of pre-pregnancy conditions related to adverse maternal or perinatal outcomes as shown in Table 2, in order to assess PA and sleep patterns of a mostly "normal" population. Lifestyle habits and body composition (Body mass index, height, etc.) characteristics, and some non-severe chronic diseases as thyroid disorders, non-severe anaemia and/or asthma are not among exclusion criteria but may be part of subgroup analyses (composition of any previous disorder, e.g.). Intra and inter-individual analyses of PA and sleep patterns enable the identification of potential confounders affecting primary outcomes, avoiding potential biases.

Eligible women will be enrolled between 04-21 weeks. The inclusion and exclusion criteria are presented in Table 2.

Data collection methods

Essentially, MAES I study is comprised of 4 key set points – 3 clinical visits during pregnancy and a postnatal visit. The clinical visits will be held at 1) between 19-21 weeks; 2) between 27-29 weeks; 3) between 37-39 weeks. At first, second, third and postnatal visits, additional information regarding maternal history, details on pregnancy complications, maternal biophysical data (weight, height, skinfolds) and pregnancy adverse outcomes will be collected following a specific Standard Operating

For peer review only - http://bmjopen.bmj.com/site/about/guidelines.xhtml

Procedure (SOP) specially developed for MAES-I study. Additionally, the Perceived Stress Scale [42] and Resilience Scale [43] will be applied during 27-29w visit. Figure 1 shows the set points of MAES-I study.

Eligible women will be invited to use a small wrist device similar to a regular watch (GENEActiv Original – Activinsights®). The device contains sensors that estimate PA and sleep-awake patterns through a proper software algorithm. At the first set point of MAES-I study (between 19+0 - 21+0 weeks of gestation), eligible women who agreed to participate will start using the wrist bracelet device on the non-dominant arm during day and night (24h/day) uninterruptedly until childbirth (including bathing or aquatic activities). The acquisition of actigraphy data can be performed in different frequencies (from 10Hz to 100Hz). Since the frequency of data acquisition impacts on the battery life of the device (inverse relationship), the measurement frequency will be set up according to the participant´s gestational age (Table 3). This information will be registered in the database accordingly. The data accumulated will be downloaded during participant´s antenatal care visits, according to the maximum return periods showed in Table 3. The maximum return periods were calculated taking into consideration the expected battery life.

A leaflet with detailed information and FAQ (Frequently asked questions) on the device will also be provided to the women. They will also have a cell phone number to call whether doubts arise regarding the procedures for using the device, or if any technical or medical concern arises.

All actigraphy data collected will be entered into proper software to interpret data and generate an output file. Then, the actigraphy data will be uploaded to an online database platform developed by MedSciNet®, where all clinical data of the study will also be registered. The actigraphy software uses several algorithms to estimate physical activity and sleep patterns. The database in centralized, secure, internet-based and allows several procedures for prospective and retrospective monitoring, hierarchical access (local user, general manager, etc.). The database will be translated into Portuguese and English, facilitating data collection for Portuguese-speaking team and international monitoring. A correspondent paper form will be available for data collection if necessary (e.g. internet connection failure for instance).

For peer review only - http://bmjopen.bmj.com/site/about/guidelines.xhtml

Decision to start monitoring PA and sleep patterns between 19-21weeks

There are various underlying mechanisms involved in the development of the maternal and perinatal adverse outcomes that will be assessed, as preterm birth, preeclampsia, gestational diabetes, fetal growth restriction and small for gestational age. The pre-clinical phase, stage where there are no clinical signs or symptoms, might be different for each disease and dependent on environmental and individual aspects. The study of adverse maternal and perinatal predictors has been focused in early pregnancy so far (first trimester), aiming to maximize the window of opportunity for preventative interventions. However, we hypothesized that the modification of PA and/or sleep pattern due to maternal underlying changes of biological function might not be evident at a very early stage in pregnancy before the beginning of the pre-clinical phase. Our hypothesis is that it possibly occurs shortly before symptoms.

Additionally, to establish the period between 19-21 weeks as appropriate to start the assessment of PA and sleep patterns taking into consideration that the prevalence of the main maternal complications, as preeclampsia, fetal growth restriction, and preterm birth, are more common in late pregnancy. A recent cross-sectional study conducted in 20 referral centres in Brazil, including the five participating centres of this proposal, showed that the occurrence of preterm birth before 28 weeks comprised less than 1% of all births and less than 8% of all preterm births [44]. In addition, the early onset of preeclampsia (before 34 weeks of gestation) complicates less than 0.4% of all pregnancies, according to a large retrospective cohort of more than 450,000 deliveries in USA [45]. Figure 2 outlines the predicted prevalence of preterm birth and preeclampsia in the second trimester, highlighting its clinical presentation through pregnancy in red. Our hypothesis is that PA and sleep patterns might be altered closely to the clinical presentation, still in preclinical phase. Thus, the start of assessment between 19-21 weeks seems to be very reasonable, providing a wide interval to monitor and predict the main maternal and perinatal adverse outcomes.

Actigraph device

The actigraph device that will be used to monitor PA and sleep-wake patterns is GENEActiv Original (GENEActiv, Activinsights Ltd, Huntingdon, UK). The device has multiple sensors as microelectromechanical (MEMS) accelerometer, temperature

For peer review only - http://bmjopen.bmj.com/site/about/guidelines.xhtml

(linear active thermistor) and light (silicon photodiode), providing crude raw data for a variety of applications.

Wrist vs waist wear: advantages and performance

Wrist wear of actigraph devices provides more comfortable use during wake and sleep periods and highest wear time compared to waist monitors [33,46]. A non-systematic review published in 2011 showed that actigraphy is a useful and reliable tool to assess sleep patterns and circadian rhythm disorders, although there are some limitations on diagnosing sleep diseases or measuring sleep stages [25]. Actigraphy showed excellent concordance with polysomnography in assessing sleep parameters in healthy subjects (sensitivity >90% in estimating total sleep time, for instance). A recent study evaluated the concordance of physical activity estimation of wrist device in free-living settings in forty overweight or obese women [34]. They used both wrist and hip devices, and a small camera that captured participant behaviour for 7 days, enabling the monitoring of physical activity behaviour (gold-standard comparison). The hip and wrist machine learning (ML) classifiers used are different due to the different methods/algorithms to estimate physical activity [34]. The sensitivity and specificity of hip and wrist estimations according to Ellis *et al* are showed in Table 4 [34].

Two years ago, the same author had published a similar evaluation using 40 adults (women and men), showing that the hip and wrist accelerometers obtained an average accuracy of 92.3% and 87.5%, respectively, in predicting PA types [47].

Staudenmayer *et al* developed an investigation with 20 participants also using two devices (wrist- and hip- worn), and showed that wrist actigraphy can estimate energy expenditure accurately and relatively precisely [48]. Another study evaluating PA patterns in a free-living environment with wrist devices showed that women in the top 40% or bottom 40% of the distribution of daily PA, hip and wrist accelerometers agreed on the classification for about 75% of the women [49]. Additionally, the total activity (counts per day) was moderately correlated (Spearman's r = 0.73) between the wrist and hip worn devices.

At the best of our knowledge, there are no systematic reviews or other high-quality evidence-based recommendation supporting a particular method. Although wrist wear of actigraphy is more conventional and accurate, it might not be the best choice for

For peer review only - http://bmjopen.bmj.com/site/about/guidelines.xhtml

assessing long periods of PA or sleep patterns, even more considering the similar performance of the wrist wear. The current proposal does not intend to diagnose pathologic behaviours or diseases, but to identify different patterns along pregnancy and in different subgroups of women. Therefore, supported by the evidence that wrist wear of actigraphy devices can accurately and more comfortably estimate PA and sleep patterns, mainly for long periods and in the free-living environment, the MAES-I study group addopted wrist wear devices.

Main variables

The independent variables assessed as potential predictors of maternal complications will be related to sleep-wake cycle and mobility as:

"Sleep" variables

- Sleep onset latency (SOT): time elapsed between full wakefulness to sleep.
- Total sleep time (TST): The amount of actual sleep time in a sleep episode (excludes awakes).
- Wake after sleep onset (WASO): defined as total amount of time awake after sleep.
- Sleep Efficiency (SE): the proportion of sleep in the period potentially filled by sleep-ratio of total sleep time to time in bed.

The actigraph device collects many pieces of information related to body position and body movements to estimate the described sleep variables. Then, actigraphy software will be used to analyse the data and generates the output variables.

"Physical activity" variables

Actigraphy technology estimates physical activity through various parameters collected by the actigraph device. Briefly, according to Freedson *et al*, the triaxial sensors stressed by acceleration forces can estimate the intensity of movements. The acceleration signal is converted to digital signal and summed over a user specified time interval (epoch). At the end of each epoch the activity count is stored. Then, according to Count per minute (CPM) cut points, the PA intensity can be categorized [50]. The information is translated by the software using proper algorithms into quantitative variables as following:

For peer review only - http://bmjopen.bmj.com/site/about/guidelines.xhtml

- Sedentary time (hours/day): the number of hours per day of count per minute between 0-99.
- Light activity (hours/day): the number of hours per day having count per minute between 100 - 1951.
- Moderate activity (minutes/day): the number of hours per day having count per minute between 1952 - 5724.
- Vigorous activity (minutes/day): the number of hours per day having count per minute between 5725 - 9498.
- Very vigorous activity (minutes/day): the number of hours per day having count per minute between 9499 - ∞.
- MET rates: Metabolic Equivalents (METs) are commonly used to also express the intensity of physical activities. One MET is the energy cost of resting quietly, often defined in terms of oxygen uptake as 3.5 $mL{\cdot}kg^{-1}{\cdot}min^{-}1$. MET rate expresses a person's working metabolic rate relative to their resting metabolic rate. Briefly, the triaxial piezoelectric sensors stressed by acceleration forces can estimate the intensity of movements, converted to the oxygen consumption required to perform such movement.
- Step counts/day: estimated steps count per day.

Outcomes

The primary outcomes are late pregnancy complications as:

- Preeclampsia: Hypertension after 20 weeks of gestation, systolic BP ≥ 140mmHg and/or diastolic BP ≥ 90mmHg (Korotkoff V) on at least 2 occasions 4h apart with: 1) Proteinuria 300 mg/24h or spot urine protein: creatinine ratio 30 mg/mmol creatinine or urine dipstick protein ≥ (+) OR, in the absence of proteinuria, hypertension and 2) any multi-system complication that are: Haematological abnormalities; thrombocytopenia (platelets < 100 x 10^9/L); disseminated intravascular coagulation (DIC), and haemolysis; Liver disease: increased aspartate transaminase and/or alanine transaminase > 45 IU/L and/or severe right upper quadrant or epigastric pain, liver rupture; Neurological problems:

For peer review only - http://bmjopen.bmj.com/site/about/guidelines.xhtml

eclampsia, imminent eclampsia (severe headache with hyperreflexia and persistent visual disturbance), cerebral haemorrhage; Acute renal insufficiency: new increase in serum creatinine to > 100 mmol/L antepartum or > 130 mmol/L postpartum; Pulmonary oedema confirmed by chest x-ray [51].

- Gestational Diabetes: new diabetes developing in pregnancy according to the WHO recommendation [52] that defines gestational diabetes as having:
  - Fasting plasma glucose ≥ 92 mg/dl, or
  - 1-h plasma glucose tolerance test (75g load) ≥ 180 mg/dl, or
  - 2-h plasma glucose tolerance test (75g load) ≥ 153 mg/dl.
- Spontaneous Preterm Birth: spontaneous onset of preterm labour or premature rupture of membranes leading to preterm birth, childbirth before 37 weeks of gestation.
- Provider-Initiated Preterm Birth: defined as childbirth occurring at less than 37 weeks, medically indicated due to maternal/fetal compromise or both;
- Maternal Hemorrhage: Classified in 1) Antepartum haemorrhage defined as bleeding from the genital tract after 24 weeks of gestation; 2) Primary Postpartum haemorrhage as the loss of 500 ml blood or more from the genital tract within 24 hours of the childbirth.

Secondary outcomes include childbirth variables and neonatal adverse outcomes as fetal death, caesarean section, small for gestational age (defined as birth weight below percentile 10 for gestational age), Apgar score < 7 at 5 minutes, neonatal severe morbidity (Table 5) and neonatal mortality before discharge.

Plans for analyses

Sample size estimation

This is an exploratory and innovative study focused on a specific population (pregnant women) and therefore there are no previously published parameters available for sample size estimation. Considering a relatively wide range of frequency of complications arising in pregnancy from 3 to 20% (including preeclampsia, fetal growth restriction, gestational diabetes, hemorrhage, preterm birth, etc.), a theoretical large population size (above 1 million pregnant women), an acceptable margin of error of 4%, the involvement of 5 clusters (participating centers) and a 95% level of confidence,

For peer review only

384 women would be necessary. Therefore, we are rounding up this estimation for at least 400 initially low-risk pregnant women to be enrolled in the study. We estimated the incidence of some main maternal complications considering the following studies:

- Pre-eclampsia: An international prospective cohort study with nulliparous women called SCOPE used similar criteria for the low-risk profile, having 5% of incidence of pre-eclampsia [53].
- Preterm Birth: A recent cross-sectional study conducted in 20 referral obstetric centres in Brazil, including the five participant centres of this proposal, showed a prevalence of 12.3% of all births [44].
- Gestational Diabetes: SCOPE international cohort, previously mentioned, had a prevalence of 8.9% of gestational diabetes in low-risk screened nulliparous women, according to mainly to the NICE guidelines [54].
- Fetal growth restriction/small for gestational age: SCOPE international cohort, previously mentioned, had a prevalence of 10.7% of newborns small for gestational age, according to the customized centiles of birthweight (<10%)[55].

Analyzes and statistics details

According to these studies, the predicted incidence of these complications, the leading causes of maternal and perinatal adverse outcomes, seems adequate for the current proposal and sample size estimation, although the complications are not cumulative.

Firstly, we will identify PA and sleep-wake patterns of women who did not develop adverse maternal or perinatal outcomes. It will permit the recognition of a normal PA and sleep-wake patterns in low-risk population without complication during pregnancy. Using the same population, we will analyze changes in PA and sleep-wake patterns through pregnancy, allowing for gestational age periods.

Then, we will compare the PA and sleep-wake patterns of women who developed specific adverse maternal or perinatal outcomes with those who did not. The differences between groups might be identified to be used as potential markers for specific pregnancy complications.

After that, we will analyze changes in PA and sleep-wake patterns of women who developed adverse maternal or perinatal outcomes through pregnancy, comparing the

For peer review only

patterns before with those after the onset of maternal complications, trying to discover which changes might be related to pregnancy complications.

Finally, we will develop a predictive model for screening pregnant women for risk of specific adverse maternal and perinatal outcomes using PA and sleep-wake data estimated with actigraphy technology.

The analysis will be performed using the actigraph software that translates the collected information into PA and sleep-wake parameters. Additionally, Friedman and Wilcoxon for paired samples, t-test, and ANOVA for repeated measures will be applied to achieve statistical analyses.

Discontinuation of participants

The criteria for discontinuation include:

- Withdrawal of consent;
- Not regularly using the actigraph device for long periods, above 50% of all planned time. The information that they are not using the device properly will be recorded if women notice the MAES-I team. Otherwise, the low use of the device will be noticed after data discharge during antenatal care visits.
- The loss to follow-up, not allowing the download of actigraphy data.

Data and Sample Quality

All entered data will be prospectively and retrospectively monitored by local research assistants and a global monitor. Internal consistency of variables will be constantly performed by the database and error messages are automatically flagged. A local research assistant will be responsible for checking all forms and actigraphy data before locking forms, assuring good quality of data. Then, the local principal investigator (PI) will be responsible for signing the case, enabling its incorporation to the final database. The University of Campinas will coordinate, implement and monitor the study in the five participating centres. A general manager and a global monitor are also part of the team of the coordinating centre. The local team of each participating centre is comprised of a Local PI and research assistants.

Ethical aspects

For peer review only - http://bmjopen.bmj.com/site/about/guidelines.xhtml

MAES-I study focuses on low-risk nulliparous Brazilian pregnant women. Despite being considered of low risk for maternal and perinatal complications, they are not free of suffering complications. Furthermore, the first and second delays, defined as a delay in deciding to seek care and delay in reaching a health care facility [56], are not uncommon, establishing a barrier between early recognition of symptoms and timely interventions capable to successfully treat potentially life-threatening conditions. We believe that women will feel encouraged, empowered and willing to participate in the study that aims to develop a potentially useful prenatal care tool to identify the risk for maternal and perinatal morbidity and mortality. Following national ethical regulations, the participants will not receive any financial compensation.

Women who agree to participate in the study will not have any disadvantages or injury of their prenatal care. On the contrary, they will receive a telephone number to contact the clinical researchers at any time (24/7 service), which enables a closer contact with researchers and providers of care, since the MAES-I team are committed to contacting providers of care if any potential complication is noticed by participants.

The participating women will not be responsible if loss, theft or damage to the wrist device occurs. However, they will be asked to return the device after they finished the participation in the study, in order to use it for new women entering the study. No self-damage is expected in those who use the device. The identity of all women will be kept confidential.

Participating women will not be able to identify any PA or sleep parameters at any stage of the study. The download of the data is only possible through the own licensed software of the device.

MAES-I study has been reviewed and approved by the National Committee for Ethics in Research of Brazil (CONEP) and by the Institutional Review Board (IRB) of the coordinating centre (Letter of approval 1.834.116 issued on 24th November 2016) and of all other Brazilian participating centres. All women who will be enrolled in the MAES-I cohort will sign an informed consent form.

The ethical principles stated in the Brazilian National Heath Council (Resolution CNS 466/12) will be respected in every stage of this study. The anonymity of the source of

For peer review only - http://bmjopen.bmj.com/site/about/guidelines.xhtml

information will be guaranteed and the care for the women will be provided independently of her agreement to participate in the study. The study also complies with the Declaration of Helsinki amended in Hong Kong in 1989.The methodological and ethical aspects of MAES-I study protocol were developed following STROBE guidelines [57].

Patient and Public Involvement

Patients and public were not involved in this study for the development of the research question and outcome measures. However, the choice for a wrist device was based on the preference of users as reported. Participants of the study will have access to the results by its webpage that will open access.

Discussion

The actigraphy is an innovative, non-invasive, non-operator dependent, wearable technology, which enables the estimative under real life conditions of diverse variables related to mobility, physical activity, sleep-wake, and circadian cycle patterns. Actigraph devices show high sensitivity in sleep-wake parameters detection and are currently highly recommended by the American Sleep Disorder Association for diagnosis and therapy response of circadian rhythm disorders [27,28,58]. Although some studies show that 7 to 14 days using the actigraph device provides reliable estimates of physical activity behavior in older adult, it is not absolutely clear how many days is needed to estimate habitual PA by using the wrist/waist device during pregnancy. In general, it seems to depend mainly on the type of actigraph device, position of wear and target population [30,33]. Nevertheless, MAES-I study will provide sufficient data to assess different patterns along pregnancy.

The use of wearable physical activity monitors has grown enormously due to the interest about the relationship between the pathophysiology of diseases and physical activity and sleep patterns. A recent study on the use of physical activity monitors in human physiology research unravels the current and potential uses of actigraph device as in strategies to promote healthier behaviour or to predict outcomes [59]. The authors conclude that physical activity monitors, as others new 21st century

For peer review only - http://bmjopen.bmj.com/site/about/guidelines.xhtml

technologies, have already transformed physiology research, revolutionizing the way we assess patients and opening new areas of interest. In addition, the use of objectives measures to evaluate habitual sleep duration and outcomes in pregnancy is critical, taking into account recent investigations reporting little agreement between objective and subjective assessments of sleep time [60].

Alterations in sleep patterns, as less deep sleep and more nocturnal awakenings, can be observed in pregnancy as early as 10-12 weeks gestation [61]. Sleep disturbances during pregnancy have been associated with preterm delivery, gestational hypertensive disorders, glucose intolerance and increased risk of caesarean delivery [24]. Shorter night time sleep was also associated with hyperglycemia [60]. Persistent sleep deficiency is correlated with depressive symptoms and stress perception by pregnant women [61]. These studies lay correlation between PA patterns and sleep disturbances determining complications, in a well-established relationship of cause and consequence, although sometimes it could not be adequately determined due to the study design [17].

In a distinct way, our analysis intends to figure out if the maternal complication could be identified by physical activity and/or sleep patterns modifications, even during its pre-clinical period, previous the appearance of clinical signs. Considering the existing evidence, we speculate that the PA and/or sleep patterns change days or weeks before the clinical presentation of the complication. In general, the signs and symptoms of some maternal outcomes are part of the gold-standard criteria for diagnosis (high blood pressure, proteinuria and/or edema in the case of preeclampsia; premature contractions and cervical ripening/dilation in preterm birth; abnormal placental blood flow and insufficient fetal growth in Intrauterine Growth Restriction).

Then, the use of actigraph device during prenatal visits has a potential to become a new tool to monitor pregnant women, improving maternal health care, identifying altered PA and/or sleep patterns, measured objectively through actigraphy, before the occurrence of those signs and symptoms. Therefore, the focus would be offering new technology to monitor the development of a potential maternal complication. Other positive points of our study are the period of data collection (from 19 weeks till delivery) and the low-risk profile of the cohort. Through which, it would be possible to

For peer review only - http://bmjopen.bmj.com/site/about/guidelines.xhtml

describe a PA and sleep patterns in a low-risk pregnant population and better interpret actigraphy data among pregnant women. The current clinical and biological predictors for the main maternal complications as preeclampsia, preterm birth, maternal haemorrhage, and gestational diabetes still lack for effective sensitivity and specificity.

If this is confirmed to be true, an important step will be achieved for a possible introduction of screening non-invasive procedures during prenatal care with the purpose of identifying women at higher risk of developing those conditions. Therefore, they could receive specific orientation on prevention and earlier detection of the onset of condition for taking immediate action to look for professional health care and receiving appropriate interventions, avoiding delays that are the most striking factor for the low quality of care the women usually receive in low and middle-income settings, contributing to the still high burden of maternal morbidity and mortality. If we were successful in identifying such "specific patterns of physical activity and sleep" as predictors for pregnancy complications, further validation studies will necessarily be recommended for assessing its effectiveness for the whole management of such conditions. Additionally, MAES-I will enable further specific studies among high risk population and also will help to identify the best gestational age for monitoring, giving the means to target a specific gestational age interval.

For peer review only

Abbreviations

ANC – antenatal care

BMI – body mass index

BP – blood pressure

CPM – count per minute

dL – decilitre

FAQ – frequently asked questions

h – hour

HDI – human development index

Hz - hertz

Kg – kilogram

L – litre

MAES-I – maternal Actigraphy Exploratory Study I

MEMS – microelectromechanical

MET – metabolic equivalents

min – minutes

mg – milligram

mL – millilitre

mmol – millimole

NICE – National Institute for Health and Care Excellence

PA – physical activity

PI – Principal investigator

SE – sleep efficiency

SCOPE – SCreening Of Pregnancy Endponits

SOL – sleep onset latency

TST – total nocturnal sleep time

US – United States

USA – United States of America

w – week

Competing interests

The authors declare that they have no competing interests.

Authors' contributions

All authors contributed to the overall study design and specific methodologies. RTS, JGC, JM, MLC and JPS conceived the study design. RTS, JGC, JM, and RBG planned the implementation of the study. RTS, JM, MLC and JGC drafted the manuscript. All authors discussed and made important contributions to the manuscript, read and approved the final version for submission.

Acknowledgements

The MAES - I study group also included: Carina B Luiz and Luiza C Brust, School of Medicine, Federal University of Rio Grande do Sul, Porto Alegre, Brazil; Danilo Anacleto and Lívia C Nascimento, School of Medicine, Federal University of Pernambuco, Recife, Brazil; Daisy Lucena and Denise Ellen F Cordeiro, School of Medicine, Federal University of Ceará, Fortaleza, Brazil; Mariana B Rogerio, Departament of Obstetrics and Gynecology, Jundiaí Medical School, Jundiaí, Brazil.

For peer review only - http://bmjopen.bmj.com/site/about/guidelines.xhtml

Funding

This study was granted by The Bill and Melinda Gates Foundation through the Grand Challenge Exploration program, call 19 (research grant OPP1182749).

For peer review only

For peer review only - http://bmjopen.bmj.com/site/about/guidelines.xhtml

References

1 United Nations Development Programme (UNDP). Sustain. Dev. Goals. 2015.http://www.un.org/sustainabledevelopment/health/ (accessed 27 Apr 2016).

2 WHO, World Health Organization. Global Strategy. Glob. Strateg. Women's, Child. Adolesc. Heal. 2016 - 2030. 2015.http://www.who.int/life-course/partners/global-strategy/global-strategy-2016-2030/en/ (accessed 9 Jul 2016).

3 Pacagnella RC, Cecatti JG, Parpinelli MA, *et al.* Delays in receiving obstetric care and poor maternal outcomes: results from a national multicentre cross-sectional study. *BMC Pregnancy Childbirth* 2014;**14**:159. doi:10.1186/1471-2393-14-159

4 Haddad SM, Cecatti JG, Souza JP, *et al.* Applying the maternal near miss approach for the evaluation of quality of obstetric care: a worked example from a Multicenter Surveillance Study. *Biomed Res Int* 2014;**2014**:989815. doi:10.1155/2014/989815

5 Pacagnella RC, Cecatti JG, Osis MJ, *et al.* The role of delays in severe maternal morbidity and mortality: expanding the conceptual framework. *Reprod Health Matters* 2012;**20**:155–63. doi:10.1016/S0968-8080(12)39601-8

6 Vogel JP, Souza JP, Mori R, *et al.* Maternal complications and perinatal mortality: findings of the World Health Organization Multicountry Survey on Maternal and Newborn Health. *BJOG* 2014;**121 Suppl**:76–88. doi:10.1111/1471-0528.12633

7 Souza JP, Gülmezoglu AM, Vogel J, *et al.* Moving beyond essential interventions for reduction of maternal mortality (the WHO Multicountry Survey on Maternal and Newborn Health): a cross-sectional study. *Lancet* 2013;**381**:1747–55. doi:10.1016/S0140-6736(13)60686-8

8 Brosens I, Pijnenborg R, Vercruysse L, *et al.* The 'Great Obstetrical Syndromes' are associated with disorders of deep placentation. *Am J Obstet Gynecol* 2011;**204**:193–201. doi:10.1016/j.ajog.2010.08.009

9 Di Renzo GC. The great obstetrical syndromes. *J Matern neonatal Med* 2009;**22**:633–5. doi:10.1080/14767050902866804

10 Loprinzi PD. The effects of objectively-measured, free-living daily ambulatory movement on mortality in a national sample of adults with diabetes. *Physiol Behav* 2016;**154**:126–8. doi:10.1016/j.physbeh.2015.11.022

11 Biswas A, Oh PI, Faulkner GE, *et al.* Sedentary Time and Its Association With Risk for Disease Incidence, Mortality, and Hospitalization in Adults. *Ann Intern Med* 2015;**162**:123. doi:10.7326/M14-1651

12 Hardin PE. From biological clock to biological rhythms. *Genome Biol* 2000;**1**:reviews1023.1-1023.5. doi:10.1186/gb-2000-1-4-reviews1023

13 Kizaki T, Sato S, Shirato K, *et al.* Effect of Circadian Rhythm on Clinical and Pathophysiological Conditions and Inflammation. *Crit Rev Immunol* 2015;**35**:261–75.

14 Baron KG, Reid KJ. Circadian misalignment and health. *Int Rev Psychiatry* 2014;**26**:139–54.

For peer review only - http://bmjopen.bmj.com/site/about/guidelines.xhtml

15 Leproult R, Holmbäck U, Van Cauter E, *et al.* Circadian misalignment augments markers of insulin resistance and inflammation, independently of sleep loss. *Diabetes* 2014;**63**:1860–9. doi:10.2337/db13-1546

16 Diem SJ, Blackwell TL, Stone KL, *et al.* Measures of Sleep-Wake Patterns and Risk of Mild Cognitive Impairment or Dementia in Older Women. *Am J Geriatr Psychiatry* 2016;**24**:248–58. doi:10.1016/j.jagp.2015.12.002

17 Haney A, Buysse DJ, Rosario BL, *et al.* Sleep disturbance and cardiometabolic risk factors in early pregnancy: a preliminary study. *Sleep Med* 2014;**15**:444–50. doi:10.1016/j.sleep.2014.01.003

18 Lynch BM, Boyle T, Winkler E, *et al.* Patterns and correlates of accelerometer-assessed physical activity and sedentary time among colon cancer survivors. *Cancer Causes Control* 2016;**27**:59–68. doi:10.1007/s10552-015-0683-4

19 Palagini L, Gemignani A, Banti S, *et al.* Chronic sleep loss during pregnancy as a determinant of stress: impact on pregnancy outcome. *Sleep Med* 2014;**15**:853–9. doi:10.1016/j.sleep.2014.02.013

20 Tsai S-Y, Lin J-W, Wu W-W, *et al.* Sleep Disturbances and Symptoms of Depression and Daytime Sleepiness in Pregnant Women. *Birth* 2016;**43**:176–83. doi:10.1111/birt.12215

21 Tsai S-Y, Lee P-L, Lin J-W, *et al.* Cross-sectional and Longitudinal Associations between Sleep and Health-related Quality of Life in Pregnant Women: a Prospective Observational Study. *Int J Nurs Stud* 2016;**56**:45–53. doi:10.1016/j.ijnurstu.2016.01.001

22 Hayase M, Shimada M, Seki H. Sleep quality and stress in women with pregnancy-induced hypertension and gestational diabetes mellitus. *Women Birth* 2014;**27**:190–5. doi:10.1016/j.wombi.2014.04.002

23 Mindell JA, Cook RA, Nikolovski J. Sleep patterns and sleep disturbances across pregnancy. *Sleep Med* 2015;**16**:483–8. doi:10.1016/j.sleep.2014.12.006

24 JOHN D, FREEDSON P. ActiGraph and Actical Physical Activity Monitors. *Med Sci Sport Exerc* 2012;**44**:S86–9. doi:10.1249/MSS.0b013e3182399f5e

25 Martin JL, Hakim AD. Wrist actigraphy. *Chest* 2011;**139**:1514–27. doi:10.1378/chest.10-1872

26 Wood AC, Kuntsi J, Asherson P, *et al.* Actigraph data are reliable, with functional reliability increasing with aggregation. *Behav Res Methods* 2008;**40**:873–8.

27 Morgenthaler TI, Lee-Chiong T, Alessi C, *et al.* Practice parameters for the clinical evaluation and treatment of circadian rhythm sleep disorders. An American Academy of Sleep Medicine report. *Sleep* 2007;**30**:1445–59.

28 Sadeh A. The role and validity of actigraphy in sleep medicine: an update. *Sleep Med Rev* 2011;**15**:259–67. doi:10.1016/j.smrv.2010.10.001

29 Sani M, Refinetti R, Jean-Louis G, *et al.* Daily activity patterns of 2316 men and women from five countries differing in socioeconomic development. *Chronobiol Int* 2015;**32**:650–6.

30 Hart TL, Swartz AM, Cashin SE, *et al.* How many days of monitoring predict physical activity and sedentary behaviour in older adults? *Int J Behav Nutr Phys*

For peer review only - http://bmjopen.bmj.com/site/about/guidelines.xhtml

Act 2011;**8**:62. doi:10.1186/1479-5868-8-62

31 Falck RS, Landry GJ, Brazendale K, *et al.* Measuring Physical Activity in Older Adults Using MotionWatch 8© Actigraphy: How Many Days are Needed? *J Aging Phys Act* Published Online First: 7 June 2016. doi:10.1123/japa.2015-0256

32 Dillon CB, Fitzgerald AP, Kearney PM, *et al.* Number of Days Required to Estimate Habitual Activity Using Wrist-Worn GENEActiv Accelerometer: A Cross-Sectional Study. *PLoS One* 2016;**11**:e0109913. doi:10.1371/journal.pone.0109913

33 Trost SG, Zheng Y, Wong W-K. Machine learning for activity recognition: hip versus wrist data. *Physiol Meas* 2014;**35**:2183–9. doi:10.1088/0967-3334/35/11/2183

34 Ellis K, Kerr J, Godbole S, *et al.* Hip and Wrist Accelerometer Algorithms for Free-Living Behavior Classification. *Med Sci Sports Exerc* 2016;**48**:933–40. doi:10.1249/MSS.0000000000000840

35 Rowlands A V, Cliff DP, Fairclough SJ, *et al.* Moving Forward with Backward Compatibility: Translating Wrist Accelerometer Data. *Med Sci Sports Exerc* Published Online First: 20 June 2016. doi:10.1249/MSS.0000000000001015

36 Koster A, Shiroma EJ, Caserotti P, *et al.* Comparison of Sedentary Estimates between activPAL and Hip- and Wrist-Worn ActiGraph. *Med Sci Sports Exerc* 2016;**48**:1514–22. doi:10.1249/MSS.0000000000000924

37 Haddad SM, Cecatti JG, Parpinelli MA, *et al.* From planning to practice: building the national network for the Surveillance of Severe Maternal Morbidity. *BMC Public Health* 2011;**11**:283. doi:10.1186/1471-2458-11-283

38 Fisher M. A revealing map of the world’s most and least ethnically diverse countries. Washint. Post. 2013.http://www.washingtonpost.com/blogs/worldviews/wp/2013/o5/16/a-revealing-map-of-the-worlds-most-and-least-ethnically-diverse-countries. (accessed 25 Jul 2016).

39 United Nations Development Programme (UNDP). Hum. Dev. Index (HDI). Brazil. 2010.http://www.pnud.org.br/Atlas.aspx

40 Wu J, Sigmund CD. Hypertension: A Disease That Strikes Around the Clock. *Hypertension* 2016;**67**:493–5. doi:10.1161/HYPERTENSIONAHA.115.06331

41 Danilack VA, Nunes AP, Phipps MG. Unexpected complications of low-risk pregnancies in the United States. *Am J Obstet Gynecol* 2015;**212**:809.e1-809.e6. doi:10.1016/j.ajog.2015.03.038

42 Luft CDB, Sanches S de O, Mazo GZ, *et al.* [Brazilian version of the Perceived Stress Scale: translation and validation for the elderly]. *Rev Saude Publica* 2007;**41**:606–15.

43 Pesce RP, Assis SG, Avanci JQ, *et al.* [Cross-cultural adaptation, reliability and validity of the resilience scale]. *Cad Saude Publica*;**21**:436–48. doi:/S0102-311X2005000200010

44 Passini R, Cecatti JG, Lajos GJ, *et al.* Brazilian Multicentre Study on Preterm Birth (EMIP): Prevalence and Factors Associated with Spontaneous Preterm Birth. *PLoS One* 2014;**9**:e109069. doi:10.1371/journal.pone.0109069

For peer review only - http://bmjopen.bmj.com/site/about/guidelines.xhtml

45 Lisonkova S, Joseph KS. Incidence of preeclampsia: risk factors and outcomes associated with early- versus late-onset disease. *Am J Obstet Gynecol* 2013;**209**:544.e1-544.e12. doi:10.1016/j.ajog.2013.08.019

46 Belton S, O'Brien W, Wickel EE, *et al.* Patterns of noncompliance in adolescent field-based accelerometer research. *J Phys Act Health* 2013;**10**:1181–5.

47 Ellis K, Kerr J, Godbole S, *et al.* A random forest classifier for the prediction of energy expenditure and type of physical activity from wrist and hip accelerometers. *Physiol Meas* 2014;**35**:2191–203. doi:10.1088/0967-3334/35/11/2191

48 Staudenmayer J, He S, Hickey A, *et al.* Methods to estimate aspects of physical activity and sedentary behavior from high-frequency wrist accelerometer measurements. *J Appl Physiol* 2015;**119**.

49 Kamada M, Shiroma EJ, Harris TB, *et al.* Comparison of physical activity assessed using hip- and wrist-worn accelerometers. *Gait Posture* 2016;**44**:23–8. doi:10.1016/j.gaitpost.2015.11.005

50 Freedson PS, Melanson E, Sirard J. Calibration of the Computer Science and Applications, Inc. accelerometer. *Med Sci Sports Exerc* 1998;**30**:777–81.

51 Tranquilli AL, Dekker G, Magee L, *et al.* The classification, diagnosis and management of the hypertensive disorders of pregnancy: A revised statement from the ISSHP. *Pregnancy Hypertens* 2014;**4**:97–104. doi:10.1016/j.preghy.2014.02.001

52 WHO recommendations on antenatal care for a positive pregnancy experience. World Health Organization. 2016.http://www.who.int/reproductivehealth/publications/maternal_perinatal_health/anc-positive-pregnancy-experience/en/

53 North RA, McCowan LME, Dekker GA, *et al.* Clinical risk prediction for pre-eclampsia in nulliparous women: development of model in international prospective cohort. *BMJ* 2011;**342**:d1875.

54 Murphy NM, McCarthy FP, Khashan AS, *et al.* Compliance with National Institute of Health and Care Excellence risk-based screening for Gestational Diabetes Mellitus in nulliparous women. *Eur J Obstet Gynecol Reprod Biol* 2016;**199**:60–5. doi:10.1016/j.ejogrb.2016.01.044

55 McCowan LME, Roberts CT, Dekker GA, *et al.* Risk factors for small-for-gestational-age infants by customised birthweight centiles: data from an international prospective cohort study. *BJOG* 2010;**117**:1599–607. doi:10.1111/j.1471-0528.2010.02737.x

56 Thaddeus S, Maine D. Too far to walk: maternal mortality in context. *Soc Sci Med* 1994;**38**:1091–110.

57 von Elm E, Altman DG, Egger M, *et al.* The Strengthening the Reporting of Observational Studies in Epidemiology (STROBE) statement: guidelines for reporting observational studies. *PLoS Med* 2007;**4**:e296. doi:10.1371/journal.pmed.0040296

58 Huberty J, Ehlers DK, Kurka J, *et al.* Feasibility of three wearable sensors for 24 hour monitoring in middle-aged women. *BMC Womens Health* 2015;**15**:55. doi:10.1186/s12905-015-0212-3

For peer review only - http://bmjopen.bmj.com/site/about/guidelines.xhtml

59 Wright SP, Hall Brown TS, Collier SR, *et al.* How consumer physical activity monitors could transform human physiology research. *Am J Physiol Regul Integr Comp Physiol* 2017;:ajpregu.00349.2016. doi:10.1152/ajpregu.00349.2016

60 Herring SJ, Foster GD, Pien GW, *et al.* Do pregnant women accurately report sleep time? A comparison between self-reported and objective measures of sleep duration in pregnancy among a sample of urban mothers. *Sleep Breath* 2013;**17**:1323–7. doi:10.1007/s11325-013-0835-2

61 Okun ML, Kline CE, Roberts JM, *et al.* Prevalence of sleep deficiency in early gestation and its associations with stress and depressive symptoms. *J women's Heal* 2013;**22**:1028–37. doi:10.1089/jwh.2013.4331

62 Tsai S-Y, Lee P-L, Lin J-W, *et al.* Cross-sectional and longitudinal associations between sleep and health-related quality of life in pregnant women: A prospective observational study. *Int J Nurs Stud* 2016;**56**:45–53. doi:10.1016/j.ijnurstu.2016.01.001

63 Herring SJ, Nelson DB, Pien GW, *et al.* Objectively measured sleep duration and hyperglycemia in pregnancy. *Sleep Med* 2014;**15**:51–5. doi:10.1016/j.sleep.2013.07.018

For peer review only

Table 1. Participating centers in the Maternal Actigraphy Exploratory Study I (MAES-I)

• The Maternity of CAISM, University of Campinas, in Campinas, SP, Brazil;<br>○ Local Principal Investigator: Maria Laura Costa.
• Maternity of the Clinic Hospital, Federal University of RS, Porto Alegre, RS, Brazil;<br>○ Local Principal Investigator: Janete Vettorazzi.
• Maternity of the University Hospital, Jundiaí Medical School in Jundiaí, SP, Brazil;<br>○ Local Principal Investigator: Ricardo Porto Tedesco.
• Maternity of Clinic Hospital, Federal University of Pernambuco, in Recife, PE, Brazil;<br>○ Local Principal Investigator: Edilberto A Rocha Filho.
• MEAC – School Maternity of the Federal University of Ceará, in Fortaleza, CE, Brazil.<br>○ Local Principal Investigator: Francisco Edson de Lucena Feitosa.

For peer review only

For peer review only - http://bmjopen.bmj.com/site/about/guidelines.xhtml

Table 2. Inclusion and Exclusion Criteria of MAES – I

Inclusion Criteria

- Singleton pregnancy
- Nulliparous (no previous delivery ≥ 20weeks)
- Between 4+0 – 21+0 weeks of gestation

Exclusion Criteria

- Unsure LMP and unwilling to have to date the Ultrasound
- ≥ 3 Miscarriages
- Major Fetal Anomaly/Abnormal Karyotype*
- Essential Hypertension Treated Pre-pregnancy
- Mod-Severe Hypertension at booking (≥ 160/100 mmHg) or Chronic hypertension using antihypertensive medication
- Pre-pregnancy Diabetes
- Renal Disease
- Systemic Lupus Erythematosus
- Anti-phospholipid Syndrome
- Sickle Cell Disease
- HIV or Hep B or Hep C positive
- Any condition that limits practice of physical activity
- Major Uterine Anomaly
- Cervical Suture
- Knife cone biopsy
- Ruptured membranes
- Use of long-term steroids
- Use of Low-dose Aspirin
- Use of Calcium (> 1g/24h)
- Use of Eicosapentaenoic acid (fish oil) > 2,7g
- Use of Vit. C ≥ 1000mg & Vit. E ≥ 400 UI
- Use of Heparin/LMW Heparin
- Untreated Thyroid disease
- Use of antidepressant and/or anxiolytic agents

* All information regarding fetal anomalies will be properly recorded

For peer review only - http://bmjopen.bmj.com/site/about/guidelines.xhtml

Table 3. Measurement frequency and maximum return periods according to gestational age – MAES I

Gestational age	Measurement frequency	Maximum return period (weeks)
19 – 32 weeks	20Hz	4
33 – 36 weeks	30Hz	2
37 – 42 weeks	50Hz	1

For peer review only

For peer review only - http://bmjopen.bmj.com/site/about/guidelines.xhtml

Table 4. Performance of wrist and hip actigraphy methods according to different activities

	Hip			Wrist		
	**Sens**	**Spec**	**BA**	**Sens**	**Spec**	**BA**
Sitting	0.894	0.923	0.908	0.883	0.870	0.876
Vehicle	0.870	0.987	0.929	0.823	0.964	0.893
Walking/running	0.687	0.981	0.834	0.574	0.983	0.779
Standing	0.797	0.929	0.851	0.687	0.904	0.795
Average	0.812	0.955	0.881	0.742	0.930	0.836

BA: balanced accuracy; sens: sensitivity; spec: specificity

Adapted from Ellis K *et al*. Hip and Wrist Accelerometer Algorithms for Free-Living Behavior Classification. Med. Sci. Sports Exerc. 2016.

For peer review only

For peer review only - http://bmjopen.bmj.com/site/about/guidelines.xhtml

Table 5. Severe Neonatal Morbidity definition according to term/preterm status

**Preterm**	**Term**
Grade III and IV intraventricular haemorrhage	Grade II or III hypoxic ischemic encephalopathy
Chronic lung disease (home on O_2 or on O_2 at 36 weeks gestation	Ventilation>24 hours
Necrotizing enterocolitis	Neonatal intensive care admission >4 days
Retinopathy of prematurity, stage 3 or 4	Apgar score <4 at 5 mins
Sepsis (blood or CSF culture proven)	Cord arterial pH<7.0 and/or base excess>-15
Cystic peri-ventricular leukomalacia	Neonatal seizures

For peer review only

Figure legends:

Figure 1. Set points of MAES-I study

Figure 2. Estimated prevalence of preterm birth and preeclampsia according to gestational age (RED represents majority of cases) and period of evaluation of PA and sleep patterns (in GREEN)

For peer review only

For peer review only - http://bmjopen.bmj.com/site/about/guidelines.xhtml

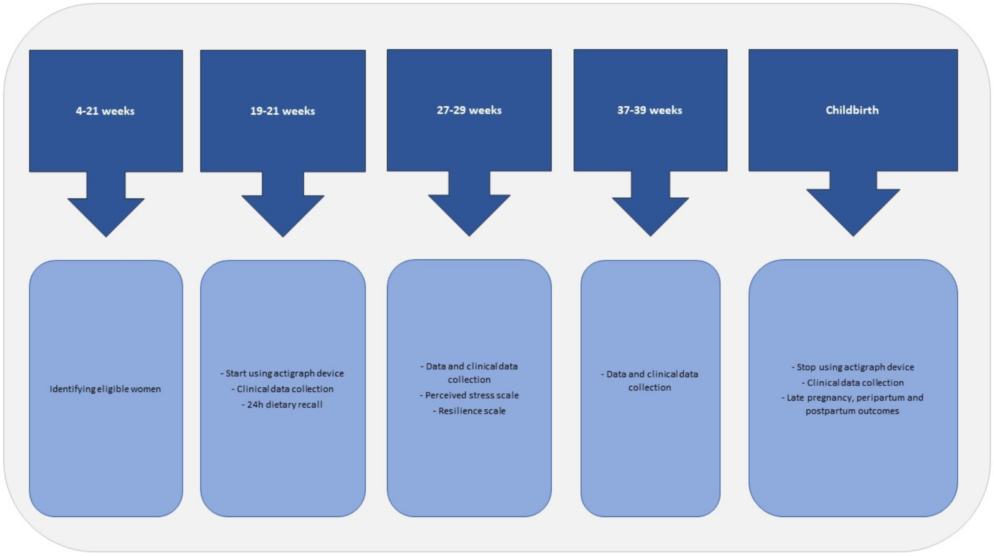

Figure 1

338x190mm (96 x 96 DPI)

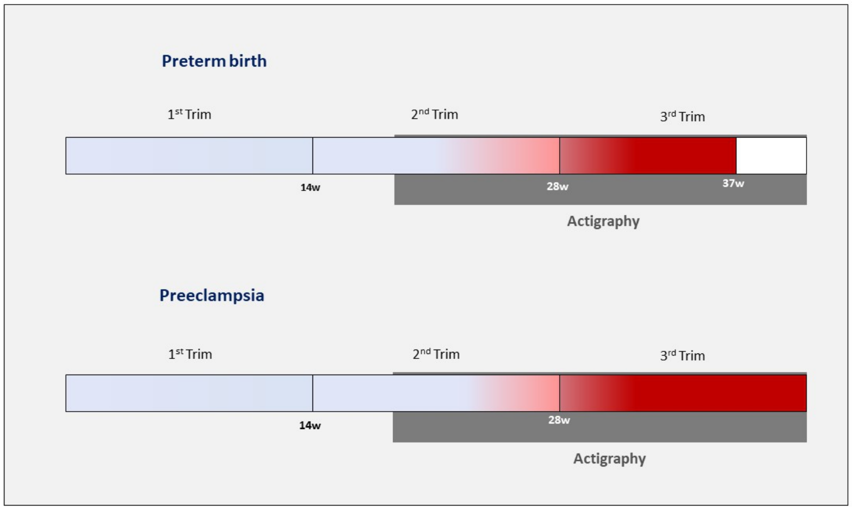

Figure 2

338x190mm (96 x 96 DPI)

BMJ Open

The identification of earlier predictors of pregnancy complications through wearable technologies in a Brazilian multicenter cohort: Maternal Actigraphy Exploratory Study I (MAES-I) study protocol

Journal:	*BMJ Open*
Manuscript ID	bmjopen-2018-023101.R1
Article Type:	Protocol
Date Submitted by the Author:	06-Aug-2018
Complete List of Authors:	Souza, Renato; Universidade Estadual de Campinas, Obstetrics and Gynecology<br>Cecatti, Jose; University of Campinas, Obstetrics and Gynecology<br>Mayrink, Jussara; Universidade Estadual de Campinas Faculdade de Ciencias Medicas<br>Galvão, Rafael; Universidade Estadual de Campinas, Obstetrics and Gynecology<br>Costa, Maria Laura; Universidade Estadual de Campinas, Obstetrics and Gynecology<br>Feitosa, Francisco; Universidade Federal do Ceara, Maternidade Escola<br>Rocha Filho, Edilberto; Universidade Federal de Pernambuco, Obstetrics and Gynecology<br>Leite, Debora; Universidade Federal de Pernambuco, Obstetrics and Gynecology<br>Vettorazzi, Janete; Universidade Federal do Rio Grande do Sul, Obstetrics and Gynecology<br>Tedesco, Ricardo; School of Medicine of Jundiai, Obstetrics and Gynecology<br>Santana, Danielly; Universidade Estadual de Campinas, Obstetrics and Gynecology<br>Souza, Joao Paulo; Universidade de Sao Paulo Faculdade de Medicina de Ribeirao Preto, Social Medicine
**Primary Subject Heading**:	Obstetrics and gynaecology
Secondary Subject Heading:	Obstetrics and gynaecology
Keywords:	wearable technologies, actigraph, physical activity, sleep patterns, pregnancy complications

SCHOLARONE™
Manuscripts

For peer review only - http://bmjopen.bmj.com/site/about/guidelines.xhtml

STUDY PROTOCOL

The identification of earlier predictors of pregnancy complications through wearable technologies in a Brazilian multicenter cohort: Maternal Actigraphy Exploratory Study I (MAES-I) study protocol

Renato T. Souza [1], Jose G. Cecatti [1#], Jussara Mayrink [1], Rafael B. Galvao [1], Maria L. Costa [1], Francisco E. Feitosa [2], Edilberto A Rocha Filho [3], Débora F Leite [1,3], Janete Vettorazzi [4], Ricardo P Tedesco [5], Danielly S Santana [1,5], Joao P. Souza [6], for the MAES-I Study Group*

Affiliations

[1] Department of Obstetrics and Gynecology, University of Campinas (UNICAMP) School of Medical Sciences, Campinas, SP, Brazil

[2] MEAC – School Maternity of the Federal University of Ceará, in Fortaleza, CE, Brazil

[3] Department of Maternal and Child Health, Maternity of Clinic Hospital, Federal University of Pernambuco, in Recife, PE, Brazil

[4] Department of Obstetrics and Gynecology, Maternity of the Clinic Hospital, Federal University of RS, Porto Alegre, RS, Brazil

[5] Department of Obstetrics and Gynecology, Jundiaí Medical School, Jundiaí, SP, Brazil

[6] Department of Social Medicine, Ribeirão Preto Medical School, University of Sao Paulo, Ribeirão Preto, SP, Brazil

*Membership of the MAES-I study group is provided in the Acknowledgments.

E-mail addresses: RT Souza: renatotsouzasp@gmail.com; JG Cecatti: cecatti@unicamp.br; J Mayrink: jussaramayrink@gmail.com; RB Galvão: rafaelbfg@gmail.com; ML Costa: mlaura@unicamp.br; FE Feitosa: edson.lucena@hotmail.com; EA Rocha Filho: edilbertorocha@globo.com; DF Leite: debora.leite@ufpe.br; J Vettorazzi: janetev@terra.com.br; RP Tedesco: rp.tedesco@yahoo.com.br; DS Santana: dany.fmj@terra.com.br; JP Souza: jp.souza@usp.br

#Corresponding Author

JG Cecatti
DO&G, University of Campinas
R. Alexander Fleming, 101
Campinas, SP, 13083-881
Brazil
E-mail: cecatti@unicamp.br

For peer review only - http://bmjopen.bmj.com/site/about/guidelines.xhtml

Abstract

Introduction: non-invasive tools capable of identifying predictors of maternal complications would be a step forward in the improvement of maternal and perinatal health. There is association between modification of physical activity (PA) and sleep-wake patterns and the occurrence of inflammatory, metabolic, pathologic conditions as chronic diseases. The actigraph device is validated to estimate PA and sleep-wake patterns and might be valuable to identify predictors for maternal complications, widening the window of opportunity to prevent, diagnose or treat specific conditions prior to the development of typical symptoms or clinical signs, assessing PA and sleep-wake patterns during pregnancy.

Methods and analysis: A cohort will be held in 5 centres from the Brazilian Network for Studies on Reproductive and Perinatal Health. MAES-I will enroll 400 low-risk nulliparous women that will wear the actigraph device on the wrist during day and night (24h/day) uninterruptedly from 19-21 weeks until childbirth. Changes in PA and sleep-wake patterns through pregnancy will be analyzed, considering gestational age ranges, in women with and without maternal complications during pregnancy, such as preeclampsia, preterm birth (spontaneous and provider-initiated), gestational diabetes, maternal haemorrhage and also perinatal outcomes. A predictive model for screening pregnant women for risk of specific adverse maternal and perinatal outcomes is planned to be then developed using the actigraphy data.

Ethics and Dissemination: MAES-I study has been reviewed and approved by each Institutional Review Board (IRB) and also by the National Council for Ethics in Research. Detailed information of the study is provided in the Brazilian Cohort website (www.medscinet.com/samba) and findings will be publicized in scientific literature and Institutional webpages.

Keywords: wearable technologies; actigraph; physical activity; sleep patterns; sleep-wake cycle; prediction; pregnancy complications.

For peer review only - http://bmjopen.bmj.com/site/about/guidelines.xhtml

Strengths and limitations of this study

- This multicentre cohort will collect comprehensive data on the main maternal and perinatal complications as pre-eclampsia, small for gestational age/fetal growth restriction, preterm birth and gestational diabetes mellitus.
- Physical activity and sleep patterns will be estimated through an innovative wearable device used in the natural environment of the study subject.
- Physical activity and sleep patterns will be estimated from the beginning of the second half of pregnancy until delivery, covering a wide interval during pregnancy and enabling the study of PA and sleep patterns changes throughout pregnancy.
- One possible limitation refers to the uncovered first half of pregnancy regarding this information.

For peer review only

For peer review only - http://bmjopen.bmj.com/site/about/guidelines.xhtml

Background

Reducing the global maternal mortality ratio to less than 70 per 100,000 live births by 2030 is one of the targets of the new United Nations Sustainable Development Goals [1]. Multiple challenges need to be tackled to achieve this target, but the 2016-2030 health and development agenda goes well beyond mortality reduction. The Global Strategy for Women's, Children's and Adolescent's Health aims to ensure that every newborn, women and child not only survive but thrive. This will only be possible if a transformative agenda, with innovation at the central stage, is put into action [2].

One of the major challenges that need to be addressed is optimizing the recognition of earlier predictors and identifiers of maternal and perinatal complications. Delays in diagnosing and managing maternal complications have been associated with poor outcomes [3]. The reduced self-perception of clinical signs related to maternal complications, difficulties in accessing the health system and poor quality of care may contribute to the late identification of complications and worsened prognosis. The development of a non-invasive Antenatal Care (ANC) tool capable of identifying maternal sub-clinical signs during pregnancy may provide the window of opportunity for the earlier identification of abnormal patterns of physiologic parameters related to pregnancy complications. We consider earlier identification when the recognition could be made before clinical presentation, when standard criteria based on clinical signs, symptoms, and supplementary tests are presented. Shortening the time between the onset of a complication and the initiation of the appropriate management allow for secondary prevention and reduction of maternal morbidity and mortality [3–7].

Pervasive computing (i.e. the trend towards embedding microprocessors in everyday-life objects so they can generate data) and wearable technology (i.e. clothing and accessories incorporating computer and advanced electronic technologies such as wrist and/or waistband sensors) are ubiquitous and able to generate a new dataset that needs to be correlated with pregnancy outcomes. Preterm birth and preeclampsia, for instance, are two important pregnancy complications that have a relatively long subclinical phase before the appearance of signs or symptoms [8, 9]. It is plausible that during this subclinical phase of certain conditions the pattern of physical

For peer review only - http://bmjopen.bmj.com/site/about/guidelines.xhtml

activity (PA) or sleep-wake rhythm is affected in some way and this change could be captured through wearable devices. Although some studies show that PA patterns (actigraphy parameters) may be related to systemic inflammation and diseases in the general population [10, 11], published literature correlating wearable technology data and maternal complications are very scarce.

The human circadian rhythm is ruled by endogenous physiologic mechanisms and environmental stimuli [12]. There is solid evidence showing that modification of circadian rhythm or sleep and PA patterns are an underlying condition related to inflammatory, degenerative and/or metabolic chronic diseases as diabetes, hypertension, and cancer [13]. Circadian misalignment is defined as having inappropriate timed sleep and wake, misplaced feeding periods and modification of activity behavior.

The determination of cause or consequence effect between these modifications and the development of pathological conditions is a complex task. It seems that changes in appetite-stimulating hormones, glucose metabolism, inflammatory markers, and mood are some of the related pathways [13–15]. Leproult *et al.* evaluated the effect of circadian misalignment on metabolic and inflammation markers in cardiovascular disease [15]. The insulin action and release, and also the levels of some inflammatory markers that are predictors for cardiovascular diseases, were abnormal in individuals with circadian misalignment. The mechanisms involved in the association between changes of PA pattern and pathologic conditions seem to be of multiple etiologies. Sani *et al.* assessed the circadian rithm of more than 2,300 African descendant adults. More than evaluating physical activity itself, the authors aimed to identify chronobiologic patterns of adults from different socioeconomic settings. The study identified that chronobiologic behavior can vary depending on individual BMI, socioeconomic background, work type and time of sunlight exposure. Possibly, many other factors are involved in modifications of chronobiologic behavior, such as pathologic conditions. Some metabolic, cognitive, cardiovascular and other chronic degenerative diseases have been associated with particular patterns of PA and sleep [10, 11, 16–18]. A previous observational study assessed various sleep parameters during pregnancy, e.g. sleep onset latency (SOL), wake after sleep onset (WASO) and total nocturnal sleep

For peer review only - http://bmjopen.bmj.com/site/about/guidelines.xhtml

time (TST). Difficulty in initiating sleep in early pregnancy was associated with higher body mass index, greater weight gain and higher blood pressure during pregnancy [17]. Palagini *et al.* reviewed clinical evidence between chronic sleep loss and pregnancy adverse outcomes, discussing common mechanisms of stress system activation [19]. Low-quality evidence suggests an association between sleep loss and prenatal depression, gestational diabetes, preeclampsia, abnormal length of labour, caesarean delivery, abnormal fetal growth, and preterm birth. Those results corroborate with other findings regarding pregnancy and sleep disorders [20–23].

The assessment of PA and the sleep patterns can be performed using small wrist (or waist) devices similar to a regular watch (actigraphy technology). The type of sensor, batteries, materials and output data have been substantially developed in recent years, enabling low cost, comfort, discretion and performance [24]. Nowadays there are devices that are portable, lightweight and with a large capacity to storage information, including a software with automatic scoring algorithms packages for the detection of wakefulness, sleep periods and PA [24, 25]. The actigraphy estimation of PA and sleep patterns is validated as a proxy for chronobiologic behavior [26–29] and 7 to 14 days using the actigraph device provides reliable estimates of PA behavior in older adults [30–32]. Both hip and wrist devices show reliable and acceptable performance in estimating PA and sleep-wake patterns [33–36].

The main advantages of using wearable devices for actigraphy is the non-invasiveness, 24/7 monitoring of the circadian pattern and PA, and informing sleep habits and parameters in the user natural environment [24, 25, 28]. We propose an innovative and strategic approach to monitor PA and sleep-wake patterns during pregnancy, establishing a large database comprised of clinical, epidemiological, PA and sleep-wake variables potentially capable of composing a prediction model for maternal complications during pregnancy. The main goal of this study is to identify earlier predictors of pregnancy complications by correlating data generated on PA and sleep patterns through wearable devices (wristband sensors) with maternal and perinatal complications and outcomes.

Methods/Design

For peer review only - http://bmjopen.bmj.com/site/about/guidelines.xhtml

Study design

We will conduct a cohort study of 400 low-risk pregnant women using wrist sensor bands able to capture information on daily physical activity and sleep patterns (exposure). This cohort study will be implemented in 5 ANC clinics linked to obstetric units in 3 different regions of Brazil that are already part of the Brazilian Network for Studies on Reproductive and Perinatal Health [37], as shown in Table 1. During a period of eight months, the ANC clinics will identify eligible cases for using the wristband sensors. Wearable technology data will be correlated with the occurrence of pregnancy and childbirth complications and outcomes, thus understood as an effect.

Eligible women will be identified up to 21 weeks of gestation and invited to participate. A proper consent form will be applied and the women who agree to participate will receive a wristband sensor to be used starting at 19-21 weeks until childbirth, uninterruptedly.

Study setting and population

Brazil is a multi-ethnic mixed-race population of diverse resourced settings [38]. Despite the considered high global overall human development index (HDI 0.727) in 2010, the HDI of Brazilian municipalities ranged from 0,862 to 0,418 [39]. The possibility of considering such mixed population is suitable to explore information regarding maternal patterns of mobility and sleep, maximizing external validity and comparisons to other populations. A few reasons might support the study population focused in low-risk nulliparous women: 1) Previous obstetric history can refer to known risk factors for many maternal complications such as preterm birth, preeclampsia, and diabetes [13, 40]. Nulliparous women enable unbiased sampling regarding obstetric history. 2) Women with previous morbidity such as hypertension, diabetes, nephropathy or others chronic/degenerative diseases are more likely to present abnormalities of sleep-wake rhythm or physical activity patterns during pregnancy.

Sampling

For peer review only - http://bmjopen.bmj.com/site/about/guidelines.xhtml

The five participating centers are regional referral obstetric units responsible for antenatal care assistance mainly for high-risk pregnant women. Participating centers are listed in Table 1. Nevertheless, there are primary health care units strategically linked with these participating centers, enabling the identification and enrollment of low-risk pregnant women. The recruitment strategies include approaching all eligible women in these participating centers and their linked facilities. An informed consent form will be applied for women who agree to participate.

Eligible women: Low-risk pregnant subjects

There is no international consensus on the criteria for low-risk pregnancies, although there are several known factors associated with maternal and perinatal adverse outcomes. A recent study evaluating complications of "low-risk" pregnancies of US Americans (10 million births from 2011 through 2013) showed that 29% of low-risk women had an unexpected complication requiring no routine obstetric/neonatal care [41]. This shows the difficulty in establishing a "low-risk profile" for maternal/perinatal complications. As an exploratory study, we tried to exclude potential known confounders of pre-pregnancy conditions related to adverse maternal or perinatal outcomes as shown in Table 2, in order to assess PA and sleep patterns of a mostly "normal" population. Nonetheless, lifestyle habits and body composition (Body mass index, height, etc.) characteristics, and some non-severe chronic diseases as non-severe anaemia and/or asthma are not among exclusion criteria but may be part of subgroup analyses (composition of any previous disorder, e.g.). Intra and inter-individual analyses of PA and sleep patterns enable the identification of potential confounders affecting primary outcomes, avoiding potential biases. It means that comparison of PA and sleep pattern parameters collected in different stages of pregnancy from the same participant (intra-individual analysis) and collected at the same stage of pregnancy from different participants (inter-individual analysis) will be carried out.

Eligible women will be enrolled between 04-21 weeks. The inclusion and exclusion criteria are presented in Table 2.

Data collection methods

For peer review only - http://bmjopen.bmj.com/site/about/guidelines.xhtml

Essentially, MAES I study is comprised of 4 key set points – 3 clinical visits during pregnancy and a postnatal visit. The clinical visits will be held at 1) between 19-21 weeks; 2) between 27-29 weeks; 3) between 37-39 weeks. At first, second, third and postnatal visits, additional information regarding maternal history, details on pregnancy complications, maternal biophysical data (weight, height, skinfolds) and pregnancy adverse outcomes will be collected following a specific Standard Operating Procedure (SOP) specially developed for MAES-I study. Additionally, the Perceived Stress Scale [42] and Resilience Scale [43] will be applied during 27-29w visit. Figure 1 shows the set points of MAES-I study.

Eligible women will be invited to use a small wrist device similar to a regular watch (GENEActiv Original – Activinsights®). The device contains sensors that estimate PA and sleep-awake patterns through a proper software algorithm. At the first set point of MAES-I study (between 19+0 - 21+0 weeks of gestation), eligible women who agreed to participate will start using the wrist bracelet device on the non-dominant arm during day and night (24h/day) uninterruptedly until childbirth (including bathing or aquatic activities). The acquisition of actigraphy data can be performed in different frequencies (from 10Hz to 100Hz). Since the frequency of data acquisition impacts on the battery life of the device (inverse relationship), the measurement frequency will be set up according to the participant´s gestational age (Table 3). This information will be registered in the database accordingly. The data accumulated will be downloaded during participant´s antenatal care visits, according to the maximum return periods showed in Table 3. The maximum return periods were calculated taking into consideration the expected battery life. At each antenatal care visit, the used device will be returned to the research team and a new charged device will be provided to the participant.

A leaflet with detailed information and FAQ (Frequently asked questions) on the device will also be provided to the women. They will also have a cell phone number to call whether doubts arise regarding the procedures for using the device, or if any technical or medical concern arises.

All actigraphy data collected will be entered into proper software to interpret data and generate an output file. Then, the actigraphy data will be uploaded to an online

For peer review only - http://bmjopen.bmj.com/site/about/guidelines.xhtml

database platform developed by MedSciNet®, where all clinical data of the study will also be registered. The actigraphy software uses several algorithms to estimate physical activity and sleep patterns. The database in centralized, secure, internet-based and allows several procedures for prospective and retrospective monitoring, hierarchical access (local user, general manager, etc.). The database will be translated into Portuguese and English, facilitating data collection for Portuguese-speaking team and international monitoring. A correspondent paper form will be available for data collection if necessary (e.g. internet connection failure for instance).

Decision to start monitoring PA and sleep patterns between 19-21weeks

There are various underlying mechanisms involved in the development of the maternal and perinatal adverse outcomes that will be assessed, as preterm birth, preeclampsia, gestational diabetes, fetal growth restriction and small for gestational age. The pre-clinical phase, stage where there are no clinical signs or symptoms, might be different for each disease and dependent on environmental and individual aspects. The study of adverse maternal and perinatal predictors has been focused in early pregnancy so far (first trimester, <14 weeks of gestation), aiming to maximize the window of opportunity for preventative interventions. However, we hypothesized that the modification of PA and/or sleep pattern due to maternal underlying changes of biological function might not be evident at a very early stage in pregnancy before the beginning of the pre-clinical phase. Our hypothesis is that it possibly occurs shortly before symptoms.

Additionally, we took into account that the occurrence of the main maternal complications, as preeclampsia, fetal growth restriction, and preterm birth, are more common in late pregnancy to establish the period between 19-21 weeks as appropriate to start the assessment of PA and sleep patterns. A recent cross-sectional study conducted in 20 referral centres in Brazil, including the five participating centres of this proposal, showed that the occurrence of preterm birth before 28 weeks comprised less than 1% of all births and less than 8% of all preterm births [44]. In addition, the early onset of preeclampsia (before 34 weeks of gestation) complicates less than 0.4% of all pregnancies, according to a large retrospective cohort of more than 450,000 deliveries in USA [45]. Figure 2 outlines the predicted prevalence of

For peer review only - http://bmjopen.bmj.com/site/about/guidelines.xhtml

preterm birth and preeclampsia in the second trimester, highlighting its clinical presentation, when classic symptoms and signs of a certain disease/complication are presented, through pregnancy in red. Our hypothesis is that PA and sleep patterns might be altered closely to the clinical presentation, still in preclinical phase when there is no symptoms or signs.

In brief, as an exploratory study, we indeed needed to make an arbitrary decision regarding interval of monitoring PA and sleep patterns. For that, we had taken into consideration: 1) the main maternal/perinatal complications of interest occur in the second half of pregnancy, more precisely in late pregnancy (Figure 2); 2) we hypothesize that any potential change on PA or sleep patterns might occur days or weeks before the onset of maternal or perinatal complication. Then, we focused monitoring women during second half of pregnancy.

Thus, the start of assessment between 19-21 weeks seems to be very reasonable, providing a wide interval to monitor and predict the main maternal and perinatal adverse outcomes.

Actigraph device

The actigraph device that will be used to monitor PA and sleep-wake patterns is GENEActiv Original (GENEActiv, Activinsights Ltd, Huntingdon, UK). The device has multiple sensors as microelectromechanical (MEMS) accelerometer, temperature (linear active thermistor) and light (silicon photodiode), providing crude raw data for a variety of applications.

Wrist vs waist wear: advantages and performance

Wrist wear of actigraph devices provides more comfortable use during wake and sleep periods and highest wear time compared to waist monitors [33, 46]. A non-systematic review published in 2011 showed that actigraphy is a useful and reliable tool to assess sleep patterns and circadian rhythm disorders, although there are some limitations on diagnosing sleep diseases or measuring sleep stages [25]. Actigraphy showed excellent concordance with polysomnography in assessing sleep parameters in healthy subjects (sensitivity >90% in estimating total sleep time, for instance). A recent study evaluated the concordance of physical activity estimation of wrist device in free-living settings in

For peer review only - http://bmjopen.bmj.com/site/about/guidelines.xhtml

forty overweight or obese women [34]. They used both wrist and hip devices, and a small camera that captured participant behaviour for 7 days, enabling the monitoring of physical activity behaviour (gold-standard comparison). The hip and wrist machine learning (ML) classifiers used are different due to the different methods/algorithms to estimate physical activity [34]. The sensitivity and specificity of hip and wrist estimations according to Ellis *et al* are showed in Table 4 [34].

Two years ago, the same author had published a similar evaluation using 40 adults (women and men), showing that the hip and wrist accelerometers obtained an average accuracy of 92.3% and 87.5%, respectively, in predicting PA types [47].

Staudenmayer *et al* developed an investigation with 20 participants also using two devices (wrist- and hip- worn), and showed that wrist actigraphy can estimate energy expenditure accurately and relatively precisely [48]. Another study evaluating PA patterns in a free-living environment with wrist devices showed that women in the top 40% or bottom 40% of the distribution of daily PA, hip and wrist accelerometers agreed on the classification for about 75% of the women [49]. Additionally, the total activity (counts per day) was moderately correlated (Spearman's r = 0.73) between the wrist and hip worn devices.

At the best of our knowledge, there are no systematic reviews or other high-quality evidence-based recommendation supporting a particular method. Although wrist wear of actigraphy is not the more traditional method, it might be the best choice for assessing long periods of PA or sleep patterns, even more considering the similar performance of the waist wear. The current proposal does not intend to diagnose pathologic behaviours or diseases, but to identify different patterns along pregnancy and in different subgroups of women. Therefore, supported by the evidence that wrist wear of actigraphy devices can accurately and more comfortably estimate PA and sleep patterns, mainly for long periods and in the free-living environment, the MAES-I study group addopted wrist wear devices.

Main variables

The independent variables assessed as potential predictors of maternal complications will be related to sleep-wake cycle and mobility as:

For peer review only - http://bmjopen.bmj.com/site/about/guidelines.xhtml

"Sleep" variables

- Sleep onset latency (SOL): time elapsed between full wakefulness to sleep.
- Total sleep time (TST): The amount of actual sleep time in a sleep episode (excludes awakes).
- Wake after sleep onset (WASO): defined as total amount of time awake after sleep.
- Sleep Efficiency (SE): the proportion of sleep in the period potentially filled by sleep-ratio of total sleep time to time in bed.

The actigraph device collects many pieces of information related to body position and body movements to estimate the described sleep variables. Then, actigraphy software will be used to analyse the data and generates the output variables.

"Physical activity" variables

Actigraphy technology estimates physical activity through various parameters collected by the actigraph device. Briefly, according to Freedson *et al*, the triaxial sensors stressed by acceleration forces can estimate the intensity of movements. The acceleration signal is converted to digital signal and summed over a user specified time interval (epoch). At the end of each epoch the activity count is stored. Then, according to Count per minute (CPM) cut points, the PA intensity can be categorized [50]. The information is translated by the software using proper algorithms into quantitative variables as following:

- Sedentary time (hours/day): the number of hours per day of count per minute between 0-99.
- Light activity (hours/day): the number of hours per day having count per minute between 100 - 1951.
- Moderate activity (minutes/day): the number of hours per day having count per minute between 1952 - 5724.
- Vigorous activity (minutes/day): the number of hours per day having count per minute between 5725 - 9498.
- Very vigorous activity (minutes/day): the number of hours per day having count per minute between 9499 - ∞.

For peer review only - http://bmjopen.bmj.com/site/about/guidelines.xhtml

- MET rates: Metabolic Equivalents (METs) are commonly used to also express the intensity of physical activities. One MET is the energy cost of resting quietly, often defined in terms of oxygen uptake as 3.5 $mL \cdot kg^{-1} \cdot min^{-}1$. MET rate expresses a person's working metabolic rate relative to their resting metabolic rate. Briefly, the triaxial piezoelectric sensors stressed by acceleration forces can estimate the intensity of movements, converted to the oxygen consumption required to perform such movement.
- Step counts/day: estimated steps count per day (estimated by proper algorithms using accelerometer data.)

Outcomes

The primary outcomes are late pregnancy complications as:

- Preeclampsia: Hypertension after 20 weeks of gestation, systolic BP ≥ 140mmHg and/or diastolic BP ≥ 90mmHg (Korotkoff V) on at least 2 occasions 4h apart with: 1) Proteinuria 300 mg/24h or spot urine protein: creatinine ratio 30 mg/mmol creatinine or urine dipstick protein ≥ (+) OR, in the absence of proteinuria, hypertension and 2) any multi-system complication that are: Haematological abnormalities; thrombocytopenia (platelets < 100 x 10^9/L); Disseminated intravascular coagulation (DIC), and haemolysis; Liver disease: increased aspartate transaminase and/or alanine transaminase > 45 IU/L and/or severe right upper quadrant or epigastric pain, liver rupture; Neurological problems: eclampsia, imminent eclampsia (severe headache with hyperreflexia and persistent visual disturbance), cerebral haemorrhage; Acute renal insufficiency: new increase in serum creatinine to > 100 mmol/L antepartum or > 130 mmol/L postpartum; Pulmonary oedema confirmed by chest x-ray [51].
- Gestational Diabetes: new diabetes developing in pregnancy according to the WHO recommendation [52] that defines gestational diabetes as having:
    - Fasting plasma glucose ≥ 92 mg/dl, or
    - 1-h plasma glucose tolerance test (75g load) ≥ 180 mg/dl, or
    - 2-h plasma glucose tolerance test (75g load) ≥ 153 mg/dl.

For peer review only - http://bmjopen.bmj.com/site/about/guidelines.xhtml

- Spontaneous Preterm Birth: spontaneous onset of preterm labour or premature rupture of membranes leading to preterm birth, childbirth before 37 weeks of gestation.
- Provider-Initiated Preterm Birth: defined as childbirth occurring at less than 37 weeks, medically indicated due to maternal/fetal compromise or both;
- Maternal Hemorrhage: Classified in 1) Antepartum haemorrhage defined as bleeding from the genital tract after 24 weeks of gestation; 2) Primary Postpartum haemorrhage as the loss of 500 ml blood or more from the genital tract within 24 hours of the childbirth.

Secondary outcomes include childbirth variables and neonatal adverse outcomes as fetal death, caesarean section, small for gestational age (defined as birth weight below percentile 10 for gestational age), Apgar score < 7 at 5 minutes, neonatal severe morbidity (Table 5) and neonatal mortality before discharge.

Plans for analyses

Sample size estimation

This is an exploratory and innovative study focused on a specific population (pregnant women) and therefore there are no previously published parameters available for sample size estimation. Considering a relatively wide range of frequency of complications arising in pregnancy from 3 to 20% (including preeclampsia, fetal growth restriction, gestational diabetes, hemorrhage, preterm birth, etc.), a theoretical large population size (above 1 million pregnant women), an acceptable margin of error of 4%, the involvement of 5 clusters (participating centers) and a 95% level of confidence, 384 women would be necessary. Therefore, we are rounding up this estimation for at least 400 initially low-risk pregnant women to be enrolled in the study. We estimated the incidence of some main maternal complications considering the following studies:

- Pre-eclampsia: An international prospective cohort study with nulliparous women called SCOPE used similar criteria for the low-risk profile, having 5% of incidence of pre-eclampsia [53].

For peer review only - http://bmjopen.bmj.com/site/about/guidelines.xhtml

- Preterm Birth: A recent cross-sectional study conducted in 20 referral obstetric centres in Brazil, including the five participant centres of this proposal, showed a prevalence of 12.3% of all births [44].
- Gestational Diabetes: SCOPE international cohort, previously mentioned, had a prevalence of 8.9% of gestational diabetes in low-risk screened nulliparous women, according to mainly to the NICE guidelines [54].
- Fetal growth restriction/small for gestational age: SCOPE international cohort, previously mentioned, had a prevalence of 10.7% of newborns small for gestational age, according to the customized centiles of birthweight (<10%)[55].

Analyzes and statistics details

According to these studies above, the predicted incidence of these complications seems reasonable and reproducible in our cohort. Then, sample size estimation might assure enough cases of maternal and perinatal complications for the current proposal.

Firstly, we will identify PA and sleep-wake patterns of women who did not develop adverse maternal or perinatal outcomes. It will permit the recognition of a normal PA and sleep-wake patterns in low-risk population without complication during pregnancy. Using the same population, we will analyze changes in PA and sleep-wake patterns through pregnancy, allowing for gestational age periods.

Then, we will compare the PA and sleep-wake patterns of women who developed specific adverse maternal or perinatal outcomes with those who did not. The differences between groups might be identified to be used as potential markers for specific pregnancy complications.

After that, we will analyze changes in PA and sleep-wake patterns of women who developed adverse maternal or perinatal outcomes through pregnancy, comparing the patterns and trying to discover which changes and when before the onset it would be related to pregnancy complications. If possible, we will conduct subgroup analysis including subpopulation with potential higher risk for maternal complications (confounder variabels), inclusing obesity, smoking, etc.

For peer review only - http://bmjopen.bmj.com/site/about/guidelines.xhtml

Finally, we will develop a predictive model for screening pregnant women for risk of specific adverse maternal and perinatal outcomes using PA and sleep-wake data estimated with actigraphy technology.

The analysis will be performed using the actigraph software that translates the collected information into PA and sleep-wake parameters. Additionally, Friedman and Wilcoxon for paired samples, t-test, and ANOVA for repeated measures will be applied to achieve statistical analyses. Also, we will address sensitivity, specificity and likelihood ratio for altered PA and sleep patterns or for their changes throughout pregnancy.

Discontinuation of participants

The criteria for discontinuation include:

- Withdrawal of consent;
- Not regularly using the actigraph device for long periods, less than 50% of all planned time. The information that they are not using the device properly will be recorded if women notice the MAES-I team. Otherwise, the low use of the device will be noticed after data discharge during antenatal care visits.
- The loss to follow-up, not allowing the download of actigraphy data.

Data and Sample Quality

All entered data will be prospectively and retrospectively monitored by local research assistants and a global monitor. Internal consistency of variables will be constantly performed by the database and error messages are automatically flagged. A local research assistant will be responsible for checking all forms and actigraphy data before locking forms, assuring good quality of data (double-checking entered data and checking for inconsistencies between variables, for instance). Then, the local principal investigator (PI) will be responsible for signing the case, enabling its incorporation to the final database. The University of Campinas will coordinate, implement and monitor the study in the five participating centres. A general manager and a global monitor are also part of the team of the coordinating centre. The local team of each participating centre is comprised of a Local PI and research assistants.

Ethics and Dissemination

For peer review only - http://bmjopen.bmj.com/site/about/guidelines.xhtml

MAES-I study focuses on low-risk nulliparous Brazilian pregnant women. Despite being considered of low risk for maternal and perinatal complications, they are not free of suffering complications. Furthermore, the first and second delays, defined as a delay in deciding to seek care and delay in reaching a health care facility [56], are not uncommon, establishing a barrier between earlier recognition of symptoms and timely interventions capable to successfully treat potentially life-threatening conditions. We believe that women will feel encouraged, empowered and willing to participate in the study that aims to develop a potentially useful prenatal care tool to identify the risk for maternal and perinatal morbidity and mortality. Following national ethical regulations, the participants will not receive any financial compensation.

Women who agree to participate in the study will not have any disadvantages or injury of their prenatal care. On the contrary, they will receive a telephone number to contact the clinical researchers at any time (24/7 service), which enables a closer contact with researchers and providers of care, since the MAES-I team are committed to contacting providers of care if any potential complication is noticed by participants.

The participating women will not be responsible if loss, theft or damage to the wrist device occurs. However, they will be asked to return the device after they finished the participation in the study, in order to use it for new women entering the study. No self-damage is expected in those who use the device.

Participating women will not be able to identify any PA or sleep parameters at any stage of the study. The download of the data is only possible through the own licensed software of the device. Actigraphy devices provided for participating women have a unique code which will be recorded in the database together with the interval of use for each women. Actigraphy data will be labelled using participant ID, device number, gestational age when starting using each device and return date of each device. The use of such codes, ID`s and numbers will ensure confidential identify for all participating women. The identity of all women will be kept confidential.

MAES-I study has been reviewed and approved by the National Committee for Ethics in Research of Brazil (CONEP) and by the Institutional Review Board (IRB) of the coordinating centre (Letter of approval 1.834.116 issued on 24th November 2016) and

For peer review only - http://bmjopen.bmj.com/site/about/guidelines.xhtml

of all other Brazilian participating centres. All women who will be enrolled in the MAES-I cohort will sign an informed consent form.

The ethical principles stated in the Brazilian National Heath Council (Resolution CNS 466/12) will be respected in every stage of this study. The anonymity of the source of information will be guaranteed and the care for the women will be provided independently of her agreement to participate in the study. The study also complies with the Declaration of Helsinki amended in Hong Kong in 1989.The methodological and ethical aspects of MAES-I study protocol were developed following STROBE guidelines [57].

Patient and Public Involvement

Patients and public were not involved in this study for the development of the research question and outcome measures. However, the choice for a wrist device was based on the preference of users as reported. Participants of the study will have access to the results by its webpage that will be open access.

Detailed information of the study is provided in the Brazilian Cohort website (www.medscinet.com/samba) and findings will be publicized in scientific literature and Institutional webpages. We intend to disseminate our findings in scientific peer-reviewed journal, general free access website, specialists' conferences, and to our funding agencies.

Discussion

The actigraphy is an innovative, non-invasive, non-operator dependent, wearable technology, which enables the estimative under real life conditions of diverse variables related to mobility, physical activity, sleep-wake, and circadian cycle patterns. Actigraph devices show high sensitivity in sleep-wake parameters detection and are currently highly recommended by the American Sleep Disorder Association for diagnosis and therapy response of circadian rhythm disorders [27, 28, 58]. Although some studies show that 7 to 14 days using the actigraph device provides reliable estimates of physical activity behavior in older adult, it is not absolutely clear how many days is needed to estimate habitual PA by using the wrist/waist device during

For peer review only - http://bmjopen.bmj.com/site/about/guidelines.xhtml

pregnancy. In general, it seems to depend mainly on the type of actigraph device, position of wear and target population [30, 33]. Nevertheless, MAES-I study will provide sufficient data to assess different patterns along pregnancy.

The use of wearable physical activity monitors has grown enormously due to the interest about the relationship between the pathophysiology of diseases and physical activity and sleep patterns. A recent study on the use of physical activity monitors in human physiology research unravels the current and potential uses of actigraph device as in strategies to promote healthier behaviour or to predict outcomes [59]. The authors conclude that physical activity monitors, as others new 21st century technologies, have already transformed physiology research, revolutionizing the way we assess patients and opening new areas of interest. In addition, the use of objectives measures to evaluate habitual sleep duration and outcomes in pregnancy is critical, taking into account recent investigations reporting little agreement between objective and subjective assessments of sleep time [60].

Alterations in sleep patterns, as less deep sleep and more nocturnal awakenings, can be observed in pregnancy as early as 10-12 weeks gestation [61]. Sleep disturbances during pregnancy have been associated with preterm delivery, gestational hypertensive disorders, glucose intolerance and increased risk of caesarean delivery [19]. Shorter night time sleep was also associated with hyperglycemia [62]. Persistent sleep deficiency is correlated with depressive symptoms and stress perception by pregnant women [61]. These studies lay correlation between PA patterns and sleep disturbances determining complications, in a well-established relationship of cause and consequence, although sometimes it could not be adequately determined due to the study design [17].

In a distinct way, our analysis intends to figure out if the maternal complication could be identified by physical activity and/or sleep patterns modifications, even during its pre-clinical period, previous the appearance of clinical signs. Considering the existing evidence, we speculate that the PA and/or sleep patterns change days or weeks before the clinical presentation of the complication. In general, the signs and symptoms of some maternal outcomes are part of the gold-standard criteria for diagnosis (high blood pressure, proteinuria and/or edema in the case of preeclampsia; premature

For peer review only - http://bmjopen.bmj.com/site/about/guidelines.xhtml

contractions and cervical ripening/dilation in preterm birth; abnormal placental blood flow and insufficient fetal growth in Intrauterine Growth Restriction). We acknowledge the fact that there are potential confounders and limitations in predicting maternal and perinatal complications using PA and sleep patterns estimated by actigraph devices. We expect that our studied population will have different subgroups of women with different risks and associated factors playing a role on maternal complication. It includes obesity, smoking, extremes of age, for instance. None of those factors was considered exclusion criteria and, if possible, we intend to assess subgroup analysis for those maternal subgroups at they might present different PA and sleep patterns. Nonetheless, we decided to perform a pragmatic approach, not excluding such common factor from our sample.

The use of actigraph device during prenatal visits has a potential to become a new tool to monitor pregnant women, improving maternal health care, identifying altered PA and/or sleep patterns, measured objectively through actigraphy, before the occurrence of those signs and symptoms. Therefore, the focus would be offering new technology to monitor the development of a potential maternal complication. Other positive points of our study are the period of data collection (from 19 weeks till delivery) and the low-risk profile of the cohort. Through which, it would be possible to describe a PA and sleep patterns in a low-risk pregnant population and better interpret actigraphy data among pregnant women. The current clinical and biological predictors for the main maternal complications as preeclampsia, preterm birth, maternal haemorrhage, and gestational diabetes still lack for effective sensitivity and specificity.

If this is confirmed to be true, an important step will be achieved for a possible introduction of screening non-invasive procedures during prenatal care with the purpose of identifying women at higher risk of developing those conditions. Therefore, they could receive specific orientation on prevention and earlier detection of the onset of condition for taking immediate action to look for professional health care and receiving appropriate interventions, avoiding delays that are the most striking factor for the low quality of care the women usually receive in low and middle-income settings, contributing to the still high burden of maternal morbidity and mortality. If we were successful in identifying such “specific patterns of physical activity and sleep”

For peer review only - http://bmjopen.bmj.com/site/about/guidelines.xhtml

as predictors for pregnancy complications, further validation studies will necessarily be recommended for assessing its effectiveness for the whole management of such conditions. Additionally, MAES-I will enable further specific studies among high risk population and also will help to identify the best gestational age for monitoring, giving the means to target a specific gestational age interval.

For peer review only

1 **Abbreviations**

ANC – antenatal care

BMI – body mass index

BP – blood pressure

CPM – count per minute

dL – decilitre

FAQ – frequently asked questions

h – hour

HDI – human development index

Hz - hertz

Kg – kilogram

L – litre

MAES-I – maternal Actigraphy Exploratory Study I

MEMS – microelectromechanical

MET – metabolic equivalent

METs – metabolic equivalents

min – minutes

mg – milligram

mL – millilitre

mmol – millimole

NICE – National Institute for Health and Care Excellence

PA – physical activity

PI – Principal investigator

SE – sleep efficiency

SCOPE – SCreening Of Pregnancy Endpoints

SOL – sleep onset latency

TST – total nocturnal sleep time

US – United States

USA – United States of America

w – week

Competing interests

The authors declare that they have no competing interests.

Authors' contributions

All authors contributed to the overall study design and specific methodologies. RTS, JGC, JM, MLC and JPS conceived the study design and wrote de first version of the study protocol. In a first meeting, the protocol was discussed and incorporated suggestions from RBG, FEF, ERF, DFL, JV, RPT and DSS. RTS, JGC, JM, and RBG planned the implementation of the study and developed the necessary material. RTS, JM, MLC and JGC drafted the manuscript that was afterwards revised by all other authors who gave suggestions. All authors discussed and made important contributions to the manuscript, read and approved the final version for submission.

For peer review only

Acknowledgements

The MAES - I study group also included: Carina B Luiz and Luiza C Brust, School of Medicine, Federal University of Rio Grande do Sul, Porto Alegre, Brazil; Danilo Anacleto and Lívia C Nascimento, School of Medicine, Federal University of Pernambuco, Recife, Brazil; Daisy Lucena and Denise Ellen F Cordeiro, School of Medicine, Federal University of Ceará, Fortaleza, Brazil; Mariana B Rogerio, Departament of Obstetrics and Gynecology, Jundiaí Medical School, Jundiaí, Brazil.

Funding

This study was granted by The Bill and Melinda Gates Foundation through the Grand Challenge Exploration program, call 19 (research grant OPP1182749).

For peer review only

References

1. United Nations Development Programme (UNDP). Sustainable Development Goals. 2015. http://www.un.org/sustainabledevelopment/health/. Accessed 27 Apr 2016.

2. WHO, World Health Organization. Global Strategy. Global Strategy for Women's, Children's and Adolescents' Health, 2016 - 2030. 2015. http://www.who.int/life-course/partners/global-strategy/global-strategy-2016-2030/en/. Accessed 9 Jul 2016.

3. Pacagnella RC, Cecatti JG, Parpinelli MA, Sousa MH, Haddad SM, Costa ML, et al. Delays in receiving obstetric care and poor maternal outcomes: results from a national multicentre cross-sectional study. BMC Pregnancy Childbirth. 2014;14:159.

4. Haddad SM, Cecatti JG, Souza JP, Sousa MH, Parpinelli MA, Costa ML, et al. Applying the maternal near miss approach for the evaluation of quality of obstetric care: a worked example from a Multicenter Surveillance Study. Biomed Res Int. 2014;2014:989815.

5. Pacagnella RC, Cecatti JG, Osis MJ, Souza JP. The role of delays in severe maternal morbidity and mortality: expanding the conceptual framework. Reprod Health Matters. 2012;20:155–63.

6. Vogel JP, Souza JP, Mori R, Morisaki N, Lumbiganon P, Laopaiboon M, et al. Maternal complications and perinatal mortality: findings of the World Health Organization Multicountry Survey on Maternal and Newborn Health. BJOG. 2014;121 Suppl:76–88.

7. Souza JP, Gülmezoglu AM, Vogel J, Carroli G, Lumbiganon P, Qureshi Z, et al. Moving beyond essential interventions for reduction of maternal mortality (the WHO Multicountry Survey on Maternal and Newborn Health): a cross-sectional study. Lancet. 2013;381:1747–55.

8. Brosens I, Pijnenborg R, Vercruysse L, Romero R. The "Great Obstetrical Syndromes" are associated with disorders of deep placentation. Am J Obstet Gynecol. 2011;204:193–201.

9. Di Renzo GC. The great obstetrical syndromes. J Matern neonatal Med. 2009;22:633–5.

10. Loprinzi PD. The effects of objectively-measured, free-living daily ambulatory movement on mortality in a national sample of adults with diabetes. Physiol Behav. 2016;154:126–8.

11. Biswas A, Oh PI, Faulkner GE, Bajaj RR, Silver MA, Mitchell MS, et al. Sedentary Time and Its Association With Risk for Disease Incidence, Mortality, and Hospitalization in Adults. Ann Intern Med. 2015;162:123.

12. Hardin PE. From biological clock to biological rhythms. Genome Biol. 2000;1:reviews1023.1-1023.5.

13. Kizaki T, Sato S, Shirato K, Sakurai T, Ogasawara J, Izawa T, et al. Effect of Circadian Rhythm on Clinical and Pathophysiological Conditions and Inflammation. Crit Rev Immunol. 2015;35:261–75.

14. Baron KG, Reid KJ. Circadian misalignment and health. Int Rev Psychiatry. 2014;26:139–54.

15. Leproult R, Holmbäck U, Van Cauter E, McMenamin T, Suwazono Y, Dochi M, et al. Circadian misalignment augments markers of insulin resistance and inflammation,

For peer review only - http://bmjopen.bmj.com/site/about/guidelines.xhtml

independently of sleep loss. Diabetes. 2014;63:1860–9.

16. Diem SJ, Blackwell TL, Stone KL, Yaffe K, Tranah G, Cauley JA, et al. Measures of Sleep-Wake Patterns and Risk of Mild Cognitive Impairment or Dementia in Older Women. Am J Geriatr Psychiatry. 2016;24:248–58.

17. Haney A, Buysse DJ, Rosario BL, Chen Y-F, Okun ML. Sleep disturbance and cardiometabolic risk factors in early pregnancy: a preliminary study. Sleep Med. 2014;15:444–50.

18. Lynch BM, Boyle T, Winkler E, Occleston J, Courneya KS, Vallance JK. Patterns and correlates of accelerometer-assessed physical activity and sedentary time among colon cancer survivors. Cancer Causes Control. 2016;27:59–68.

19. Palagini L, Gemignani A, Banti S, Manconi M, Mauri M, Riemann D. Chronic sleep loss during pregnancy as a determinant of stress: impact on pregnancy outcome. Sleep Med. 2014;15:853–9.

20. Tsai S-Y, Lin J-W, Wu W-W, Lee C-N, Lee P-L. Sleep Disturbances and Symptoms of Depression and Daytime Sleepiness in Pregnant Women. Birth. 2016;43:176–83.

21. Tsai S-Y, Lee P-L, Lin J-W, Lee C-N. Cross-sectional and Longitudinal Associations between Sleep and Health-related Quality of Life in Pregnant Women: a Prospective Observational Study. Int J Nurs Stud. 2016;56:45–53.

22. Hayase M, Shimada M, Seki H. Sleep quality and stress in women with pregnancy-induced hypertension and gestational diabetes mellitus. Women Birth. 2014;27:190–5.

23. Mindell JA, Cook RA, Nikolovski J. Sleep patterns and sleep disturbances across pregnancy. Sleep Med. 2015;16:483–8.

24. JOHN D, FREEDSON P. ActiGraph and Actical Physical Activity Monitors. Med Sci Sport Exerc. 2012;44 1 Suppl 1:S86–9.

25. Martin JL, Hakim AD. Wrist actigraphy. Chest. 2011;139:1514–27.

26. Wood AC, Kuntsi J, Asherson P, Saudino KJ. Actigraph data are reliable, with functional reliability increasing with aggregation. Behav Res Methods. 2008;40:873–8.

27. Morgenthaler TI, Lee-Chiong T, Alessi C, Friedman L, Aurora RN, Boehlecke B, et al. Practice parameters for the clinical evaluation and treatment of circadian rhythm sleep disorders. An American Academy of Sleep Medicine report. Sleep. 2007;30:1445–59.

28. Sadeh A. The role and validity of actigraphy in sleep medicine: an update. Sleep Med Rev. 2011;15:259–67.

29. Sani M, Refinetti R, Jean-Louis G, Pandi-Perumal SR, Durazo-Arvizu RA, Dugas LR, et al. Daily activity patterns of 2316 men and women from five countries differing in socioeconomic development. Chronobiol Int. 2015;32:650–6.

30. Hart TL, Swartz AM, Cashin SE, Strath SJ. How many days of monitoring predict physical activity and sedentary behaviour in older adults? Int J Behav Nutr Phys Act. 2011;8:62.

31. Falck RS, Landry GJ, Brazendale K, Liu-Ambrose T. Measuring Physical Activity in Older Adults Using MotionWatch 8© Actigraphy: How Many Days are Needed? J Aging Phys Act. 2016.

For peer review only - http://bmjopen.bmj.com/site/about/guidelines.xhtml

32. Dillon CB, Fitzgerald AP, Kearney PM, Perry IJ, Rennie KL, Kozarski R, et al. Number of Days Required to Estimate Habitual Activity Using Wrist-Worn GENEActiv Accelerometer: A Cross-Sectional Study. PLoS One. 2016;11:e0109913.

33. Trost SG, Zheng Y, Wong W-K. Machine learning for activity recognition: hip versus wrist data. Physiol Meas. 2014;35:2183–9.

34. Ellis K, Kerr J, Godbole S, Staudenmayer J, Lanckriet G. Hip and Wrist Accelerometer Algorithms for Free-Living Behavior Classification. Med Sci Sports Exerc. 2016;48:933–40.

35. Rowlands A V, Cliff DP, Fairclough SJ, Boddy LM, Olds TS, Parfitt G, et al. Moving Forward with Backward Compatibility: Translating Wrist Accelerometer Data. Med Sci Sports Exerc. 2016.

36. Koster A, Shiroma EJ, Caserotti P, Matthews CE, Chen KY, Glynn NW, et al. Comparison of Sedentary Estimates between activPAL and Hip- and Wrist-Worn ActiGraph. Med Sci Sports Exerc. 2016;48:1514–22.

37. Haddad SM, Cecatti JG, Parpinelli MA, Souza JP, Costa ML, Sousa MH, et al. From planning to practice: building the national network for the Surveillance of Severe Maternal Morbidity. BMC Public Health. 2011;11:283.

38. Fisher M. A revealing map of the world's most and least ethnically diverse countries. Washinton Post. 2013. http://www.washingtonpost.com/blogs/worldviews/wp/2013/o5/16/a-revealing-map-of-the-worlds-most-and-least-ethnically-diverse-countries. Accessed 25 Jul 2016.

39. United Nations Development Programme (UNDP). Human Development Index (HDI). Brazil. 2010. http://www.pnud.org.br/Atlas.aspx.

40. Wu J, Sigmund CD. Hypertension: A Disease That Strikes Around the Clock. Hypertension. 2016;67:493–5.

41. Danilack VA, Nunes AP, Phipps MG. Unexpected complications of low-risk pregnancies in the United States. Am J Obstet Gynecol. 2015;212:809.e1-809.e6.

42. Luft CDB, Sanches S de O, Mazo GZ, Andrade A. [Brazilian version of the Perceived Stress Scale: translation and validation for the elderly]. Rev Saude Publica. 2007;41:606–15.

43. Pesce RP, Assis SG, Avanci JQ, Santos NC, Malaquias J V, Carvalhaes R. [Cross-cultural adaptation, reliability and validity of the resilience scale]. Cad Saude Publica. 21:436–48.

44. Passini R, Cecatti JG, Lajos GJ, Tedesco RP, Nomura ML, Dias TZ, et al. Brazilian Multicentre Study on Preterm Birth (EMIP): Prevalence and Factors Associated with Spontaneous Preterm Birth. PLoS One. 2014;9:e109069.

45. Lisonkova S, Joseph KS. Incidence of preeclampsia: risk factors and outcomes associated with early- versus late-onset disease. Am J Obstet Gynecol. 2013;209:544.e1-544.e12.

46. Belton S, O'Brien W, Wickel EE, Issartel J. Patterns of noncompliance in adolescent field-based accelerometer research. J Phys Act Health. 2013;10:1181–5.

47. Ellis K, Kerr J, Godbole S, Lanckriet G, Wing D, Marshall S, et al. A random forest classifier for the prediction of energy expenditure and type of physical activity from wrist and hip accelerometers. Physiol Meas. 2014;35:2191–203.

For peer review only - http://bmjopen.bmj.com/site/about/guidelines.xhtml

48. Staudenmayer J, He S, Hickey A, Sasaki J, Freedson P. Methods to estimate aspects of physical activity and sedentary behavior from high-frequency wrist accelerometer measurements. J Appl Physiol. 2015;119.

49. Kamada M, Shiroma EJ, Harris TB, Lee I-M. Comparison of physical activity assessed using hip- and wrist-worn accelerometers. Gait Posture. 2016;44:23–8.

50. Freedson PS, Melanson E, Sirard J. Calibration of the Computer Science and Applications, Inc. accelerometer. Med Sci Sports Exerc. 1998;30:777–81.

51. Tranquilli AL, Dekker G, Magee L, Roberts J, Sibai BM, Steyn W, et al. The classification, diagnosis and management of the hypertensive disorders of pregnancy: A revised statement from the ISSHP. Pregnancy Hypertens. 2014;4:97–104.

52. WHO recommendations on antenatal care for a positive pregnancy experience. World Health Organization. 2016. http://www.who.int/reproductivehealth/publications/maternal_perinatal_health/anc-positive-pregnancy-experience/en/.

53. North RA, McCowan LME, Dekker GA, Poston L, Chan EHY, Stewart AW, et al. Clinical risk prediction for pre-eclampsia in nulliparous women: development of model in international prospective cohort. BMJ. 2011;342:d1875.

54. Murphy NM, McCarthy FP, Khashan AS, Myers JE, Simpson NAB, Kearney PM, et al. Compliance with National Institute of Health and Care Excellence risk-based screening for Gestational Diabetes Mellitus in nulliparous women. Eur J Obstet Gynecol Reprod Biol. 2016;199:60–5.

55. McCowan LME, Roberts CT, Dekker GA, Taylor RS, Chan EHY, Kenny LC, et al. Risk factors for small-for-gestational-age infants by customised birthweight centiles: data from an international prospective cohort study. BJOG. 2010;117:1599–607.

56. Thaddeus S, Maine D. Too far to walk: maternal mortality in context. Soc Sci Med. 1994;38:1091–110.

57. von Elm E, Altman DG, Egger M, Pocock SJ, Gøtzsche PC, Vandenbroucke JP. The Strengthening the Reporting of Observational Studies in Epidemiology (STROBE) statement: guidelines for reporting observational studies. PLoS Med. 2007;4:e296.

58. Huberty J, Ehlers DK, Kurka J, Ainsworth B, Buman M, Gangwisch J, et al. Feasibility of three wearable sensors for 24 hour monitoring in middle-aged women. BMC Womens Health. 2015;15:55.

59. Wright SP, Hall Brown TS, Collier SR, Sandberg K. How consumer physical activity monitors could transform human physiology research. Am J Physiol Regul Integr Comp Physiol. 2017;:ajpregu.00349.2016.

60. Herring SJ, Foster GD, Pien GW, Massa K, Nelson DB, Gehrman PR, et al. Do pregnant women accurately report sleep time? A comparison between self-reported and objective measures of sleep duration in pregnancy among a sample of urban mothers. Sleep Breath. 2013;17:1323–7.

61. Okun ML, Kline CE, Roberts JM, Wettlaufer B, Glover K, Hall M. Prevalence of sleep deficiency in early gestation and its associations with stress and depressive symptoms. J women's Heal. 2013;22:1028–37.

62. Herring SJ, Nelson DB, Pien GW, Homko C, Goetzl LM, Davey A, et al. Objectively measured sleep duration and hyperglycemia in pregnancy. Sleep Med.

For peer review only - http://bmjopen.bmj.com/site/about/guidelines.xhtml

2014;15:51–5.

For peer review only

For peer review only - http://bmjopen.bmj.com/site/about/guidelines.xhtml

Table 1. Participating centers in the Maternal Actigraphy Exploratory Study I (MAES-I)

• The Maternity of CAISM, University of Campinas, in Campinas, SP, Brazil;<br>○ Local Principal Investigator: Maria Laura Costa.
• Maternity of the Clinic Hospital, Federal University of RS, Porto Alegre, RS, Brazil;<br>○ Local Principal Investigator: Janete Vettorazzi.
• Maternity of the University Hospital, Jundiaí Medical School in Jundiaí, SP, Brazil;<br>○ Local Principal Investigator: Ricardo Porto Tedesco.
• Maternity of Clinic Hospital, Federal University of Pernambuco, in Recife, PE, Brazil;<br>○ Local Principal Investigator: Edilberto A Rocha Filho.
• MEAC – School Maternity of the Federal University of Ceará, in Fortaleza, CE, Brazil.<br>○ Local Principal Investigator: Francisco Edson de Lucena Feitosa.

For peer review only

For peer review only - http://bmjopen.bmj.com/site/about/guidelines.xhtml

Table 2. Inclusion and Exclusion Criteria of MAES – I

Inclusion Criteria

- Singleton pregnancy
- Nulliparous (who had never given birth before)
- Between 19+0 – 21+0 weeks of gestation

Exclusion Criteria

- Unsure LMP and unwilling to have to date the Ultrasound
- ≥ 3 Miscarriages
- Major Fetal Anomaly/Abnormal Karyotype*
- Essential Hypertension Treated Pre-pregnancy
- Mod-Severe Hypertension at booking (≥ 160/100 mmHg) or Chronic hypertension using antihypertensive medication
- Pre-pregnancy Diabetes
- Renal Disease
- Systemic Lupus Erythematosus
- Anti-phospholipid Syndrome
- Sickle Cell Disease
- HIV or Hep B or Hep C positive
- Any condition that limits practice of physical activity
- Major Uterine Anomaly
- Cervical Suture
- Knife cone biopsy
- Ruptured membranes
- Use of long-term steroids
- Use of Low-dose Aspirin
- Use of Calcium (> 1g/24h)
- Use of Eicosapentaenoic acid (fish oil) > 2,7g
- Use of Vit. C ≥ 1000mg & Vit. E ≥ 400 UI
- Use of Heparin/LMW Heparin
- Untreated Thyroid disease
- Use of antidepressant and/or anxiolytic agents

* All information regarding fetal anomalies will be properly recorded

For peer review only - http://bmjopen.bmj.com/site/about/guidelines.xhtml

Table 3. Measurement frequency and maximum return periods according to gestational age – MAES I

Gestational age	Measurement frequency	Maximum return period (weeks)
19 – 32 weeks	20Hz	4
33 – 36 weeks	30Hz	2
37 – 42 weeks	50Hz	1

For peer review only

For peer review only - http://bmjopen.bmj.com/site/about/guidelines.xhtml

Table 4. Performance of wrist and hip actigraphy methods according to different activities

	Hip			Wrist		
	**Sens**	**Spec**	**BA**	**Sens**	**Spec**	**BA**
Sitting	0.894	0.923	0.908	0.883	0.870	0.876
Vehicle	0.870	0.987	0.929	0.823	0.964	0.893
Walking/running	0.687	0.981	0.834	0.574	0.983	0.779
Standing	0.797	0.929	0.851	0.687	0.904	0.795
Average	0.812	0.955	0.881	0.742	0.930	0.836

BA: balanced accuracy; sens: sensitivity; spec: specificity

Adapted from Ellis K *et al*. Hip and Wrist Accelerometer Algorithms for Free-Living Behavior Classification. Med. Sci. Sports Exerc. 2016.

For peer review only

Table 5. Severe Neonatal Morbidity definition according to term/preterm status

**Preterm**	**Term**
Grade III and IV intraventricular haemorrhage	Grade II or III hypoxic ischemic encephalopathy
Chronic lung disease (home on O_2 or on O_2 at 36 weeks gestation	Ventilation>24 hours
Necrotizing enterocolitis	Neonatal intensive care admission >4 days
Retinopathy of prematurity, stage 3 or 4	Apgar score <4 at 5 mins
Sepsis (blood or CSF culture proven)	Cord arterial pH<7.0 and/or base excess>-15
Cystic peri-ventricular leukomalacia	Neonatal seizures

For peer review only

For peer review only - http://bmjopen.bmj.com/site/about/guidelines.xhtml

Figure legends:

Figure 1. Set points of MAES-I study

Figure 2. Estimated prevalence of preterm birth and preeclampsia according to gestational age (RED represents majority of cases) and period of evaluation of PA and sleep patterns (in GREY)

For peer review only

For peer review only - http://bmjopen.bmj.com/site/about/guidelines.xhtml

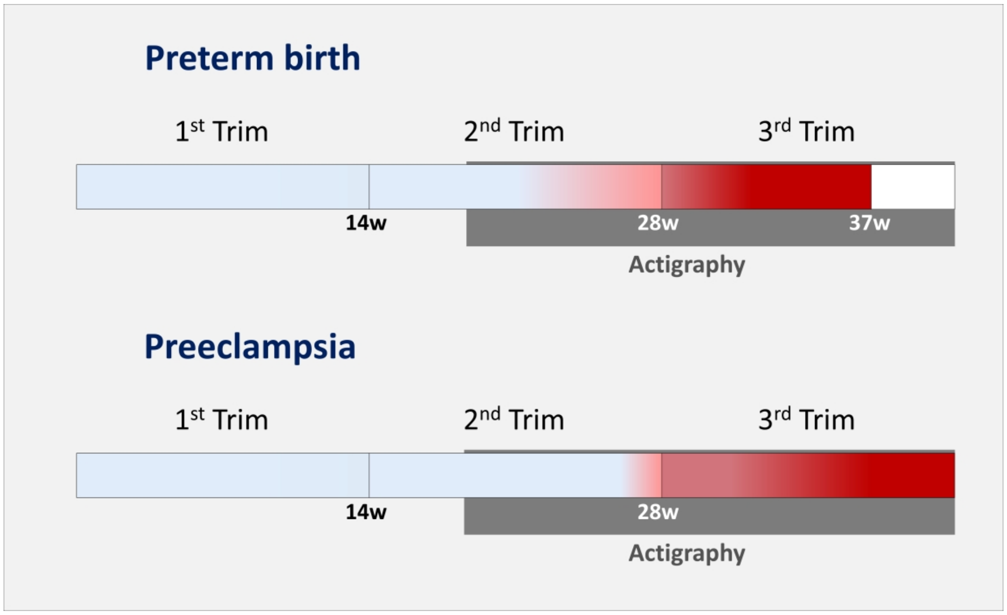

Figure 1

402x244mm (300 x 300 DPI)

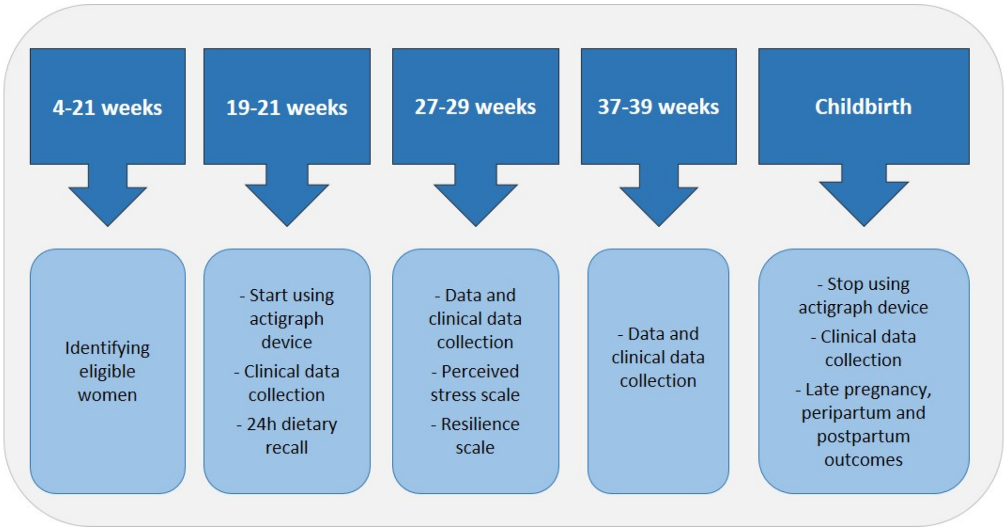

Figure 2

90x47mm (300 x 300 DPI)

STROBE 2007 (v4) checklist of items to be included in reports of observational studies in epidemiology*
Checklist for cohort, case-control, and cross-sectional studies (combined)

Section/Topic	Item #	Recommendation	Reported on page #
**Title and abstract**	1	(*a*) Indicate the study's design with a commonly used term in the title or the abstract	1,2
		(*b*) Provide in the abstract an informative and balanced summary of what was done and what was found	2
**Introduction**			
Background/rationale	2	Explain the scientific background and rationale for the investigation being reported	4-6
Objectives	3	State specific objectives, including any pre-specified hypotheses	6
**Methods**			
Study design	4	Present key elements of study design early in the paper	1,2,7
Setting	5	Describe the setting, locations, and relevant dates, including periods of recruitment, exposure, follow-up, and data collection	7
Participants	6	(*a*) *Case-control study*—Give the eligibility criteria, and the sources and methods of case ascertainment and control selection. Give the rationale for the choice of cases and controls<br>*Cross-sectional study*—Give the eligibility criteria, and the sources and methods of selection of participants	7,8
		(*b*) *Case-control study*—For matched studies, give matching criteria and the number of controls per case	
Variables	7	Clearly define all outcomes, exposures, predictors, potential confounders, and effect modifiers. Give diagnostic criteria, if applicable	11-15
Data sources/ measurement	8*	For each variable of interest, give sources of data and details of methods of assessment (measurement). Describe comparability of assessment methods if there is more than one group	11-15
Bias	9	Describe any efforts to address potential sources of bias	16,17
Study size	10	Explain how the study size was arrived at	15
Quantitative variables	11	Explain how quantitative variables were handled in the analyses. If applicable, describe which groupings were chosen and why	13
Statistical methods	12	(*a*) Describe all statistical methods, including those used to control for confounding	16,17
		(*b*) Describe any methods used to examine subgroups and interactions	16,17
		(*c*) Explain how missing data were addressed	17
		(*d*) *Case-control study*—If applicable, explain how matching of cases and controls was addressed<br>*Cross-sectional study*—If applicable, describe analytical methods taking account of sampling strategy	n/a
		(*e*) Describe any sensitivity analyses	n/a
**Results**			

For peer review only

Participants	13*	(a) Report numbers of individuals at each stage of study—eg numbers potentially eligible, examined for eligibility, confirmed eligible, included in the study, completing follow-up, and analysed	n/a
		(b) Give reasons for non-participation at each stage	n/a
		(c) Consider use of a flow diagram	n/a
Descriptive data	14*	(a) Give characteristics of study participants (eg demographic, clinical, social) and information on exposures and potential confounders	7,8
		(b) Indicate number of participants with missing data for each variable of interest	n/a
Outcome data	15*	*Case-control study*—Report numbers in each exposure category, or summary measures of exposure	n/a
		Cross-sectional study—Report numbers of outcome events or summary measures	n/a
Main results	16	(*a*) Give unadjusted estimates and, if applicable, confounder-adjusted estimates and their precision (eg, 95% confidence interval). Make clear which confounders were adjusted for and why they were included	n/a
		(*b*) Report category boundaries when continuous variables were categorized	n/a
		(*c*) If relevant, consider translating estimates of relative risk into absolute risk for a meaningful time period	
Other analyses	17	Report other analyses done—eg analyses of subgroups and interactions, and sensitivity analyses	17
**Discussion**			
Key results	18	Summarise key results with reference to study objectives	n/a
Limitations	19	Discuss limitations of the study, taking into account sources of potential bias or imprecision. Discuss both direction and magnitude of any potential bias	21
Interpretation	20	Give a cautious overall interpretation of results considering objectives, limitations, multiplicity of analyses, results from similar studies, and other relevant evidence	19-21
Generalisability	21	Discuss the generalisability (external validity) of the study results	21
**Other information**			
Funding	22	Give the source of funding and the role of the funders for the present study and, if applicable, for the original study on which the present article is based	24

*Give information separately for cases and controls in case-control studies and, if applicable, for exposed and unexposed groups in cohort and cross-sectional studies.

Note: An Explanation and Elaboration article discusses each checklist item and gives methodological background and published examples of transparent reporting. The STROBE checklist is best used in conjunction with this article (freely available on the Web sites of PLoS Medicine at http://www.plosmedicine.org/, Annals of Internal Medicine at http://www.annals.org/, and Epidemiology at http://www.epidem.com/). Information on the STROBE Initiative is available at www.strobe-statement.org.

For peer review only

BMJ Open

The identification of earlier predictors of pregnancy complications through wearable technologies in a Brazilian multicenter cohort: Maternal Actigraphy Exploratory Study I (MAES-I) study protocol

Journal:	*BMJ Open*
Manuscript ID	bmjopen-2018-023101.R2
Article Type:	Protocol
Date Submitted by the Author:	23-Dec-2018

Complete List of Authors:	Souza, Renato; Universidade Estadual de Campinas, Obstetrics and Gynecology<br>Cecatti, Jose; University of Campinas, Obstetrics and Gynecology<br>Mayrink, Jussara; Universidade Estadual de Campinas Faculdade de Ciencias Medicas<br>Galvão, Rafael; Universidade Estadual de Campinas, Obstetrics and Gynecology<br>Costa, Maria Laura; Universidade Estadual de Campinas, Obstetrics and Gynecology<br>Feitosa, Francisco; Universidade Federal do Ceara, Maternidade Escola<br>Rocha Filho, Edilberto; Universidade Federal de Pernambuco, Obstetrics and Gynecology<br>Leite, Debora; Universidade Federal de Pernambuco, Obstetrics and Gynecology<br>Vettorazzi, Janete; Universidade Federal do Rio Grande do Sul, Obstetrics and Gynecology<br>Tedesco, Ricardo; School of Medicine of Jundiai, Obstetrics and Gynecology<br>Santana, Danielly; Universidade Estadual de Campinas, Obstetrics and Gynecology<br>Souza, Joao Paulo; Universidade de Sao Paulo Faculdade de Medicina de Ribeirao Preto, Social Medicine

**Primary Subject Heading**:	Obstetrics and gynaecology
Secondary Subject Heading:	Obstetrics and gynaecology
Keywords:	wearable technologies, actigraph, physical activity, sleep patterns, pregnancy complications

SCHOLARONE™
Manuscripts

STUDY PROTOCOL

The identification of earlier predictors of pregnancy complications through wearable technologies in a Brazilian multicenter cohort: Maternal Actigraphy Exploratory Study I (MAES-I) study protocol

Renato T. Souza [1], Jose G. Cecatti [1#], Jussara Mayrink [1], Rafael B. Galvao [1], Maria L. Costa [1], Francisco E. Feitosa [2], Edilberto A Rocha Filho [3], Débora F Leite [1,3], Janete Vettorazzi [4], Ricardo P Tedesco [5], Danielly S Santana [1,5], Joao P. Souza [6], for the MAES-I Study Group*

Affiliations

[1] Department of Obstetrics and Gynecology, University of Campinas (UNICAMP) School of Medical Sciences, Campinas, SP, Brazil

[2] MEAC – School Maternity of the Federal University of Ceará, in Fortaleza, CE, Brazil

[3] Department of Maternal and Child Health, Maternity of Clinic Hospital, Federal University of Pernambuco, in Recife, PE, Brazil

[4] Department of Obstetrics and Gynecology, Maternity of the Clinic Hospital, Federal University of RS, Porto Alegre, RS, Brazil

[5] Department of Obstetrics and Gynecology, Jundiaí Medical School, Jundiaí, SP, Brazil

[6] Department of Social Medicine, Ribeirão Preto Medical School, University of Sao Paulo, Ribeirão Preto, SP, Brazil

*Membership of the MAES-I study group is provided in the Acknowledgments.

E-mail addresses: RT Souza: renatotsouzasp@gmail.com; JG Cecatti: cecatti@unicamp.br; J Mayrink: jussaramayrink@gmail.com; RB Galvão: rafaelbfg@gmail.com; ML Costa: mlaura@unicamp.br; FE Feitosa: edson.lucena@hotmail.com; EA Rocha Filho: edilbertorocha@globo.com; DF Leite: debora.leite@ufpe.br; J Vettorazzi: janetev@terra.com.br; RP Tedesco: rp.tedesco@yahoo.com.br; DS Santana: dany.fmj@terra.com.br; JP Souza: jp.souza@usp.br

#Corresponding Author

JG Cecatti

DO&G, University of Campinas

R. Alexander Fleming, 101

Campinas, SP, 13083-881

Brazil

E-mail: cecatti@unicamp.br

For peer review only - http://bmjopen.bmj.com/site/about/guidelines.xhtml

Abstract

Introduction: Non-invasive tools capable of identifying predictors of maternal complications would be a step forward in the improvement of maternal and perinatal health. There is association between modification of physical activity (PA) and sleep-wake patterns and the occurrence of inflammatory, metabolic, pathologic conditions as chronic diseases. The actigraph device is validated to estimate PA and sleep-wake patterns among pregnant women. In order to extend the window of opportunity to prevent, diagnose and treat specific maternal conditions, would it be possible to use actigraphic data to identify risk factors for the development of adverse maternal outcomes during pregnancy?

Methods and analysis: A cohort will be held in 5 centres from the Brazilian Network for Studies on Reproductive and Perinatal Health. MAES-I will enroll 400 low-risk nulliparous women that will wear the actigraph device on the wrist during day and night (24h/day) uninterruptedly from 19-21 weeks until childbirth. Changes in PA and sleep-wake patterns through pregnancy will be analyzed, considering gestational age ranges, in women with and without maternal complications during pregnancy, such as preeclampsia, preterm birth (spontaneous or provider-initiated), gestational diabetes, maternal haemorrhage and also perinatal outcomes. A predictive model for screening pregnant women at risk of presenting specific adverse maternal and perinatal outcomes is planned to be developed using the actigraphy data.

Ethics and Dissemination: MAES-I study has been reviewed and approved by each Institutional Review Board (IRB) and also by the National Council for Ethics in Research. Detailed information of the study is provided in the Brazilian Cohort website (www.medscinet.com/samba) and findings will be publicized in scientific literature and Institutional webpages.

Keywords: wearable technologies; actigraph; physical activity; sleep patterns; sleep-wake cycle; prediction; pregnancy complications.

For peer review only

Strengths and limitations of this study

- This multicentre cohort will collect comprehensive data on the main maternal and perinatal complications as pre-eclampsia, small for gestational age/fetal growth restriction, preterm birth and gestational diabetes mellitus.
- Physical activity and sleep patterns will be estimated through an innovative wearable device used in the natural environment of the study subject.
- Physical activity and sleep patterns will be estimated from the beginning of the second half of pregnancy until delivery, covering a wide interval during pregnancy and enabling the study of PA and sleep patterns changes throughout pregnancy.
- One possible limitation refers to the uncovered first half of pregnancy regarding this information.

For peer review only

For peer review only - http://bmjopen.bmj.com/site/about/guidelines.xhtml

Background

Reducing the global maternal mortality ratio to less than 70 per 100,000 live births by 2030 is one of the targets of the new United Nations Sustainable Development Goals [1]. Multiple challenges need to be tackled to achieve this target, but the 2016-2030 health and development agenda goes well beyond mortality reduction. The Global Strategy for Women's, Children's and Adolescent's Health aims to ensure that every newborn, women and child not only survive but thrive. This will only be possible if a transformative agenda, with innovation at the central stage, is put into action [2].

One of the major challenges that need to be addressed is optimizing the recognition of earlier predictors and identifiers of maternal and perinatal complications. Delays in diagnosing and managing maternal complications have been associated with poor outcomes [3]. The reduced self-perception of clinical signs related to maternal complications, difficulties in accessing the health system and poor quality of care may contribute to the late identification of complications and worsened prognosis. The development of a non-invasive Antenatal Care (ANC) tool capable of identifying maternal sub-clinical signs during pregnancy may provide the window of opportunity for the earlier identification of abnormal patterns of physiologic parameters related to pregnancy complications. We consider earlier identification when the recognition could be made before clinical presentation, when standard criteria based on clinical signs, symptoms, and supplementary tests are presented. Shortening the time between the onset of a complication and the initiation of the appropriate management allow for secondary prevention and reduction of maternal morbidity and mortality [3–7].

Pervasive computing (i.e. the trend towards embedding microprocessors in everyday-life objects so they can generate data) and wearable technology (i.e. clothing and accessories incorporating computer and advanced electronic technologies such as wrist and/or waistband sensors) are ubiquitous and able to generate a new dataset that needs to be correlated with pregnancy outcomes. Preterm birth and preeclampsia, for instance, are two important pregnancy complications that have a relatively long subclinical phase before the appearance of signs or symptoms [8, 9]. It is plausible that during this subclinical phase of certain conditions the pattern of physical activity (PA) or sleep-wake rhythm is affected in some way and this change could be captured through

For peer review only - http://bmjopen.bmj.com/site/about/guidelines.xhtml

wearable devices. Although some studies show that PA patterns (actigraphy parameters) may be related to systemic inflammation and diseases in the general population [10, 11], published literature correlating wearable technology data and maternal complications are very scarce.

The human circadian rhythm is ruled by endogenous physiologic mechanisms and environmental stimuli [12]. There is solid evidence showing that modification of circadian rhythm or sleep and PA patterns are an underlying condition related to inflammatory, degenerative and/or metabolic chronic diseases as diabetes, hypertension, and cancer [13]. Circadian misalignment is defined as having inappropriate timed sleep and wake, misplaced feeding periods and modification of activity behavior.

The determination of cause or consequence effect between these modifications and the development of pathological conditions is a complex task. It seems that changes in appetite-stimulating hormones, glucose metabolism, inflammatory markers, and mood are some of the related pathways [13–15]. Leproult *et al.* evaluated the effect of circadian misalignment on metabolic and inflammation markers in cardiovascular disease [15]. The insulin action and release, and also the levels of some inflammatory markers that are predictors for cardiovascular diseases, were abnormal in individuals with circadian misalignment. The mechanisms involved in the association between changes of PA pattern and pathologic conditions seem to be of multiple etiologies. Sani *et al.* assessed the circadian rithm of more than 2,300 African descendant adults. More than evaluating physical activity itself, the authors aimed to identify chronobiologic patterns of adults from different socioeconomic settings. The study identified that chronobiologic behavior can vary depending on individual BMI, socioeconomic background, work type and time of sunlight exposure. Possibly, many other factors are involved in modifications of chronobiologic behavior, such as pathologic conditions. Some metabolic, cognitive, cardiovascular and other chronic degenerative diseases have been associated with particular patterns of PA and sleep [10, 11, 16–18]. A previous observational study assessed various sleep parameters during pregnancy, e.g. sleep onset latency (SOL), wake after sleep onset (WASO) and total nocturnal sleep time (TST). Difficulty in initiating sleep in early pregnancy was associated with higher body mass

For peer review only - http://bmjopen.bmj.com/site/about/guidelines.xhtml

index, greater weight gain and higher blood pressure during pregnancy [17]. Palagini *et al.* reviewed clinical evidence between chronic sleep loss and pregnancy adverse outcomes, discussing common mechanisms of stress system activation [19]. Low-quality evidence suggests an association between sleep loss and prenatal depression, gestational diabetes, preeclampsia, abnormal length of labour, caesarean delivery, abnormal fetal growth, and preterm birth. Those results corroborate with other findings regarding pregnancy and sleep disorders [20–23].

The assessment of PA and the sleep patterns can be performed using small wrist (or waist) devices similar to a regular watch (actigraphy technology). The type of sensor, batteries, materials and output data have been substantially developed in recent years, enabling low cost, comfort, discretion and performance [24]. Nowadays there are devices that are portable, lightweight and with a large capacity to storage information, including a software with automatic scoring algorithms packages for the detection of wakefulness, sleep periods and PA [24, 25]. The actigraphy estimation of PA and sleep patterns is validated as a proxy for chronobiologic behavior [26–29] and 7 to 14 days using the actigraph device provides reliable estimates of PA behavior in older adults [30–32]. Both hip and wrist devices show reliable and acceptable performance in estimating PA and sleep-wake patterns [33–36].

The main advantages of using wearable devices for actigraphy is the non-invasiveness, 24/7 monitoring of the circadian pattern and PA, and informing sleep habits and parameters in the user natural environment [24, 25, 28]. We propose an innovative and strategic approach to monitor PA and sleep-wake patterns during pregnancy, establishing a large database comprised of clinical, epidemiological, PA and sleep-wake variables potentially capable of composing a prediction model for maternal complications during pregnancy. The main goal of this study is to identify earlier predictors of pregnancy complications by correlating data generated on PA and sleep patterns through wearable devices (wristband sensors) with maternal and perinatal complications and outcomes.

Methods/Design

Study design

For peer review only - http://bmjopen.bmj.com/site/about/guidelines.xhtml

We will conduct a cohort study of 400 pregnant women using wrist sensor bands able to capture information on daily physical activity and sleep patterns (exposure). This cohort study will be implemented in 5 ANC clinics linked to obstetric units in 3 different regions of Brazil that are already part of the Brazilian Network for Studies on Reproductive and Perinatal Health [37], as shown in Table 1. During a period of eight months, the ANC clinics will identify eligible cases for using the wristband sensors. Wearable technology data will be correlated with the occurrence of pregnancy and childbirth complications and outcomes, such as hypertensive disorders, gestational diabetes mellitus, fetal growth restriction and prematurity.

Eligible women will be identified up to 21 weeks of gestation and invited to participate. A proper consent form will be applied and the women who agree to participate will receive a wristband sensor to be used starting at 19-21 weeks until childbirth, uninterruptedly.

Study setting and population

Brazil is a multi-ethnic mixed-race population of diverse resourced settings [38]. Despite the considered high global overall human development index (HDI 0.727) in 2010, the HDI of Brazilian municipalities ranged from 0,862 to 0,418 [39]. The possibility of considering such mixed population is suitable to explore information regarding maternal patterns of mobility and sleep, maximizing external validity and comparisons to other populations. The following reasons support the study population being focused in low-risk nulliparous women: 1) Previous obstetric history can refer to known risk factors for many maternal complications such as preterm birth, preeclampsia, and diabetes [13, 40]. Therefore nulliparous women enable unbiased sampling regarding obstetric history. 2) Women with previous morbidity such as hypertension, diabetes, nephropathy or others chronic/degenerative diseases are more likely to present abnormalities of sleep-wake rhythm or physical activity patterns during pregnancy.

Sampling

The five participating centers are regional referral obstetric units responsible for antenatal care assistance mainly for high-risk pregnant women. Participating centers are listed in Table 1. Nevertheless, there are primary health care units strategically linked

For peer review only - http://bmjopen.bmj.com/site/about/guidelines.xhtml

with these participating centers, enabling the identification and enrollment of women with non-pathological pregnancies. The recruitment strategies include approaching all eligible women in these participating centers and their linked facilities. An informed consent form will be applied for women who agree to participate.

Eligible women: Low-risk pregnant subjects

There is no international consensus on the criteria for low-risk pregnancies, although there are several known factors associated with maternal and perinatal adverse outcomes. A recent study evaluating complications of "low-risk" pregnancies of US Americans (10 million births from 2011 through 2013) showed that 29% of low-risk women had an unexpected complication requiring no routine obstetric/neonatal care [41]. This shows the difficulty in establishing a "low-risk profile" for maternal/perinatal complications. In order to better identify eligible low-risk pregnant women, we excluded potential known confounders of pre-pregnancy conditions that could be related to adverse maternal or perinatal outcomes as shown in Table 2, so we could assess PA and sleep patterns of a mostly "normal" population. Nonetheless, lifestyle habits and body composition (Body mass index, height, etc.) characteristics, and some non-severe chronic diseases as non-severe anaemia and/or asthma are not among the exclusion criteria in this study but may be part of subgroup analyses (composition of any previous disorder, e.g.). Intra and inter-individual analyses of PA and sleep patterns enable the identification of potential confounders affecting primary outcomes, avoiding potential biases. It means that comparison of PA and sleep pattern parameters collected in different stages of pregnancy from the same participant (intra-individual analysis) and collected at the same stage of pregnancy from different participants (inter-individual analysis) will be carried out.

Eligible women will be enrolled between 04-21 weeks. The inclusion and exclusion criteria are presented in Table 2.

Data collection methods

Essentially, MAES I study is comprised of 4 key set points – 3 clinical visits during pregnancy and a postnatal visit. The clinical visits will be held at 1) between 19-21 weeks; 2) between 27-29 weeks; 3) between 37-39 weeks. At first, second, third and postnatal

For peer review only - http://bmjopen.bmj.com/site/about/guidelines.xhtml

visits, additional information regarding maternal history, details on pregnancy complications, maternal biophysical data (weight, height, skinfolds) and pregnancy adverse outcomes will be collected following a specific Standard Operating Procedure (SOP) specially developed for MAES-I study. Additionally, the Perceived Stress Scale [42] and Resilience Scale [43] will be applied during 27-29w visit. Figure 1 shows the set points of MAES-I study.

Eligible women will be invited to use a 43mmx40mmx13mm water-resistant wrist device similar to a regular watch (GENEActiv Original – Activinsights®). The device contains accelerometer to estimate PA and sensors to estimate sleep-wake patterns through light and temperature measurements, using a proper software algorithm.

At the first set point of MAES-I study (between 19+0 - 21+0 weeks of gestation), eligible women who agreed to participate will be instructed that the wrist bracelet device should be worn on the non-dominant arm during day and night (24h/day) uninterruptedly until childbirth (including bathing or aquatic activities). The participant will not have to press any button or take any special care regarding the functioning of the device, which will be configured to register physical activity and sleep-wake data automatically from the moment it is delivered at the antenatal care visit. Also the battery charge will be held by the research assistant before delivering the device to the participant woman.

The acquisition of actigraphy data can be performed in different frequencies (from 10Hz to 100Hz). Since the frequency of data acquisition impacts on the battery life of the device (inverse relationship), the measurement frequency will be set up according to the participant´s gestational age (Table 3). This information will be registered in the database accordingly. The data accumulated will be downloaded during participant´s antenatal care visits, according to the maximum return periods showed in Table 3. The maximum return periods were calculated taking into consideration the expected battery life. At each antenatal care visit, the used device will be returned to the research team and a new charged device will be provided to the participant.

A leaflet with detailed information and FAQ (Frequently asked questions) on the device will also be provided to the women. They will also have a cell phone number to call whether doubts arise regarding the procedures for using the device, or if any technical or medical concern arises.

For peer review only - http://bmjopen.bmj.com/site/about/guidelines.xhtml

During each antenatal care visit, the wrist device will be connected to a charge base which can be connected to a computer through an USB connection. All actigraphy data will be extracted to the computer as a raw data ".bin" file. An open source proper software (Geneactiv Software®) will allow to convert this file into ".csv" compressed epoch files for each 30 minutes of registered data, which can be read in Excel® program. Then, the actigraphy data will be uploaded to an online database platform developed by MedSciNet®, where all clinical data of the study will also be registered.

The actigraphy software uses several algorithms to translate numerical information obtained though the epoch files into physical activity and sleep-wake patterns, which will compose the independent variables of this study. The database in centralized, secure, internet-based and allows several procedures for prospective and retrospective monitoring, hierarchical access (local user, general manager, etc.). The database will be translated into Portuguese and English, facilitating data collection for Portuguese-speaking team and international monitoring. A correspondent paper form will be available for data collection if necessary (e.g. internet connection failure for instance).

Decision to start monitoring PA and sleep patterns between 19-21weeks

There are various underlying mechanisms involved in the development of the maternal and perinatal adverse outcomes that will be assessed, as preterm birth, preeclampsia, gestational diabetes, fetal growth restriction and small for gestational age. The pre-clinical phase, stage where there are no clinical signs or symptoms, might be different for each disease and dependent on environmental and individual aspects. The study of adverse maternal and perinatal predictors has been focused in early pregnancy so far (first trimester, <14 weeks of gestation), aiming to maximize the window of opportunity for preventative interventions. However, we hypothesized that the modification of PA and/or sleep pattern due to maternal underlying changes of biological function might not be evident at a very early stage in pregnancy before the beginning of the pre-clinical phase. Our hypothesis is that it possibly occurs shortly before symptoms.

Additionally, we took into account that the occurrence of the main maternal complications, as preeclampsia, fetal growth restriction, and preterm birth, are more common in late pregnancy to establish the period between 19-21 weeks as appropriate to start the assessment of PA and sleep patterns. A recent cross-sectional study

For peer review only - http://bmjopen.bmj.com/site/about/guidelines.xhtml

conducted in 20 referral centres in Brazil, including the five participating centres of this proposal, showed that the occurrence of preterm birth before 28 weeks comprised less than 1% of all births and less than 8% of all preterm births [44]. In addition, the early onset of preeclampsia (before 34 weeks of gestation) complicates less than 0.4% of all pregnancies, according to a large retrospective cohort of more than 450,000 deliveries in USA [45]. Figure 2 outlines the predicted prevalence of preterm birth and preeclampsia in the second trimester, highlighting its clinical presentation, when classic symptoms and signs of a certain disease/complication are presented, through pregnancy in red. Our hypothesis is that PA and sleep patterns might be altered closely to the clinical presentation, still in preclinical phase when there is no symptoms or signs.

In brief, as an exploratory study, we indeed needed to make an arbitrary decision regarding interval of monitoring PA and sleep patterns. For that, we had taken into consideration: 1) the main maternal/perinatal complications of interest occur in the second half of pregnancy, more precisely in late pregnancy (Figure 2); 2) we hypothesize that any potential change on PA or sleep patterns might occur days or weeks before the onset of maternal or perinatal complication. Then, we focused monitoring women during second half of pregnancy.

Thus, the start of assessment between 19-21 weeks seems to be very reasonable, providing a wide interval to monitor and predict the main maternal and perinatal adverse outcomes.

Actigraph device

The actigraph device that will be used to monitor PA and sleep-wake patterns is GENEActiv Original (GENEActiv, Activinsights Ltd, Huntingdon, UK). The device has multiple sensors as microelectromechanical (MEMS) accelerometer, temperature (linear active thermistor) and light (silicon photodiode), providing crude raw data for a variety of applications.

Wrist vs waist wear: advantages and performance

Wrist wear of actigraph devices provides more comfortable use during wake and sleep periods and highest wear time compared to waist monitors [33, 46]. A non-systematic review published in 2011 showed that actigraphy is a useful and reliable tool to assess

For peer review only - http://bmjopen.bmj.com/site/about/guidelines.xhtml

sleep patterns and circadian rhythm disorders, although there are some limitations on diagnosing sleep diseases or measuring sleep stages [25]. Actigraphy showed excellent concordance with polysomnography in assessing sleep parameters in healthy subjects (sensitivity >90% in estimating total sleep time, for instance). A recent study evaluated the concordance of physical activity estimation of wrist device in free-living settings in forty overweight or obese women [34]. They used both wrist and hip devices, and a small camera that captured participant behaviour for 7 days, enabling the monitoring of physical activity behaviour (gold-standard comparison). The hip and wrist machine learning (ML) classifiers used are different due to the different methods/algorithms to estimate physical activity [34]. The sensitivity and specificity of hip and wrist estimations according to Ellis *et al* are showed in Table 4 [34].

Two years ago, the same author had published a similar evaluation using 40 adults (women and men), showing that the hip and wrist accelerometers obtained an average accuracy of 92.3% and 87.5%, respectively, in predicting PA types [47].

Staudenmayer *et al* developed an investigation with 20 participants also using two devices (wrist- and hip- worn), and showed that wrist actigraphy can estimate energy expenditure accurately and relatively precisely [48]. Another study evaluating PA patterns in a free-living environment with wrist devices showed that women in the top 40% or bottom 40% of the distribution of daily PA, hip and wrist accelerometers agreed on the classification for about 75% of the women [49]. Additionally, the total activity (counts per day) was moderately correlated (Spearman's r = 0.73) between the wrist and hip worn devices.

At the best of our knowledge, there are no systematic reviews or other high-quality evidence-based recommendation supporting a particular method. Although wrist wear of actigraphy is not the more traditional method, it might be the best choice for assessing long periods of PA or sleep patterns, even more considering the similar performance of the waist wear. The current proposal does not intend to diagnose pathologic behaviours or diseases, but to identify different patterns along pregnancy and in different subgroups of women. Therefore, supported by the evidence that wrist wear of actigraphy devices can accurately and more comfortably estimate PA and sleep

For peer review only - http://bmjopen.bmj.com/site/about/guidelines.xhtml

patterns, mainly for long periods and in the free-living environment, the MAES-I study group addopted wrist wear devices.

Main variables

The independent variables assessed as potential predictors of maternal complications will be related to sleep-wake cycle and mobility as:

"Sleep" variables

- Sleep onset latency (SOL): time elapsed between full wakefulness to sleep.
- Total sleep time (TST): The amount of actual sleep time in a sleep episode (excludes awakes).
- Wake after sleep onset (WASO): defined as total amount of time awake after sleep.
- Sleep Efficiency (SE): the proportion of sleep in the period potentially filled by sleep-ratio of total sleep time to time in bed.

The actigraph device collects many pieces of information related to body position and body movements to estimate the described sleep variables. Then, actigraphy software will be used to analyse the data and generate the output variables.

"Physical activity" variables

Actigraphy technology estimates physical activity through various parameters collected by the actigraph device. Briefly, according to Freedson *et al*, the triaxial sensors stressed by acceleration forces can estimate the intensity of movements. The acceleration signal is converted to digital signal and summed over a user specified time interval (epoch). At the end of each epoch the activity count is stored. Then, according to Count per minute (CPM) cut points, the PA intensity can be categorized [50]. The information is translated by the software using proper algorithms into quantitative variables as following:

- Sedentary time (hours/day): the number of hours per day of count per minute between 0-99.

- Light activity (hours/day): the number of hours per day having count per minute between 100 - 1951.

For peer review only - http://bmjopen.bmj.com/site/about/guidelines.xhtml

- Moderate activity (minutes/day): the number of hours per day having count per minute between 1952 - 5724.
- Vigorous activity (minutes/day): the number of hours per day having count per minute between 5725 - 9498.
- Very vigorous activity (minutes/day): the number of hours per day having count per minute between 9499 - ∞.
- MET rates: Metabolic Equivalents (METs) are commonly used to also express the intensity of physical activities. One MET is the energy cost of resting quietly, often defined in terms of oxygen uptake as 3.5 mL·kg^{-1}·min^{-}1. MET rate expresses a person's working metabolic rate relative to their resting metabolic rate. Briefly, the triaxial piezoelectric sensors stressed by acceleration forces can estimate the intensity of movements, converted to the oxygen consumption required to perform such movement.
- Step counts/day: estimated steps count per day (estimated by proper algorithms using accelerometer data.)

Outcomes

The primary outcomes are late pregnancy complications as:

- Preeclampsia: Hypertension after 20 weeks of gestation, systolic BP ≥ 140mmHg and/or diastolic BP ≥ 90mmHg (Korotkoff V) on at least 2 occasions 4h apart with: 1) Proteinuria 300 mg/24h or spot urine protein: creatinine ratio 30 mg/mmol creatinine or urine dipstick protein ≥ (+) OR, in the absence of proteinuria, hypertension and 2) any multi-system complication that are: Haematological abnormalities; thrombocytopenia (platelets < 100 x 10^9/L); Disseminated intravascular coagulation (DIC), and haemolysis; Liver disease: increased aspartate transaminase and/or alanine transaminase > 45 IU/L and/or severe right upper quadrant or epigastric pain, liver rupture; Neurological problems: eclampsia, imminent eclampsia (severe headache with hyperreflexia and persistent visual disturbance), cerebral haemorrhage; Acute renal insufficiency: new increase in serum creatinine to > 100 mmol/L antepartum or > 130 mmol/L postpartum; Pulmonary oedema confirmed by chest x-ray [51].

For peer review only - http://bmjopen.bmj.com/site/about/guidelines.xhtml

- Gestational Diabetes: new diabetes developing in pregnancy according to the WHO recommendation [52] that defines gestational diabetes as having:
    - Fasting plasma glucose ≥ 92 mg/dl, or
    - 1-h plasma glucose tolerance test (75g load) ≥ 180 mg/dl, or
    - 2-h plasma glucose tolerance test (75g load) ≥ 153 mg/dl.
- Spontaneous Preterm Birth: spontaneous onset of preterm labour or premature rupture of membranes leading to preterm birth, childbirth before 37 weeks of gestation.
- Provider-Initiated Preterm Birth: defined as childbirth occurring at less than 37 weeks, medically indicated due to maternal/fetal compromise or both;
- Maternal Hemorrhage: Classified in 1) Antepartum haemorrhage defined as bleeding from the genital tract after 24 weeks of gestation; 2) Primary Postpartum haemorrhage as the loss of 500 ml blood or more from the genital tract within 24 hours of the childbirth.

Secondary outcomes include childbirth variables and neonatal adverse outcomes as fetal death, caesarean section, small for gestational age (defined as birth weight below percentile 10 for gestational age), Apgar score < 7 at 5 minutes, neonatal severe morbidity (Table 5) and neonatal mortality before discharge.

Plans for analyses

Sample size estimation

This is an exploratory and innovative study focused on a specific population (pregnant women) and therefore there are no previously published parameters available for sample size estimation. Considering a relatively wide range of frequency of complications arising in pregnancy from 3 to 20% (including preeclampsia, fetal growth restriction, gestational diabetes, hemorrhage, preterm birth, etc.), a theoretical large population size (above 1 million pregnant women), an acceptable margin of error of 4%, the involvement of 5 clusters (participating centers) and a 95% level of confidence, 384 women would be necessary. Therefore, we are rounding up this estimation for at least 400 initially low-risk pregnant women to be enrolled in the study. We estimated the incidence of some main maternal complications considering the following studies:

For peer review only - http://bmjopen.bmj.com/site/about/guidelines.xhtml

- Pre-eclampsia: An international prospective cohort study with nulliparous women called SCOPE used similar criteria for the low-risk profile, having 5% of incidence of pre-eclampsia [53].
- Preterm Birth: A recent cross-sectional study conducted in 20 referral obstetric centres in Brazil, including the five participant centres of this proposal, showed a prevalence of 12.3% of all births [44].
- Gestational Diabetes: SCOPE international cohort, previously mentioned, had a prevalence of 8.9% of gestational diabetes in low-risk screened nulliparous women, according to mainly to the NICE guidelines [54].
- Fetal growth restriction/small for gestational age: SCOPE international cohort, previously mentioned, had a prevalence of 10.7% of newborns small for gestational age, according to the customized centiles of birthweight (<10%)[55].

Statistical Analysis details

According to these studies above, the predicted incidence of these complications seems reasonable and reproducible in our cohort. Then, sample size estimation might assure enough cases of maternal and perinatal complications for the current proposal.

The epoch files obtained from Geneactv Software by reading data of sleep variables and physical activity parameters will be translated into numerical results and then averaged in periods of 7 days. By doing this, there will be one value to be used in statistical analysis for each variable per week of use of the wrist device.

Firstly, we will identify PA and sleep-wake patterns of women who did not develop adverse maternal or perinatal outcomes. It will permit the recognition of a normal PA and sleep-wake patterns in low-risk population without complication during pregnancy. Using the same population, we will analyze changes in PA and sleep-wake patterns through pregnancy, allowing for gestational age periods.

Then, we will compare the PA and sleep-wake patterns of women who developed specific adverse maternal or perinatal outcomes with those who did not. The differences between groups might be identified to be used as potential markers for specific pregnancy complications.

For peer review only

For peer review only - http://bmjopen.bmj.com/site/about/guidelines.xhtml

After that, we will analyze changes in PA and sleep-wake patterns of women who developed adverse maternal or perinatal outcomes through pregnancy, comparing the patterns and trying to discover which changes and when before the onset it would be related to pregnancy complications. If possible, we will conduct subgroup analysis including subpopulation with potential higher risk for maternal complications (confounder variables), including obesity, smoking, etc.

Finally, we will develop a predictive model for screening pregnant women for risk of specific adverse maternal and perinatal outcomes using PA and sleep-wake data estimated with actigraphy technology.

The analysis will be performed using the actigraph software that translates the collected information into PA and sleep-wake parameters. Additionally, data regarding time of sleep onset latency, wake after sleep onset and total sleep time as well as sleep efficiency will be compared between participants along the whole pregnancy time using Friedman and Wilcoxon for paired samples. ANOVA and t-test will be used to compare the sleep parameters between the participants for each week of gestational age for repeated measures. The same tests will be applied to analyze quantitative data regarding the median of number of hours per day having different types of physical activity (sedentary, light, moderate, vigorous and very vigorous), MET rates and estimative os steps/day through the entire gestational period examined, and the comparison between the participants for each week of gestational age. Also, we will address sensitivity, specificity and likelihood ratio for altered PA and sleep patterns or for their changes throughout pregnancy.

Discontinuation of participants

The criteria for discontinuation include:

- Withdrawal of consent;
- Not regularly using the actigraph device for long periods, less than 50% of all planned time. The information that they are not using the device properly will be recorded if women notice the MAES-I team. Otherwise, the low use of the device will be noticed after data discharge during antenatal care visits.
- The loss to follow-up, not allowing the download of actigraphy data.

For peer review only - http://bmjopen.bmj.com/site/about/guidelines.xhtml

Those women who decide to leave follow-up will be asked by telephone call to return the wrist device and a last visit will be set in order to regain the wrist monitor and direct the woman to a proper antenatal care service to continue their consultations.

Data and Sample Quality

All entered data will be prospectively and retrospectively monitored by local research assistants and a global monitor. Internal consistency of variables will be constantly performed by the database and error messages are automatically flagged. A local research assistant will be responsible for checking all forms and actigraphy data before locking forms, assuring good quality of data (double-checking entered data and checking for inconsistencies between variables, for instance). Then, the local principal investigator (PI) will be responsible for signing the case, enabling its incorporation to the final database. The University of Campinas will coordinate, implement and monitor the study in the five participating centres. A general manager and a global monitor are also part of the team of the coordinating centre. The local team of each participating centre is comprised of a Local PI and research assistants.

Ethics and Dissemination

MAES-I study focuses on low-risk nulliparous Brazilian pregnant women. Despite being considered of low risk for maternal and perinatal complications, they are not free of suffering complications. Furthermore, the first and second delays, defined as a delay in deciding to seek care and delay in reaching a health care facility [56], are not uncommon, establishing a barrier between earlier recognition of symptoms and timely interventions capable to successfully treat potentially life-threatening conditions. We believe that women will feel encouraged, empowered and willing to participate in the study that aims to develop a potentially useful prenatal care tool to identify the risk for maternal and perinatal morbidity and mortality. Following national ethical regulations, the participants will not receive any financial compensation.

Women who agree to participate in the study will not have any disadvantages or compromise of their prenatal care. On the contrary, they will receive a telephone number to contact the clinical researchers at any time (24/7 service), which enables a closer contact with researchers and providers of care, since the MAES-I team are

For peer review only - http://bmjopen.bmj.com/site/about/guidelines.xhtml

committed to contacting providers of care if any potential complication is noticed by participants.

The participating women will not be responsible if loss, theft or damage to the wrist device occurs. They will be asked only to use the device just as a regular wrist-watch would be worn and no self-damage is expected in those who use it.

Participating women will not be able to identify any PA or sleep parameters at any stage of the study. The download of the data is only possible through the own licensed software of the device. Actigraphy devices provided for participating women have a unique code which will be recorded in the database together with the interval of use for each women. Actigraphy data will be labelled using participant ID, device number, gestational age when starting using each device and return date of each device. The use of such codes, ID`s and numbers will ensure confidential identify for all participating women. The identity of all women will be kept confidential.

MAES-I study has been reviewed and approved by the National Committee for Ethics in Research of Brazil (CONEP) and by the Institutional Review Board (IRB) of the coordinating centre (Letter of approval 1.834.116 issued on 24th November 2016) and of all other Brazilian participating centres. All women who will be enrolled in the MAES-I cohort will sign an informed consent form.

The ethical principles stated in the Brazilian National Heath Council (Resolution CNS 466/12) will be respected in every stage of this study. The anonymity of the source of information will be guaranteed and the care for the women will be provided independently of her agreement to participate in the study. The study also complies with the Declaration of Helsinki amended in Hong Kong in 1989.The methodological and ethical aspects of MAES-I study protocol were developed following STROBE guidelines [57].

Patient and Public Involvement

Patients and public were not involved in this study for the development of the research question and outcome measures. However, the choice for a wrist device was based on the preference of users as reported. Participants of the study will have access to the results by its webpage that will be open access.

For peer review only - http://bmjopen.bmj.com/site/about/guidelines.xhtml

Detailed information of the study is provided in the Brazilian Cohort website (www.medscinet.com/samba) and findings will be publicized in scientific literature and Institutional webpages. We intend to disseminate our findings in scientific peer-reviewed journal, general free access website, specialists' conferences, and to our funding agencies.

Discussion

The actigraphy is an innovative, non-invasive, non-operator dependent, wearable technology, which enables the estimative under real life conditions of diverse variables related to mobility, physical activity, sleep-wake, and circadian cycle patterns. Actigraph devices show high sensitivity in sleep-wake parameters detection and are currently highly recommended by the American Sleep Disorder Association for diagnosis and therapy response of circadian rhythm disorders [27, 28, 58]. Although some studies show that 7 to 14 days using the actigraph device provides reliable estimates of physical activity behavior in older adult, it is not absolutely clear how many days is needed to estimate habitual PA by using the wrist/waist device during pregnancy. In general, it seems to depend mainly on the type of actigraph device, position of wear and target population [30, 33]. Nevertheless, MAES-I study will provide sufficient data to assess different patterns along pregnancy.

The use of wearable physical activity monitors has grown enormously due to the interest about the relationship between the pathophysiology of diseases and physical activity and sleep patterns. A recent study on the use of physical activity monitors in human physiology research unravels the current and potential uses of actigraph device as in strategies to promote healthier behaviour or to predict outcomes [59]. The authors conclude that physical activity monitors, as others new 21st century technologies, have already transformed physiology research, revolutionizing the way we assess patients and opening new areas of interest. In addition, the use of objectives measures to evaluate habitual sleep duration and outcomes in pregnancy is critical, taking into account recent investigations reporting little agreement between objective and subjective assessments of sleep time [60].

For peer review only - http://bmjopen.bmj.com/site/about/guidelines.xhtml

Alterations in sleep patterns, as less deep sleep and more nocturnal awakenings, can be observed in pregnancy as early as 10-12 weeks gestation [61]. Sleep disturbances during pregnancy have been associated with preterm delivery, gestational hypertensive disorders, glucose intolerance and increased risk of caesarean delivery [19]. Shorter night time sleep was also associated with hyperglycemia [62]. Persistent sleep deficiency is correlated with depressive symptoms and stress perception by pregnant women [61]. These studies lay correlation between PA patterns and sleep disturbances determining complications, in a well-established relationship of cause and consequence, although sometimes it could not be adequately determined due to the study design [17].

In a distinct way, our analysis intends to figure out if the maternal complication could be identified by physical activity and/or sleep patterns modifications, even during its pre-clinical period, previous the appearance of clinical signs. Considering the existing evidence, we speculate that the PA and/or sleep patterns change days or weeks before the clinical presentation of the complication. In general, the signs and symptoms of some maternal outcomes are part of the gold-standard criteria for diagnosis (high blood pressure, proteinuria and/or edema in the case of preeclampsia; premature contractions and cervical ripening/dilation in preterm birth; abnormal placental blood flow and insufficient fetal growth in Intrauterine Growth Restriction). We acknowledge the fact that there are potential confounders and limitations in predicting maternal and perinatal complications using PA and sleep patterns estimated by actigraph devices. We expect that our studied population will have different subgroups of women with different risks and associated factors playing a role on maternal complication. It includes obesity, smoking, extremes of age, for instance. None of those factors was considered exclusion criteria and, if possible, we intend to assess subgroup analysis for those maternal subgroups at they might present different PA and sleep patterns. Nonetheless, we decided to perform a pragmatic approach, not excluding such common factor from our sample.

The use of actigraph device during prenatal visits has a potential to become a new tool to monitor pregnant women, improving maternal health care, identifying altered PA and/or sleep patterns, measured objectively through actigraphy, before the occurrence of those signs and symptoms. Therefore, the focus would be offering new technology to

For peer review only - http://bmjopen.bmj.com/site/about/guidelines.xhtml

monitor the development of a potential maternal complication. Other positive points of our study are the period of data collection (from 19 weeks till delivery) and the low-risk profile of the cohort. Through which, it would be possible to describe a PA and sleep patterns in a low-risk pregnant population and better interpret actigraphy data among pregnant women. The current clinical and biological predictors for the main maternal complications as preeclampsia, preterm birth, maternal haemorrhage, and gestational diabetes still lack for effective sensitivity and specificity.

If this is confirmed to be true, an important step will be achieved for a possible introduction of screening non-invasive procedures during prenatal care with the purpose of identifying women at higher risk of developing those conditions. Therefore, they could receive specific orientation on prevention and earlier detection of the onset of condition for taking immediate action to look for professional health care and receiving appropriate interventions, avoiding delays that are the most striking factor for the low quality of care the women usually receive in low and middle-income settings, contributing to the still high burden of maternal morbidity and mortality. If we were successful in identifying such "specific patterns of physical activity and sleep" as predictors for pregnancy complications, further validation studies will necessarily be recommended for assessing its effectiveness for the whole management of such conditions. Additionally, MAES-I will enable further specific studies among high risk population and also will help to identify the best gestational age for monitoring, giving the means to target a specific gestational age interval.

For peer review only

1 **Abbreviations**

ANC – antenatal care

BMI – body mass index

BP – blood pressure

CPM – count per minute

dL – decilitre

FAQ – frequently asked questions

h – hour

HDI – human development index

Hz - hertz

Kg – kilogram

L – litre

MAES-I – maternal Actigraphy Exploratory Study I

MEMS – microelectromechanical

MET – metabolic equivalent

METs – metabolic equivalents

min – minutes

mg – milligram

mL – millilitre

mmol – millimole

NICE – National Institute for Health and Care Excellence

PA – physical activity

PI – Principal investigator

SE – sleep efficiency

SCOPE – SCreening Of Pregnancy Endpoints

SOL – sleep onset latency

TST – total nocturnal sleep time

US – United States

USA – United States of America

w – week

Competing interests

The authors declare that they have no competing interests.

Authors' contributions

All authors contributed to the overall study design and specific methodologies. RTS, JGC, JM, MLC and JPS conceived the study design and wrote de first version of the study protocol. In a first meeting, the protocol was discussed and incorporated suggestions from RBG, FEF, ERF, DFL, JV, RPT and DSS. RTS, JGC, JM, and RBG planned the implementation of the study and developed the necessary material. RTS, JM, MLC and JGC drafted the manuscript that was afterwards revised by all other authors who gave suggestions. All authors discussed and made important contributions to the manuscript, read and approved the final version for submission.

For peer review only

Acknowledgements

The MAES - I study group also included: Carina B Luiz and Luiza C Brust, School of Medicine, Federal University of Rio Grande do Sul, Porto Alegre, Brazil; Danilo Anacleto and Lívia C Nascimento, School of Medicine, Federal University of Pernambuco, Recife, Brazil; Daisy Lucena and Denise Ellen F Cordeiro, School of Medicine, Federal University of Ceará, Fortaleza, Brazil; Mariana B Rogerio, Departament of Obstetrics and Gynecology, Jundiaí Medical School, Jundiaí, Brazil.

Funding

This study was granted by The Bill and Melinda Gates Foundation through the Grand Challenge Exploration program, call 19 (research grant OPP1182749).

For peer review only

For peer review only - http://bmjopen.bmj.com/site/about/guidelines.xhtml

References

1. United Nations Development Programme (UNDP). Sustainable Development Goals. 2015. http://www.un.org/sustainabledevelopment/health/. Accessed 27 Apr 2016.

2. WHO, World Health Organization. Global Strategy. Global Strategy for Women's, Children's and Adolescents' Health, 2016 - 2030. 2015. http://www.who.int/life-course/partners/global-strategy/global-strategy-2016-2030/en/. Accessed 9 Jul 2016.

3. Pacagnella RC, Cecatti JG, Parpinelli MA, Sousa MH, Haddad SM, Costa ML, et al. Delays in receiving obstetric care and poor maternal outcomes: results from a national multicentre cross-sectional study. BMC Pregnancy Childbirth. 2014;14:159.

4. Haddad SM, Cecatti JG, Souza JP, Sousa MH, Parpinelli MA, Costa ML, et al. Applying the maternal near miss approach for the evaluation of quality of obstetric care: a worked example from a Multicenter Surveillance Study. Biomed Res Int. 2014;2014:989815.

5. Pacagnella RC, Cecatti JG, Osis MJ, Souza JP. The role of delays in severe maternal morbidity and mortality: expanding the conceptual framework. Reprod Health Matters. 2012;20:155–63.

6. Vogel JP, Souza JP, Mori R, Morisaki N, Lumbiganon P, Laopaiboon M, et al. Maternal complications and perinatal mortality: findings of the World Health Organization Multicountry Survey on Maternal and Newborn Health. BJOG. 2014;121 Suppl:76–88.

7. Souza JP, Gülmezoglu AM, Vogel J, Carroli G, Lumbiganon P, Qureshi Z, et al. Moving beyond essential interventions for reduction of maternal mortality (the WHO Multicountry Survey on Maternal and Newborn Health): a cross-sectional study. Lancet. 2013;381:1747–55.

8. Brosens I, Pijnenborg R, Vercruysse L, Romero R. The "Great Obstetrical Syndromes" are associated with disorders of deep placentation. Am J Obstet Gynecol. 2011;204:193–201.

9. Di Renzo GC. The great obstetrical syndromes. J Matern neonatal Med. 2009;22:633–5.

10. Loprinzi PD. The effects of objectively-measured, free-living daily ambulatory movement on mortality in a national sample of adults with diabetes. Physiol Behav. 2016;154:126–8.

11. Biswas A, Oh PI, Faulkner GE, Bajaj RR, Silver MA, Mitchell MS, et al. Sedentary Time and Its Association With Risk for Disease Incidence, Mortality, and Hospitalization in Adults. Ann Intern Med. 2015;162:123.

12. Hardin PE. From biological clock to biological rhythms. Genome Biol. 2000;1:reviews1023.1-1023.5.

13. Kizaki T, Sato S, Shirato K, Sakurai T, Ogasawara J, Izawa T, et al. Effect of Circadian Rhythm on Clinical and Pathophysiological Conditions and Inflammation. Crit Rev Immunol. 2015;35:261–75.

14. Baron KG, Reid KJ. Circadian misalignment and health. Int Rev Psychiatry. 2014;26:139–54.

15. Leproult R, Holmbäck U, Van Cauter E, McMenamin T, Suwazono Y, Dochi M, et al. Circadian misalignment augments markers of insulin resistance and inflammation,

For peer review only - http://bmjopen.bmj.com/site/about/guidelines.xhtml

independently of sleep loss. Diabetes. 2014;63:1860–9.

16. Diem SJ, Blackwell TL, Stone KL, Yaffe K, Tranah G, Cauley JA, et al. Measures of Sleep-Wake Patterns and Risk of Mild Cognitive Impairment or Dementia in Older Women. Am J Geriatr Psychiatry. 2016;24:248–58.

17. Haney A, Buysse DJ, Rosario BL, Chen Y-F, Okun ML. Sleep disturbance and cardiometabolic risk factors in early pregnancy: a preliminary study. Sleep Med. 2014;15:444–50.

18. Lynch BM, Boyle T, Winkler E, Occleston J, Courneya KS, Vallance JK. Patterns and correlates of accelerometer-assessed physical activity and sedentary time among colon cancer survivors. Cancer Causes Control. 2016;27:59–68.

19. Palagini L, Gemignani A, Banti S, Manconi M, Mauri M, Riemann D. Chronic sleep loss during pregnancy as a determinant of stress: impact on pregnancy outcome. Sleep Med. 2014;15:853–9.

20. Tsai S-Y, Lin J-W, Wu W-W, Lee C-N, Lee P-L. Sleep Disturbances and Symptoms of Depression and Daytime Sleepiness in Pregnant Women. Birth. 2016;43:176–83.

21. Tsai S-Y, Lee P-L, Lin J-W, Lee C-N. Cross-sectional and Longitudinal Associations between Sleep and Health-related Quality of Life in Pregnant Women: a Prospective Observational Study. Int J Nurs Stud. 2016;56:45–53.

22. Hayase M, Shimada M, Seki H. Sleep quality and stress in women with pregnancy-induced hypertension and gestational diabetes mellitus. Women Birth. 2014;27:190–5.

23. Mindell JA, Cook RA, Nikolovski J. Sleep patterns and sleep disturbances across pregnancy. Sleep Med. 2015;16:483–8.

24. JOHN D, FREEDSON P. ActiGraph and Actical Physical Activity Monitors. Med Sci Sport Exerc. 2012;44 1 Suppl 1:S86–9.

25. Martin JL, Hakim AD. Wrist actigraphy. Chest. 2011;139:1514–27.

26. Wood AC, Kuntsi J, Asherson P, Saudino KJ. Actigraph data are reliable, with functional reliability increasing with aggregation. Behav Res Methods. 2008;40:873–8.

27. Morgenthaler TI, Lee-Chiong T, Alessi C, Friedman L, Aurora RN, Boehlecke B, et al. Practice parameters for the clinical evaluation and treatment of circadian rhythm sleep disorders. An American Academy of Sleep Medicine report. Sleep. 2007;30:1445–59.

28. Sadeh A. The role and validity of actigraphy in sleep medicine: an update. Sleep Med Rev. 2011;15:259–67.

29. Sani M, Refinetti R, Jean-Louis G, Pandi-Perumal SR, Durazo-Arvizu RA, Dugas LR, et al. Daily activity patterns of 2316 men and women from five countries differing in socioeconomic development. Chronobiol Int. 2015;32:650–6.

30. Hart TL, Swartz AM, Cashin SE, Strath SJ. How many days of monitoring predict physical activity and sedentary behaviour in older adults? Int J Behav Nutr Phys Act. 2011;8:62.

31. Falck RS, Landry GJ, Brazendale K, Liu-Ambrose T. Measuring Physical Activity in Older Adults Using MotionWatch 8© Actigraphy: How Many Days are Needed? J Aging Phys Act. 2016.

For peer review only - http://bmjopen.bmj.com/site/about/guidelines.xhtml

32. Dillon CB, Fitzgerald AP, Kearney PM, Perry IJ, Rennie KL, Kozarski R, et al. Number of Days Required to Estimate Habitual Activity Using Wrist-Worn GENEActiv Accelerometer: A Cross-Sectional Study. PLoS One. 2016;11:e0109913.

33. Trost SG, Zheng Y, Wong W-K. Machine learning for activity recognition: hip versus wrist data. Physiol Meas. 2014;35:2183–9.

34. Ellis K, Kerr J, Godbole S, Staudenmayer J, Lanckriet G. Hip and Wrist Accelerometer Algorithms for Free-Living Behavior Classification. Med Sci Sports Exerc. 2016;48:933–40.

35. Rowlands A V, Cliff DP, Fairclough SJ, Boddy LM, Olds TS, Parfitt G, et al. Moving Forward with Backward Compatibility: Translating Wrist Accelerometer Data. Med Sci Sports Exerc. 2016.

36. Koster A, Shiroma EJ, Caserotti P, Matthews CE, Chen KY, Glynn NW, et al. Comparison of Sedentary Estimates between activPAL and Hip- and Wrist-Worn ActiGraph. Med Sci Sports Exerc. 2016;48:1514–22.

37. Haddad SM, Cecatti JG, Parpinelli MA, Souza JP, Costa ML, Sousa MH, et al. From planning to practice: building the national network for the Surveillance of Severe Maternal Morbidity. BMC Public Health. 2011;11:283.

38. Fisher M. A revealing map of the world’s most and least ethnically diverse countries. Washinton Post. 2013. http://www.washingtonpost.com/blogs/worldviews/wp/2013/o5/16/a-revealing-map-of-the-worlds-most-and-least-ethnically-diverse-countries. Accessed 25 Jul 2016.

39. United Nations Development Programme (UNDP). Human Development Index (HDI). Brazil. 2010. http://www.pnud.org.br/Atlas.aspx.

40. Wu J, Sigmund CD. Hypertension: A Disease That Strikes Around the Clock. Hypertension. 2016;67:493–5.

41. Danilack VA, Nunes AP, Phipps MG. Unexpected complications of low-risk pregnancies in the United States. Am J Obstet Gynecol. 2015;212:809.e1-809.e6.

42. Luft CDB, Sanches S de O, Mazo GZ, Andrade A. [Brazilian version of the Perceived Stress Scale: translation and validation for the elderly]. Rev Saude Publica. 2007;41:606–15.

43. Pesce RP, Assis SG, Avanci JQ, Santos NC, Malaquias J V, Carvalhaes R. [Cross-cultural adaptation, reliability and validity of the resilience scale]. Cad Saude Publica. 21:436–48.

44. Passini R, Cecatti JG, Lajos GJ, Tedesco RP, Nomura ML, Dias TZ, et al. Brazilian Multicentre Study on Preterm Birth (EMIP): Prevalence and Factors Associated with Spontaneous Preterm Birth. PLoS One. 2014;9:e109069.

45. Lisonkova S, Joseph KS. Incidence of preeclampsia: risk factors and outcomes associated with early- versus late-onset disease. Am J Obstet Gynecol. 2013;209:544.e1-544.e12.

46. Belton S, O’Brien W, Wickel EE, Issartel J. Patterns of noncompliance in adolescent field-based accelerometer research. J Phys Act Health. 2013;10:1181–5.

47. Ellis K, Kerr J, Godbole S, Lanckriet G, Wing D, Marshall S, et al. A random forest classifier for the prediction of energy expenditure and type of physical activity from wrist and hip accelerometers. Physiol Meas. 2014;35:2191–203.

For peer review only

48. Staudenmayer J, He S, Hickey A, Sasaki J, Freedson P. Methods to estimate aspects of physical activity and sedentary behavior from high-frequency wrist accelerometer measurements. J Appl Physiol. 2015;119.

49. Kamada M, Shiroma EJ, Harris TB, Lee I-M. Comparison of physical activity assessed using hip- and wrist-worn accelerometers. Gait Posture. 2016;44:23–8.

50. Freedson PS, Melanson E, Sirard J. Calibration of the Computer Science and Applications, Inc. accelerometer. Med Sci Sports Exerc. 1998;30:777–81.

51. Tranquilli AL, Dekker G, Magee L, Roberts J, Sibai BM, Steyn W, et al. The classification, diagnosis and management of the hypertensive disorders of pregnancy: A revised statement from the ISSHP. Pregnancy Hypertens. 2014;4:97–104.

52. WHO recommendations on antenatal care for a positive pregnancy experience. World Health Organization. 2016. http://www.who.int/reproductivehealth/publications/maternal_perinatal_health/anc-positive-pregnancy-experience/en/.

53. North RA, McCowan LME, Dekker GA, Poston L, Chan EHY, Stewart AW, et al. Clinical risk prediction for pre-eclampsia in nulliparous women: development of model in international prospective cohort. BMJ. 2011;342:d1875.

54. Murphy NM, McCarthy FP, Khashan AS, Myers JE, Simpson NAB, Kearney PM, et al. Compliance with National Institute of Health and Care Excellence risk-based screening for Gestational Diabetes Mellitus in nulliparous women. Eur J Obstet Gynecol Reprod Biol. 2016;199:60–5.

55. McCowan LME, Roberts CT, Dekker GA, Taylor RS, Chan EHY, Kenny LC, et al. Risk factors for small-for-gestational-age infants by customised birthweight centiles: data from an international prospective cohort study. BJOG. 2010;117:1599–607.

56. Thaddeus S, Maine D. Too far to walk: maternal mortality in context. Soc Sci Med. 1994;38:1091–110.

57. von Elm E, Altman DG, Egger M, Pocock SJ, Gøtzsche PC, Vandenbroucke JP. The Strengthening the Reporting of Observational Studies in Epidemiology (STROBE) statement: guidelines for reporting observational studies. PLoS Med. 2007;4:e296.

58. Huberty J, Ehlers DK, Kurka J, Ainsworth B, Buman M, Gangwisch J, et al. Feasibility of three wearable sensors for 24 hour monitoring in middle-aged women. BMC Womens Health. 2015;15:55.

59. Wright SP, Hall Brown TS, Collier SR, Sandberg K. How consumer physical activity monitors could transform human physiology research. Am J Physiol Regul Integr Comp Physiol. 2017;:ajpregu.00349.2016.

60. Herring SJ, Foster GD, Pien GW, Massa K, Nelson DB, Gehrman PR, et al. Do pregnant women accurately report sleep time? A comparison between self-reported and objective measures of sleep duration in pregnancy among a sample of urban mothers. Sleep Breath. 2013;17:1323–7.

61. Okun ML, Kline CE, Roberts JM, Wettlaufer B, Glover K, Hall M. Prevalence of sleep deficiency in early gestation and its associations with stress and depressive symptoms. J women's Heal. 2013;22:1028–37.

62. Herring SJ, Nelson DB, Pien GW, Homko C, Goetzl LM, Davey A, et al. Objectively measured sleep duration and hyperglycemia in pregnancy. Sleep Med.

For peer review only - http://bmjopen.bmj.com/site/about/guidelines.xhtml

2014;15:51–5.

For peer review only

For peer review only - http://bmjopen.bmj.com/site/about/guidelines.xhtml

Table 1. Participating centers in the Maternal Actigraphy Exploratory Study I (MAES-I)

• The Maternity of CAISM, University of Campinas, in Campinas, SP, Brazil;<br>○ Local Principal Investigator: Maria Laura Costa.
• Maternity of the Clinic Hospital, Federal University of RS, Porto Alegre, RS, Brazil;<br>○ Local Principal Investigator: Janete Vettorazzi.
• Maternity of the University Hospital, Jundiaí Medical School in Jundiaí, SP, Brazil;<br>○ Local Principal Investigator: Ricardo Porto Tedesco.
• Maternity of Clinic Hospital, Federal University of Pernambuco, in Recife, PE, Brazil;<br>○ Local Principal Investigator: Edilberto A Rocha Filho.
• MEAC – School Maternity of the Federal University of Ceará, in Fortaleza, CE, Brazil.<br>○ Local Principal Investigator: Francisco Edson de Lucena Feitosa.

For peer review only

For peer review only - http://bmjopen.bmj.com/site/about/guidelines.xhtml

Table 2. Inclusion and Exclusion Criteria of MAES – I

Inclusion Criteria

- Singleton pregnancy
- Nulliparous (who had never given birth before)
- Between 19+0 – 21+0 weeks of gestation

Exclusion Criteria

• Unsure LMP and unwilling to have to date the Ultrasound	• Major Uterine Anomaly
• ≥ 3 Miscarriages	• Cervical Suture
• Major Fetal Anomaly/Abnormal Karyotype*	• Knife cone biopsy
• Essential Hypertension Treated Pre-pregnancy	• Ruptured membranes
• Mod-Severe Hypertension at booking (≥ 160/100 mmHg) or Chronic hypertension using antihypertensive medication	• Use of long-term steroids
• Pre-pregnancy Diabetes	• Use of Low-dose Aspirin
• Renal Disease	• Use of Calcium (> 1g/24h)
• Systemic Lupus Erythematosus	• Use of Eicosapentaenoic acid (fish oil) > 2,7g
• Anti-phospholipid Syndrome	• Use of Vit. C ≥ 1000mg & Vit. E ≥ 400 UI
• Sickle Cell Disease	• Use of Heparin/LMW Heparin
• HIV or Hep B or Hep C positive	• Untreated Thyroid disease
• Any condition that limits practice of physical activity	• Use of antidepressant and/or anxiolytic agents

* All information regarding fetal anomalies will be properly recorded

For peer review only

For peer review only - http://bmjopen.bmj.com/site/about/guidelines.xhtml

Table 3. Measurement frequency and maximum return periods according to gestational age – MAES I

Gestational age	Measurement frequency	Maximum return period (weeks)
19 – 32 weeks	20Hz	4
33 – 36 weeks	30Hz	2
37 – 42 weeks	50Hz	1

For peer review only

For peer review only - http://bmjopen.bmj.com/site/about/guidelines.xhtml

Table 4. Performance of wrist and hip actigraphy methods according to different activities

	Hip			Wrist		
	**Sens**	**Spec**	**BA**	**Sens**	**Spec**	**BA**
Sitting	0.894	0.923	0.908	0.883	0.870	0.876
Vehicle	0.870	0.987	0.929	0.823	0.964	0.893
Walking/running	0.687	0.981	0.834	0.574	0.983	0.779
Standing	0.797	0.929	0.851	0.687	0.904	0.795
Average	0.812	0.955	0.881	0.742	0.930	0.836

BA: balanced accuracy; sens: sensitivity; spec: specificity

Adapted from Ellis K *et al*. Hip and Wrist Accelerometer Algorithms for Free-Living Behavior Classification. Med. Sci. Sports Exerc. 2016.

For peer review only

For peer review only - http://bmjopen.bmj.com/site/about/guidelines.xhtml

Table 5. Severe Neonatal Morbidity definition according to term/preterm status

**Preterm**	**Term**
Grade III and IV intraventricular haemorrhage	Grade II or III hypoxic ischemic encephalopathy
Chronic lung disease (home on O_2 or on O_2 at 36 weeks gestation	Ventilation>24 hours
Necrotizing enterocolitis	Neonatal intensive care admission >4 days
Retinopathy of prematurity, stage 3 or 4	Apgar score <4 at 5 mins
Sepsis (blood or CSF culture proven)	Cord arterial pH<7.0 and/or base excess>-15
Cystic peri-ventricular leukomalacia	Neonatal seizures

For peer review only

For peer review only - http://bmjopen.bmj.com/site/about/guidelines.xhtml

Figure legends:

Figure 1. Set points of MAES-I study

Figure 2. Estimated prevalence of preterm birth and preeclampsia according to gestational age (RED represents majority of cases) and period of evaluation of PA and sleep patterns (in GREY)

For peer review only

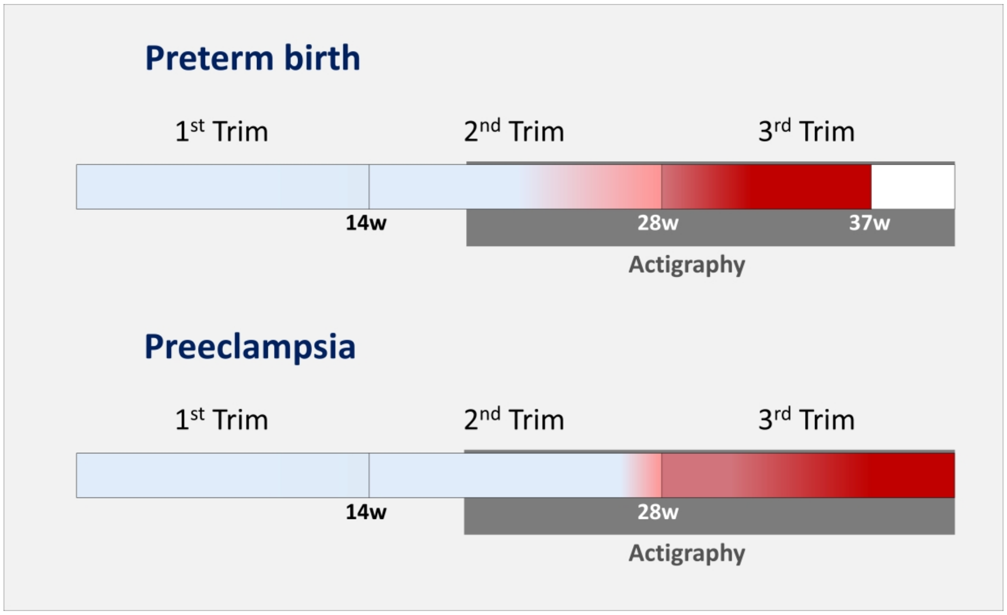

Figure 1

402x244mm (300 x 300 DPI)

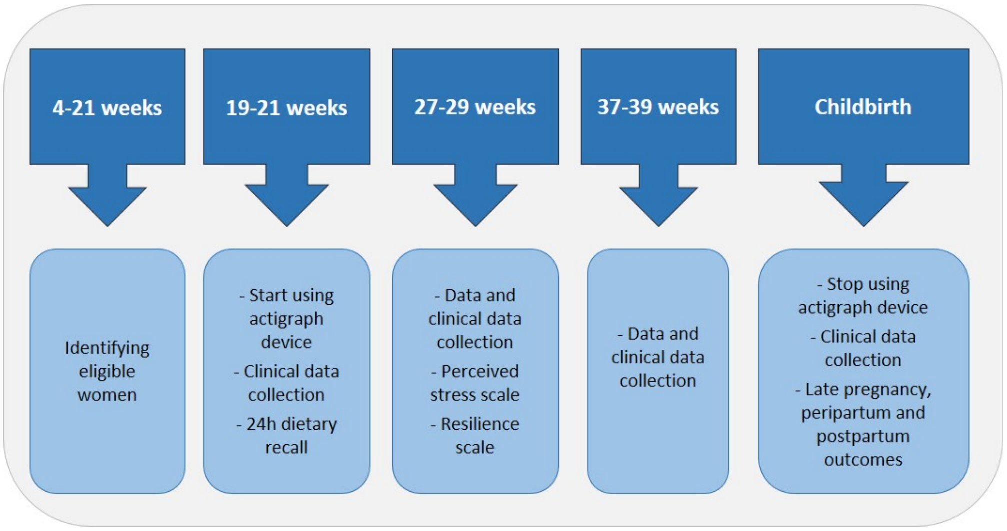

Figure 2

90x47mm (300 x 300 DPI)

STROBE 2007 (v4) checklist of items to be included in reports of observational studies in epidemiology*
Checklist for cohort, case-control, and cross-sectional studies (combined)

Section/Topic	Item #	Recommendation	Reported on page #
**Title and abstract**	1	(*a*) Indicate the study's design with a commonly used term in the title or the abstract	1,2
		(*b*) Provide in the abstract an informative and balanced summary of what was done and what was found	2
**Introduction**			
Background/rationale	2	Explain the scientific background and rationale for the investigation being reported	4-6
Objectives	3	State specific objectives, including any pre-specified hypotheses	6
**Methods**			
Study design	4	Present key elements of study design early in the paper	1,2,7
Setting	5	Describe the setting, locations, and relevant dates, including periods of recruitment, exposure, follow-up, and data collection	7
Participants	6	(*a*) *Case-control study*—Give the eligibility criteria, and the sources and methods of case ascertainment and control selection. Give the rationale for the choice of cases and controls<br>*Cross-sectional study*—Give the eligibility criteria, and the sources and methods of selection of participants	7,8
		(*b*) *Case-control study*—For matched studies, give matching criteria and the number of controls per case	
Variables	7	Clearly define all outcomes, exposures, predictors, potential confounders, and effect modifiers. Give diagnostic criteria, if applicable	11-15
Data sources/ measurement	8*	For each variable of interest, give sources of data and details of methods of assessment (measurement). Describe comparability of assessment methods if there is more than one group	11-15
Bias	9	Describe any efforts to address potential sources of bias	16,17
Study size	10	Explain how the study size was arrived at	15
Quantitative variables	11	Explain how quantitative variables were handled in the analyses. If applicable, describe which groupings were chosen and why	13
Statistical methods	12	(*a*) Describe all statistical methods, including those used to control for confounding	16,17
		(*b*) Describe any methods used to examine subgroups and interactions	16,17
		(*c*) Explain how missing data were addressed	17
		(*d*) *Case-control study*—If applicable, explain how matching of cases and controls was addressed<br>*Cross-sectional study*—If applicable, describe analytical methods taking account of sampling strategy	n/a
		(*e*) Describe any sensitivity analyses	n/a
**Results**			

For peer review only

Participants	13*	(a) Report numbers of individuals at each stage of study—eg numbers potentially eligible, examined for eligibility, confirmed eligible, included in the study, completing follow-up, and analysed	n/a
		(b) Give reasons for non-participation at each stage	n/a
		(c) Consider use of a flow diagram	n/a
Descriptive data	14*	(a) Give characteristics of study participants (eg demographic, clinical, social) and information on exposures and potential confounders	7,8
		(b) Indicate number of participants with missing data for each variable of interest	n/a
Outcome data	15*	*Case-control study*—Report numbers in each exposure category, or summary measures of exposure	n/a
		Cross-sectional study—Report numbers of outcome events or summary measures	n/a
Main results	16	(*a*) Give unadjusted estimates and, if applicable, confounder-adjusted estimates and their precision (eg, 95% confidence interval). Make clear which confounders were adjusted for and why they were included	n/a
		(*b*) Report category boundaries when continuous variables were categorized	n/a
		(*c*) If relevant, consider translating estimates of relative risk into absolute risk for a meaningful time period	
Other analyses	17	Report other analyses done—eg analyses of subgroups and interactions, and sensitivity analyses	17
**Discussion**			
Key results	18	Summarise key results with reference to study objectives	n/a
Limitations	19	Discuss limitations of the study, taking into account sources of potential bias or imprecision. Discuss both direction and magnitude of any potential bias	21
Interpretation	20	Give a cautious overall interpretation of results considering objectives, limitations, multiplicity of analyses, results from similar studies, and other relevant evidence	19-21
Generalisability	21	Discuss the generalisability (external validity) of the study results	21
**Other information**			
Funding	22	Give the source of funding and the role of the funders for the present study and, if applicable, for the original study on which the present article is based	24

*Give information separately for cases and controls in case-control studies and, if applicable, for exposed and unexposed groups in cohort and cross-sectional studies.

Note: An Explanation and Elaboration article discusses each checklist item and gives methodological background and published examples of transparent reporting. The STROBE checklist is best used in conjunction with this article (freely available on the Web sites of PLoS Medicine at http://www.plosmedicine.org/, Annals of Internal Medicine at http://www.annals.org/, and Epidemiology at http://www.epidem.com/). Information on the STROBE Initiative is available at www.strobe-statement.org.

For peer review only

The identification of earlier predictors of pregnancy complications through wearable technologies in a Brazilian multicenter cohort: Maternal Actigraphy Exploratory Study I (MAES-I) study protocol

Journal:	*BMJ Open*
Manuscript ID	bmjopen-2018-023101.R3
Article Type:	Protocol
Date Submitted by the Author:	28-Feb-2019
Complete List of Authors:	Souza, Renato; Universidade Estadual de Campinas, Obstetrics and Gynecology<br>Cecatti, Jose; University of Campinas, Obstetrics and Gynecology<br>Mayrink, Jussara; Universidade Estadual de Campinas Faculdade de Ciencias Medicas<br>Galvão, Rafael; Universidade Estadual de Campinas, Obstetrics and Gynecology<br>Costa, Maria Laura; Universidade Estadual de Campinas, Obstetrics and Gynecology<br>Feitosa, Francisco; Universidade Federal do Ceara, Maternidade Escola<br>Rocha Filho, Edilberto; Universidade Federal de Pernambuco, Obstetrics and Gynecology<br>Leite, Debora; Universidade Federal de Pernambuco, Obstetrics and Gynecology<br>Vettorazzi, Janete; Universidade Federal do Rio Grande do Sul, Obstetrics and Gynecology<br>Tedesco, Ricardo; School of Medicine of Jundiai, Obstetrics and Gynecology<br>Santana, Danielly; Universidade Estadual de Campinas, Obstetrics and Gynecology<br>Souza, Joao Paulo; Universidade de Sao Paulo Faculdade de Medicina de Ribeirao Preto, Social Medicine
**Primary Subject Heading**:	Obstetrics and gynaecology
Secondary Subject Heading:	Obstetrics and gynaecology
Keywords:	wearable technologies, actigraph, physical activity, sleep patterns, pregnancy complications

STUDY PROTOCOL

The identification of earlier predictors of pregnancy complications through wearable technologies in a Brazilian multicenter cohort: Maternal Actigraphy Exploratory Study I (MAES-I) study protocol

Renato T. Souza [1], Jose G. Cecatti [1#], Jussara Mayrink [1], Rafael B. Galvao [1], Maria L. Costa [1], Francisco E. Feitosa [2], Edilberto A Rocha Filho [3], Débora F Leite [1,3], Janete Vettorazzi [4], Ricardo P Tedesco [5], Danielly S Santana [1,5], Joao P. Souza [6], for the MAES-I Study Group*

Affiliations

[1] Department of Obstetrics and Gynaecology, University of Campinas (UNICAMP) School of Medical Sciences, Campinas, SP, Brazil

[2] MEAC – School Maternity of the Federal University of Ceará, in Fortaleza, CE, Brazil

[3] Department of Maternal and Child Health, Maternity of Clinic Hospital, Federal University of Pernambuco, in Recife, PE, Brazil

[4] Department of Obstetrics and Gynaecology, Maternity of the Clinic Hospital, Federal University of RS, Porto Alegre, RS, Brazil

[5] Department of Obstetrics and Gynaecology, Jundiaí Medical School, Jundiaí, SP, Brazil

[6] Department of Social Medicine, Ribeirão Preto Medical School, University of Sao Paulo, Ribeirão Preto, SP, Brazil

*Membership of the MAES-I study group is provided in the Acknowledgments.

E-mail addresses: RT Souza: renatotsouzasp@gmail.com; JG Cecatti: cecatti@unicamp.br; J Mayrink: jussaramayrink@gmail.com; RB Galvão: rafaelbfg@gmail.com; ML Costa: mlaura@unicamp.br; FE Feitosa: edson.lucena@hotmail.com; EA Rocha Filho: edilbertorocha@globo.com; DF Leite: debora.leite@ufpe.br; J Vettorazzi: janetev@terra.com.br; RP Tedesco: rp.tedesco@yahoo.com.br; DS Santana: dany.fmj@terra.com.br; JP Souza: jp.souza@usp.br

#Corresponding Author

JG Cecatti

DO&G, University of Campinas

R. Alexander Fleming, 101

Campinas, SP, 13083-881

Brazil

E-mail: cecatti@unicamp.br

For peer review only - http://bmjopen.bmj.com/site/about/guidelines.xhtml

Abstract

Introduction: Non-invasive tools capable of identifying predictors of maternal complications would be a step forward for improving maternal and perinatal health. There is an association between modification in physical activity (PA) and sleep-wake patterns and the occurrence of inflammatory, metabolic, pathologic conditions related to chronic diseases. The actigraphy device is validated to estimate PA and sleep-wake patterns among pregnant women. In order to extend the window of opportunity to prevent, diagnose and treat specific maternal conditions, would it be possible to use actigraphy data to identify risk factors for the development of adverse maternal outcomes during pregnancy?

Methods and analysis: A cohort will be held in 5 centres from the Brazilian Network for Studies on Reproductive and Perinatal Health. MAES-I will enroll 400 low-risk nulliparous women who will wear the actigraphy device on their wrists day and night (24h/day) uninterruptedly from 19-21 weeks until childbirth. Changes in PA and sleep-wake patterns will be analysed throughout pregnancy, considering ranges in gestational age in women with and without maternal complications such as preeclampsia, preterm birth (spontaneous or provider-initiated), gestational diabetes, maternal haemorrhage during pregnancy, in addition to perinatal outcomes. The plan is to design a predictive model using actigraphy data for screening pregnant women at risk of developing specific adverse maternal and perinatal outcomes.

Ethics and Dissemination: MAES-I study has been reviewed and approved by each Institutional Review Board (IRB) and also by the National Council for Ethics in Research. Detailed information about the study is provided in the Brazilian Cohort website (www.medscinet.com/samba) and findings will be published in the scientific literature and Institutional webpages.

Keywords: wearable technologies; actigraphy; physical activity; sleep patterns; sleep-wake cycle; prediction; pregnancy complications.

For peer review only - http://bmjopen.bmj.com/site/about/guidelines.xhtml

Strengths and limitations of this study

- This multicentre cohort will collect comprehensive data on major maternal and perinatal complications such as pre-eclampsia, small for gestational age/fetal growth restriction, preterm birth and gestational diabetes mellitus.
- Physical activity and sleep patterns will be estimated by an innovative wearable device used in the natural environment of the study subject.
- Physical activity and sleep patterns will be estimated from the beginning of the second half of pregnancy until delivery, covering a wide interval during pregnancy, allowing for the study of changes in PA and sleep patterns throughout pregnancy.
- One possible limitation is the first half of pregnancy at a time when this information was not covered.

For peer review only - http://bmjopen.bmj.com/site/about/guidelines.xhtml

Background

Reducing the global maternal mortality ratio to less than 70 per 100,000 live births by 2030 is one of the targets of the new United Nations Sustainable Development Goals [1]. Multiple challenges need to be tackled to achieve this target, but the 2016-2030 health and development agenda goes well beyond mortality reduction. The aim of the Global Strategy for Women's, Children's and Adolescent's Health is to ensure that every newborn, woman, and child not only survives but thrives. This will only be possible if a transformative agenda centered on innovation is put into action [2].

One of the major challenges lies in optimizing earlier predictors and identifiers of maternal and perinatal complications. Delays in diagnosing and managing maternal complications have been associated with poor outcomes [3]. Decreased self-perception of clinical signs related to maternal complications, difficulties in accessing the health system and poor quality of care may contribute to late identification of complications and a worse prognosis. The development of a non-invasive Antenatal Care (ANC) tool for identifying maternal sub-clinical signs during pregnancy may provide a window of opportunity for an earlier identification of abnormal patterns of physiological parameters related to pregnancy complications. Earlier identification occurs when recognition is made before clinical presentation by standard criteria based on clinical signs, symptoms, and supplementary tests. Shortening the time between the onset of a complication and the initiation of appropriate management enables secondary prevention and reduction of maternal morbidity and mortality [3–7].

Pervasive computing (i.e. the trend towards embedding microprocessors in everyday-life objects so they can generate data) and wearable technology (i.e. clothing and accessories incorporating computer and advanced electronic technologies such as sensor wristbands and/or waistbands) are ubiquitous and can generate a new dataset that requires correlation with pregnancy outcomes. Preterm birth and preeclampsia are two important pregnancy complications that have a relatively long subclinical phase before the appearance of signs or symptoms [8, 9]. It is plausible that during subclinical phases of certain conditions the pattern of physical activity (PA) or sleep-wake rhythm is affected in some way and wearable devices could capture these changes. Although some studies have shown that PA patterns (actigraphy parameters) may be related to

For peer review only - http://bmjopen.bmj.com/site/about/guidelines.xhtml

systemic inflammation and diseases in the general population [10, 11], there is a paucity of published literature that correlates wearable technology data with maternal complications.

The human circadian rhythm is regulated by endogenous physiological mechanisms and environmental stimuli [12]. Solid evidence indicates that modification in circadian rhythm or sleep and PA patterns are underlying conditions related to inflammatory, degenerative and/or metabolic chronic diseases such as diabetes, hypertension, and cancer [13]. Circadian misalignment is defined as inappropriately timed sleep and wake, misplaced feeding periods and modification in physical activity behaviour.

Determining a cause or effect relationship between these modifications and the development of pathological conditions is a complex task. It seems that changes in appetite-stimulating hormones, glucose metabolism, inflammatory markers, and mood are some of the related pathways [13–15]. Leproult *et al.* evaluated the effect of circadian misalignment on metabolic and inflammation markers in cardiovascular disease [15]. Insulin action and release, and also levels of some inflammatory markers that are predictors of cardiovascular disease, were abnormal in individuals with circadian misalignment. The mechanisms involved in the association between changes in PA pattern and pathologic conditions seem to have multiple etiologies. Sani *et al.* assessed circadian rhythms of more than 2,300 African adult descendants. In addition to the evaluation of physical activity itself, the aim of those authors was to identify chronobiological patterns of adults from different socioeconomic settings. The study described that chronobiological behaviour can vary depending on individual BMI, socioeconomic background, work type and time of sunlight exposure. Many other factors, such as pathologic conditions, may be potentially involved in a modification in chronobiological behaviour. Some metabolic, cognitive, cardiovascular and other chronic degenerative diseases have been associated with particular patterns of PA and sleep [10, 11, 16–18]. A previous observational study assessed various sleep parameters during pregnancy, e.g. sleep onset latency (SOL), wake after sleep onset (WASO) and total nocturnal sleep time (TST). Difficulty in initiating sleep in early pregnancy was associated with higher body mass index, greater weight gain and higher blood pressure during pregnancy [17]. Palagini *et al.* reviewed the clinical evidence between chronic

For peer review only - http://bmjopen.bmj.com/site/about/guidelines.xhtml

sleep loss and adverse pregnancy outcomes, discussing common mechanisms of stress system activation [19]. Low-quality evidence suggests an association between sleep loss and prenatal depression, gestational diabetes, preeclampsia, abnormal length of labour, caesarean delivery, abnormal fetal growth, and preterm birth. Those results corroborate with other findings regarding pregnancy and sleep disorders [20–23].

Assessment of PA and sleep patterns can be performed by wearing small wrist (or waist) devices similar to a regular watch (actigraphy technology). More recently, substantial advance has been made in types of sensors, batteries, materials and output data, leading to lower cost, comfort, discretion and performance of the devices [24]. Nowadays, portable, lightweight devices have a large capacity to store data, including software with automatic scoring algorithm packages for the detection of wakefulness, sleep periods and PA [24, 25]. Actigraphy estimation of PA and sleep patterns is validated as a proxy for chronobiological behaviour [26–29] and the use of an actigraphy device for 7 to 14 days provides reliable estimates of PA behaviour in older adults [30–32]. The performance of both hip and wrist devices has been shown to be reliable and acceptable for estimating PA and sleep-wake patterns [33–36].

The main advantages of using wearable devices for actigraphy are non-invasiveness, 24/7 monitoring of PA and circadian patterns, and information about sleep habits and parameters in the natural environment of the subject [24, 25, 28]. We propose an innovative and strategic approach to monitor PA and sleep-wake patterns during pregnancy, establishing a large database comprised of clinical, epidemiological, PA and sleep-wake variables that are potentially capable of composing a prediction model for maternal complications during pregnancy. The main goal of this study is to identify earlier predictors of pregnancy complications by establishing a correlation between data on PA and sleep patterns using wearable devices (sensor wristbands) and maternal and perinatal complications and outcomes.

For peer review only - http://bmjopen.bmj.com/site/about/guidelines.xhtml

Methods/Design

Study design

We will conduct a cohort study of 400 pregnant women using sensor wristbands capable of capturing information on daily physical activity and sleep patterns (exposure). This cohort study will be implemented in 5 ANC clinics linked to obstetric units in 3 different regions of Brazil that are already part of the Brazilian Network for Studies on Reproductive and Perinatal Health [37], as shown in Table 1. During an 8-month period, the ANC clinics will identify cases that are eligible to use the sensor wristband. Wearable technology data will be correlated with the occurrence of pregnancy and childbirth complications and outcomes, such as hypertensive disorders, gestational diabetes mellitus, fetal growth restriction and prematurity.

Eligible women will be identified up to 21 weeks of gestation and invited to participate in the study. A proper consent form will be applied and women who agree to participate will receive a sensor wristband to wear continuously from 19-21 weeks until childbirth.

Study setting and population

Brazil is a multi-ethnic mixed-race population of diverse resourced settings [38]. Despite the high global overall human development index (HDI 0.727) in 2010, the HDI of Brazilian municipalities ranged from 0,862 to 0,418 [39]. A mixed population is suitable for exploring information on patterns of maternal mobility and sleep, maximizing external validity and comparisons to other populations. The following reasons support a study population of low-risk nulliparous women: 1) Previous obstetric history can refer to known risk factors for many maternal complications such as preterm birth, preeclampsia, and diabetes [13, 40]. Therefore, nulliparous women permit unbiased sampling regarding obstetric history. 2) Women with previous morbidities such as hypertension, diabetes, nephropathy or other chronic/degenerative diseases are more likely to present abnormalities in sleep-wake rhythms or physical activity patterns during pregnancy.

For peer review only

Sampling

The five participating centres are regional referral obstetric units responsible for antenatal care of mainly high-risk pregnant women. Participating centres are listed in Table 1. Nevertheless, there are primary health care units strategically linked to these participating centres, enabling the identification and enrolment of women with non-pathological pregnancies. Recruitment strategies include approaching all eligible women in these participating centres and their linked facilities. An informed consent form will be applied for women who agree to participate.

Eligible women: Low-risk pregnant subjects

There is a lack of international consensus on criteria for low-risk pregnancies, although several factors are known to be associated with maternal and perinatal adverse outcomes. A recent study evaluating complications of “low-risk” pregnancies of US Americans (10 million births from 2011 to 2013) indicated that 29% of low-risk women experienced an unexpected complication that required no routine obstetric/neonatal care [41]. This illustrates the difficulty in establishing a “low-risk profile” for maternal/perinatal complications. To make a better identification of eligible low-risk pregnant women, we excluded known potential confounders of pre-pregnancy conditions that could be related to adverse maternal or perinatal outcomes as shown in Table 2, so we could assess PA and sleep patterns of a mostly “normal” population. Nonetheless, features such as lifestyle habits and body composition (body mass index, height), and some non-severe chronic diseases including non-severe anaemia and/or asthma are not exclusion criteria in this study. However, these features and conditions may be a part of subgroup analyses (composition of any previous disorder, e.g.). Intra and inter-individual analyses of PA and sleep patterns can avoid possible bias by identifying potential confounders that may affect primary outcomes. A comparative analysis will be conducted, in which parameters of PA and sleep patterns will be collected in different stages of pregnancy from the same participant (intra-individual analysis) and compared to data collected at the same stage of pregnancy from different participants (inter-individual analysis).

Eligible women are to be enrolled at 04-21 weeks of gestation. Inclusion and exclusion criteria are shown in Table 2.

For peer review only - http://bmjopen.bmj.com/site/about/guidelines.xhtml

Data collection methods

Essentially, MAES I study is comprised of 4 key set points – 3 clinical visits during pregnancy and a postnatal visit. Clinical visits will be held at 1) 19-21 weeks; 2) 27-29 weeks; and 3) 37-39 weeks. On the first, second, third and postnatal visits, additional information on maternal history, details of pregnancy complications, maternal biophysical data (weight, height, skinfolds) and adverse pregnancy outcomes will be collected following a specific Standard Operating Procedure (SOP) specially developed for MAES-I study. Furthermore, the Perceived Stress Scale [42] and Resilience Scale [43] will be applied during the 27-29 weeks visit. Figure 1 shows the set points of MAES-I study.

Eligible women will be invited to use a 43mmx40mmx13mm water-resistant wrist device similar to a regular watch (GENEActiv Original – Activinsights®). The device contains an accelerometer for PA calculation and sensors for estimation of sleep-wake patterns by light and temperature measurements, using a proper software algorithm.

At the first set point of MAES-I study (between 19+0 - 21+0 weeks of gestation), eligible women who agreed to participate will be instructed to wear the wrist bracelet device on the non-dominant wrist night and day (24h/day), uninterruptedly until childbirth (including bathing or recreational water activities). Participants will not need to press any buttons and functioning of the device requires no special care. The device will be configured to register physical activity and sleep-wake data automatically from the moment it is delivered to the participant during antenatal care visit. In addition, the battery charge will be held by the research assistant before delivering the device to the study participant.

The acquisition of actigraphy data can be performed in different frequencies (from 10Hz to 100Hz). Since the frequency of data acquisition has an impact on battery life of the device (inverse relationship), measurement frequency will be set according to gestational age of the participant (Table 3). This information will be registered in the database accordingly. Cumulative data will be downloaded during antenatal care visits,

For peer review only - http://bmjopen.bmj.com/site/about/guidelines.xhtml

according to maximum return periods shown in Table 3. Calculation of maximum return periods will be based on expected battery life. At each antenatal care visit, the used device will be returned to the research team and a new charged device will be provided to the participant.

A leaflet with detailed information and FAQ (Frequently asked questions) about the device will also be provided. Women will also have a cell phone number to call in case of any doubts regarding use of the device, or if any technical or medical concern arises.

During each antenatal care visit, the wrist device will be connected to a charge base which can be connected to a computer through an USB connection. All actigraphy data will be extracted to the computer as raw data ".bin" file. A proper open source software (Geneactiv Software®) will allow the conversion of this file into ".csv" compressed epoch files for each 30 minutes of registered data, which can be read in Excel® program. The actigraphy data will then be uploaded to an online database platform developed by MedSciNet®, where all clinical study data will also be registered.

The actigraphy software uses several algorithms to translate numerical information obtained from epoch files into physical activity and sleep-wake patterns, which will compose the independent variables of this study. This is a centralized, secure, internet-based database that allows several procedures for prospective and retrospective monitoring, hierarchical access (local user, general manager, etc.). The database will be translated into Portuguese and English, facilitating data collection for Portuguese-speaking teams and international monitoring. A correspondent paper form will be available for data collection if necessary (e.g. internet connection failure for instance).

Decision to start monitoring PA and sleep patterns between 19-21weeks

There are various underlying mechanisms involved in the development of maternal and perinatal adverse outcomes that will be assessed, such as preterm birth, preeclampsia, gestational diabetes, fetal growth restriction and small for gestational age. Each disease may have a different pre-clinical phase, depending on environmental and individual aspects. In this phase, there are no clinical signs or symptoms. So far, the study of adverse maternal and perinatal predictors has been focused on early pregnancy (first trimester, <14 weeks of gestation) to maximize the window of opportunity for the

For peer review only - http://bmjopen.bmj.com/site/about/guidelines.xhtml

performance of preventive interventions. However, we hypothesized that modifications in PA and/or sleep pattern due to underlying changes in maternal biological function might not be evident at a very early stage in pregnancy before the beginning of the pre-clinical phase. Our hypothesis is that changes might occur shortly before the manifestation of symptoms.

Furthermore, we took into account that major maternal complications, including preeclampsia, fetal growth restriction, and preterm birth, occur more commonly in late pregnancy and established the period between 19-21 weeks as an appropriate time to start assessment of PA and sleep patterns. A recent cross-sectional study conducted in 20 referral centres in Brazil, including the five participating centres of this proposal, showed that the occurrence of preterm birth before 28 weeks comprised less than 1% of all births and less than 8% of all preterm births [44]. In addition, the early onset of preeclampsia (before 34 weeks of gestation) complicates less than 0.4% of all pregnancies, according to a large retrospective cohort of more than 450,000 deliveries in the USA [45]. Figure 2 outlines the predicted prevalence of preterm birth and preeclampsia in the second trimester. Clinical presentation, when classic symptoms and signs of a certain disease/complication occur, is highlighted by pregnancy in red. Our hypothesis is that alterations in PA and sleep patterns may occur closer to clinical presentation, still in the preclinical phase when there are no symptoms or signs.

Briefly, an exploratory study required an arbitrary decision about the interval for monitoring PA and sleep patterns. To that end, we considered that: 1) the main maternal/perinatal complications of interest occur in the second half of pregnancy, more precisely in late pregnancy (Figure 2); 2) any potential change in PA or sleep patterns occurred hypothetically days or weeks before the onset of maternal or perinatal complications. Then, we focused on monitoring women during the second half of pregnancy.

Thus, starting assessment at 19-21 weeks seems to be quite reasonable, providing a wide interval to monitor and predict major maternal and perinatal adverse outcomes.

Actigraphy device

For peer review only - http://bmjopen.bmj.com/site/about/guidelines.xhtml

The actigraphy device that will be used for monitoring PA and sleep-wake patterns is the GENEActiv Original (GENEActiv, Activinsights Ltd, Huntingdon, UK). The device has multiple sensors including a microelectromechanical (MEMS) accelerometer, temperature (linear active thermistor) and light (silicon photodiode) sensors, providing crude raw data for a variety of applications.

Wrist vs waist wear: advantages and performance

Wrist-worn actigraphy devices are more comfortable to use during wake and sleep periods and provide the highest wear time compared to waist-worn monitors [33, 46]. A non-systematic review published in 2011 showed that actigraphy is a useful and reliable tool to assess sleep patterns and circadian rhythm disorders, although there are some limitations in the diagnosis of sleep disorders or measurement of sleep stages [25]. Actigraphy had a very good concordance with polysomnography for assessment of sleep parameters in healthy subjects (i.e., sensitivity >90% in estimating total sleep time). A recent study evaluated the concordance of physical activity estimation by wrist device in free-living settings in forty overweight or obese women [34]. Those women used both wrist and hip devices, and a small camera that captured participant behaviour for 7 days, monitoring physical activity behaviour (gold-standard comparison). There was a difference in hip and wrist machine learning (ML) classifiers, resulting from different methods/algorithms used to measure physical activity [34]. The sensitivity and specificity of hip and wrist estimations according to Ellis *et al* are shown in Table 4 [34].

Two years previously, the same author published a similar evaluation of 40 adult women and men, showing that hip and wrist accelerometers predicted types of PA with an average accuracy of 92.3% and 87.5% respectively [47].

Staudenmayer *et al* investigated 20 participants who also wore two devices (wrist and hip), and concluded that wrist actigraphy can estimate energy expenditure in an accurate and relatively precise manner [48]. Another study evaluated PA patterns in women at the top 40% or bottom 40% of the distribution of daily PA who wore wrist devices in a free-living environment. There was agreement in classification between hip and wrist accelerometers in about 75% of those women [49]. Additionally, total activity (counts per day) was moderately correlated (Spearman's r = 0.73) with wrist-worn and hip-worn devices.

For peer review only - http://bmjopen.bmj.com/site/about/guidelines.xhtml

To the best of our knowledge, there are no systematic reviews or other high-quality evidence-based recommendations that support a particular method. Although a wrist-worn actigraphy device is not the most traditional method, it might be the best choice for assessment of prolonged periods of PA or sleep patterns, considering that it performs similarly to a waist-worn device. The current proposal has no intention of diagnosing pathological behaviours or diseases, but it plans to identify different patterns throughout pregnancy and in different subgroups of women. Evidence suggests that wrist-worn actigraphy devices can accurately and more comfortably estimate PA and sleep patterns, mainly during prolonged periods and in free-living environments. Therefore, the MAES-I study group adopted a wrist-worn device.

Main variables

Independent variables assessed as potential predictors of maternal complications will be related to the sleep-wake cycle and mobility as:

"Sleep" variables

- Sleep onset latency (SOL): time elapsed between full wakefulness and sleep.
- Total sleep time (TST): The amount of actual sleep time in a sleep episode (excluding time awake).
- Wake after sleep onset (WASO): defined as total amount of time awake after sleep.
- Sleep Efficiency (SE): the ratio between total sleep time and time in bed.

The actigraphy device collects many pieces of information related to body position and body movements to estimate the described sleep variables. The actigraphy software will then be used to analyse data and generate output variables.

"Physical activity" variables

Actigraphy technology estimates physical activity through various parameters collected by the actigraphy device. Briefly, according to Freedson *et al*, the triaxial sensors stressed by acceleration forces can estimate movement intensity. The acceleration signal is converted to a digital signal and summed over a user-specified time interval (epoch). At the end of each epoch the activity count is stored. Then, according to count per minute (CPM) cut points, PA intensity can be categorized [50].

For peer review only - http://bmjopen.bmj.com/site/about/guidelines.xhtml

The software translates information into quantitative variables using appropriate algorithms as follows:

- Sedentary (hours/day): the number of hours per day when the count per minute ranges from 0-99.
- Light activity (hours/day): the number of hours per day when the count per minute ranges from 100 - 1951.
- Moderate activity (minutes/day): the number of hours per day when the count per minute ranges from 1952 - 5724.
- Vigorous activity (minutes/day): the number of hours per day when the count per minute ranges from 5725 - 9498.
- Very vigorous activity (minutes/day): the number of hours per day when the count per minute is 9499 - ∞.
- MET rates: Metabolic Equivalents (METs) are also commonly used to express the intensity of physical activity. One MET is the energy cost of resting quietly, often defined by oxygen uptake as 3.5 $mL \cdot kg^{-1} \cdot min^{-}1$. MET rate expresses the working metabolic rate of subjects in comparison to their resting metabolic rate. Briefly, the triaxial piezoelectric sensors stressed by acceleration forces can estimate movement intensity, converted to oxygen consumption required to perform such a movement.
- Step counts/day: estimated step counts per day (estimated by proper algorithms using accelerometer data.)

Outcomes

Primary outcomes are late pregnancy complications such as:

- Preeclampsia: Hypertension after 20 weeks of gestation, systolic BP ≥ 140mmHg and/or diastolic BP ≥ 90mmHg (Korotkoff V) on at least 2 occasions 4h apart with: 1) Proteinuria 300 mg/24h or spot urine protein: creatinine ratio 30 mg/mmol creatinine or urine dipstick protein ≥ (+) OR, in the absence of proteinuria, hypertension and 2) any multi-system complication that are: Haematological abnormalities; thrombocytopenia (platelets < 100 x 10^9/L); Disseminated

intravascular coagulation (DIC), and haemolysis; Liver disease: increased aspartate transaminase and/or alanine transaminase > 45 IU/L and/or severe right upper quadrant or epigastric pain, liver rupture; Neurological problems: eclampsia, imminent eclampsia (severe headache with hyperreflexia and persistent visual disturbance), cerebral haemorrhage; Acute renal insufficiency: new increase in serum creatinine to > 100 mmol/L antepartum or > 130 mmol/L postpartum; Pulmonary oedema confirmed by chest x-ray [51].

- Gestational Diabetes: new diabetes developing in pregnancy according to the WHO recommendation [52] that defines gestational diabetes as :
  - Fasting plasma glucose ≥ 92 mg/dl, or
  - 1-h plasma glucose tolerance test (75g load) ≥ 180 mg/dl, or
  - 2-h plasma glucose tolerance test (75g load) ≥ 153 mg/dl.
- Spontaneous Preterm Birth: spontaneous onset of preterm labour or premature rupture of membranes leading to preterm birth, childbirth before 37 weeks of gestation.
- Provider-Initiated Preterm Birth: defined as childbirth occurring at less than 37 weeks, medically indicated due to maternal/fetal compromise or both;
- Maternal Hemorrhage: Classified as 1) Antepartum haemorrhage defined as bleeding from the genital tract after 24 weeks of gestation; 2) Primary Postpartum haemorrhage defined as the loss of at least 500 ml blood from the genital tract within 24 hours of childbirth.

Secondary outcomes include childbirth variables and neonatal adverse outcomes such as fetal death, caesarean section, small for gestational age (defined as birth weight below percentile 10 for gestational age), Apgar score < 7 at 5 minutes, neonatal severe morbidity (Table 5) and neonatal mortality before discharge.

Plans for analyses

Sample size estimation

This is an exploratory and innovative study focused on a specific population (pregnant women) and therefore there are no previously published parameters available for sample size estimation. Considering that the rate of pregnancy-related complications is 3 to 20% (including preeclampsia, fetal growth restriction, gestational diabetes,

For peer review only - http://bmjopen.bmj.com/site/about/guidelines.xhtml

hemorrhage, preterm birth, etc.), assuming a large population (above 1 million pregnant women), an acceptable margin of error of 4%, involvement of 5 clusters (participating centres) and a 95% level of confidence, the study would require 384 women. Therefore, we rounded up this estimation to 400 initially low-risk pregnant women for enrolment in the study. We estimated the incidence of some main maternal complications considering the following studies:

- Pre-eclampsia: An international prospective cohort study with nulliparous women termed SCOPE used similar criteria for low-risk profile, with a 5% of incidence of pre-eclampsia [53].
- Preterm Birth: A recent cross-sectional study conducted in 20 referral obstetric centres in Brazil, including the five participating centres, showed that preterm birth was prevalent in 12.3% of all births [44].
- Gestational Diabetes: In the previously mentioned SCOPE international cohort, the prevalence of gestational diabetes was 8.9% in screened low-risk nulliparous women, according to the NICE guidelines [54].
- Fetal growth restriction/small for gestational age: the previously mentioned SCOPE international cohort had a prevalence of 10.7% of small for gestational age newborns, according to customized centiles of birthweight (<10%)[55].

Details of Statistical Analysis

According to the studies above, the predicted incidence of complications seems reasonable and reproducible in our cohort. Therefore, sample size estimation may ensure a sufficient number of cases of maternal and perinatal complications for the current proposal.

The epoch files obtained from Geneactv Software by reading data on sleep variables and physical activity parameters will be translated into numerical results and then averaged in 7-day periods. Therefore, only one value will be employed in statistical analysis for each variable per week of use of the wrist-worn device.

First, we will identify PA and sleep-wake patterns of women who did not develop adverse maternal or perinatal outcomes. This will permit the recognition of normal PA and sleep-wake patterns in a low-risk population without complications during

For peer review only - http://bmjopen.bmj.com/site/about/guidelines.xhtml

pregnancy. We will use the same population to analyse changes in PA and sleep-wake patterns throughout pregnancy, allowing for gestational age periods.

Subsequently, we will compare PA and sleep-wake patterns of women who developed specific adverse maternal or perinatal outcomes with those who did not have any complications. Differences between groups may be identified and used as potential markers for specific pregnancy complications.

Afterwards, we will analyse changes in PA and sleep-wake patterns of women who developed adverse maternal or perinatal outcomes throughout pregnancy, comparing patterns in an attempt to discover which changes occurred before the onset of symptoms that could be related to pregnancy complications. If possible, we will conduct a subgroup analysis including a subpopulation with a potentially higher risk for maternal complications (confounder variables), including obesity, smoking, etc.

Finally, we will develop a predictive model for screening pregnant women at risk of specific adverse maternal and perinatal outcomes using PA and sleep-wake data estimated by actigraphy technology.

Analysis will be performed using the actigraphy software that translates collected information into PA and sleep-wake parameters. In addition, sleep onset latency, wake after sleep onset and total sleep time as well as sleep efficiency will be compared between participants throughout pregnancy using the Friedman and Wilcoxon tests for paired samples. The ANOVA and t-test will be used to compare sleep parameters between participants per week of gestational age for repeated measures. The same tests will be applied to analyse quantitative data on the median number of hours per day that different types of physical activity (sedentary, light, moderate, vigorous and very vigorous) are performed, MET rates and estimate of steps/day through the entire gestational period examined, and the comparison between participants per week of gestational age. Also, we will address the sensitivity, specificity and likelihood ratio for altered PA and sleep patterns or for their changes throughout pregnancy.

Discontinuation of participants

Criteria for discontinuation include:

- Withdrawal of consent;

For peer review only - http://bmjopen.bmj.com/site/about/guidelines.xhtml

- Irregular use of the actigraphy device for prolonged periods, less than 50% of the whole planned time. Information of improper use of the device will be recorded if women notify the MAES-I team. Otherwise, the low level of use of the device will be observed after data discharge during antenatal care visits.
- Loss to follow-up, preventing us from downloading actigraphy data.

Women who decide to withdraw from follow-up care will be called by telephone and asked to return the wrist device. The last visit will be scheduled to regain the wrist monitor and direct the woman to a proper antenatal care service to continue medical consultations.

Data and Sample Quality

All entered data will be prospectively and retrospectively monitored by local research assistants and a global monitor. Internal consistency of variables will be constantly performed by database and error messages are automatically flagged. A local research assistant will be responsible for checking all forms and actigraphy data before locking forms, assuring the good quality of data (i.e. double-checking entered data and checking for inconsistencies between variables). The local principal investigator (PI) will be in charge of signing the case, which will then be incorporated into the final database. The University of Campinas will coordinate, implement and monitor the study in the five participating centres. A general manager and a global monitor are also part of the coordinating team. The local team of each participating centre is comprised of a Local PI and research assistants.

Ethics and Dissemination

MAES-I study focuses on low-risk nulliparous Brazilian pregnant women. Although classified as low risk for maternal and perinatal complications, these women are not free from suffering complications. Furthermore, first and second delays, defined as a delay in deciding to seek care and delay in reaching a health care facility [56], are not uncommon. A barrier is created between earlier recognition of symptoms and timely intervention for the successful treatment of potentially life-threatening conditions. We believe that women will feel encouraged, empowered and willing to participate in a study aimed at developing a potentially useful prenatal care tool to identify the risk for

For peer review only - http://bmjopen.bmj.com/site/about/guidelines.xhtml

maternal and perinatal morbidity and mortality. Following national ethical regulations, the participants will not receive any financial compensation.

Women who agree to participate in the study will not have any disadvantage or difficulties in prenatal care. On the contrary, they will receive a contact number to find clinical researchers at any time (24/7 service), maintaining a closer contact with researchers and care providers. The MAES-I team is committed to contact health care providers if any potential complication arises.

Participating women will not be held accountable for any loss, theft or damage to the wrist device. These women will only be required to wear the device as a regular wrist-watch and no self-damage is expected.

Participating women will not be able to identify any PA or sleep parameters at any stage of the study. Data can only be downloaded through proper licensed software of the device. The actigraphy devices provided to participating women have a unique code which will be recorded in the database along with the interval of use per woman. Actigraphy data will be labelled using participant ID, device number, gestational age when the device was initially used and the return date of each device. Codes, ID number and numbers will ensure confidentiality of all participating women. The identity of all women will be kept confidential.

MAES-I study has been reviewed and approved by the National Committee for Ethics in Research of Brazil (CONEP) and by the Institutional Review Board (IRB) of the coordinating centre (Letter of approval 1.834.116 issued on 24th November 2016) and of all other Brazilian participating centres. All women enroled in the MAES-I cohort will sign an informed consent form.

Ethical principles of the Brazilian National Heath Council (Resolution CNS 466/12) will be upheld at every stage of this study. Anonymity of the source of information will be guaranteed and the woman will receive care irrespective of her agreement to participate in the study. The study also complies with the Declaration of Helsinki amended in Hong Kong in 1989. Methodological and ethical aspects of MAES-I study protocol were developed following STROBE guidelines [57].

For peer review only - http://bmjopen.bmj.com/site/about/guidelines.xhtml

Patient and Public Involvement

Patients and the public were not involved in this study for the development of the research question and outcome measures. However, the choice of a wrist device was based on user preference as reported. Participants of the study will have access to information available at the open-access website.

Detailed information about the study is provided in the Brazilian Cohort website (www.medscinet.com/samba). Publications of the results of the study can be found in the scientific literature and Institutional webpages. We intend to disseminate our findings to a scientific peer-reviewed journal, general free access website, specialist conferences, and our funding agencies.

Discussion

Actigraphy is an innovative, non-invasive, non-operator dependent, wearable technology, that is capable of measuring diverse variables related to mobility, physical activity, sleep-wake, and circadian cycle patterns under real-life conditions. Actigraphy devices have a high sensitivity in detecting sleep-wake parameters and are currently highly recommended by the American Sleep Disorder Association for diagnosis and therapy response of circadian rhythm disorders [27, 28, 58]. Although some studies show that using the actigraphy device for 7 to 14 days provides reliable estimates of physical activity behaviour in older adults, it is not absolutely clear how many days are needed to estimate habitual PA by using the wrist/waist device during pregnancy. In general, it seems to depend mainly on the type of actigraphy device, wear location and target population [30, 33]. Nevertheless, MAES-I study will provide sufficient data to assess different patterns throughout pregnancy.

The use of wearable physical activity monitors has increased considerably, owing to interest in the relationship between the pathophysiology of diseases and patterns of physical activity and sleep. A recent study on the use of physical activity monitors in human physiology research unravels current and potential use of the actigraphy device. The device can be applied in strategies that promote a healthier behaviour or predict outcomes [59]. The authors conclude that physical activity monitors, as well as other

For peer review only - http://bmjopen.bmj.com/site/about/guidelines.xhtml

new 21st century technologies, have already transformed physiology research, revolutionizing how we assess patients and opening new areas of interest. In addition, the use of objective measures to evaluate habitual sleep duration and outcomes in pregnancy is critical, considering recent reports of little agreement between objective and subjective assessments of sleep time [60].

Alterations in sleep patterns, including less deep sleep and more nocturnal awakenings can be observed in pregnancy as early as in 10-12 weeks gestation [61]. Sleep disturbances during pregnancy have been associated with preterm delivery, gestational hypertensive disorders, glucose intolerance and increased risk of caesarean delivery [19]. Shortened nocturnal sleep time was also associated with hyperglycemia [62]. Persistent sleep deprivation has been correlated with depressive symptoms and stress perception by pregnant women [61]. These studies explored a correlation between PA patterns and sleep disturbances that determine complications through a well-established relationship between cause and effect. However, this correlation could not always be adequately determined due to study design [17].

In a distinct manner, the intent of our analysis is to discover whether a maternal complication can be identified before the manifestation of its clinical signs, by evaluating physical activity and/or sleep patterns modifications of pregnant women. Considering existing evidence, we speculate that patterns of PA and/or sleep change days or weeks before clinical presentation of the complication. In general, the signs and symptoms of some maternal outcomes are part of the gold-standard criteria for diagnosis (high blood pressure, proteinuria and/or edema in preeclampsia; premature contractions and cervical ripening/dilation in preterm birth; abnormal placental blood flow and insufficient fetal growth in Intrauterine Growth Restriction). We acknowledge that there are potential confounders and limitations in predicting maternal and perinatal complications using PA and sleep patterns estimated by actigraphy devices. The population in our research is expected to have different subgroups of women with different risks and associated factors contributing to maternal complications, such as obesity, smoking habit, and with age under twenty or over forty years old, for instance. None of those factors was considered an exclusion criterion. If possible, we intend to conduct a subgroup analysis of the maternal subgroups, since they may have different

For peer review only - http://bmjopen.bmj.com/site/about/guidelines.xhtml

PA and sleep patterns. Nonetheless, we decided to adopt a pragmatic approach and not exclude such a common factor from our sample.

The use of actigraphy device during prenatal visits has the potential to become a new tool for monitoring pregnant women. It may improve maternal health care and identify altered PA and/or sleep patterns. Changes can be objectively measured by actigraphy before the occurrence of signs and symptoms. The focus is on providing new technology to monitor the development of potential maternal complications. Other positive points in our study are the data collection period (from 19 weeks until delivery) and the low-risk profile of the cohort, enabling us to describe PA and sleep patterns in a low-risk pregnant population and make a better interpretation of actigraphy data among pregnant women. Current clinical and biological predictors of major maternal complications such as preeclampsia, preterm birth, maternal haemorrhage, and gestational diabetes still lack effective sensitivity and specificity.

If our hypothesis is confirmed, this will be an important step for introducing non-invasive screening procedures into prenatal care to identify women at higher risk for those conditions. Women could receive specific advice on the prevention and earlier detection of the condition, take immediate action and seek professional health care to receive appropriate treatment. This would avoid delays, the most significant factors contributing to low-quality health care in underprivileged women, which increase the still substantial burden of maternal morbidity and mortality. If we succeed in identifying "specific patterns of physical activity and sleep" that are predictors of pregnancy complications, further validation studies are recommended to assess the effectiveness of screening procedures in management of these conditions. In addition, MAES-I will permit further specific studies among a high-risk population and also help to identify the best gestational age for monitoring, targeting a specific gestational age interval.

For peer review only - http://bmjopen.bmj.com/site/about/guidelines.xhtml

Abbreviations

ANC – antenatal care

BMI – body mass index

BP – blood pressure

CPM – count per minute

dL – decilitre

FAQ – frequently asked questions

h – hour

HDI – human development index

Hz - hertz

Kg – kilogram

L – litre

MAES-I – maternal Actigraphy Exploratory Study I

MEMS – microelectromechanical

MET – metabolic equivalent

METs – metabolic equivalents

min – minutes

mg – milligram

mL – millilitre

mmol – millimole

NICE – National Institute for Health and Care Excellence

PA – physical activity

PI – Principal investigator

SE – sleep efficiency

SCOPE – SCreening Of Pregnancy Endpoints

SOL – sleep onset latency

TST – total nocturnal sleep time

US – United States

USA – United States of America

w – week

For peer review only

Competing interests

The authors declare that they have no competing interests.

Author contributions

All authors contributed to the overall study design and specific methodologies. RTS, JGC, JM, MLC and JPS conceived the study design and wrote de first version of the study protocol. In a first meeting, the protocol was discussed and incorporated suggestions from RBG, FEF, ERF, DFL, JV, RPT and DSS. RTS, JGC, JM, and RBG planned the implementation of the study and developed the necessary material. RTS, JM, MLC and JGC drafted the manuscript that was afterwards revised by all other authors who gave suggestions. All authors discussed and made important contributions to the manuscript, read and approved the final version for submission.

Acknowledgements

The MAES - I study group also included: Carina B Luiz and Luiza C Brust, School of Medicine, Federal University of Rio Grande do Sul, Porto Alegre, Brazil; Danilo Anacleto and Lívia C Nascimento, School of Medicine, Federal University of Pernambuco, Recife, Brazil; Daisy Lucena and Denise Ellen F Cordeiro, School of Medicine, Federal University of Ceará, Fortaleza, Brazil; Mariana B Rogerio, Departament of Obstetrics and Gynecology, Jundiaí Medical School, Jundiaí, Brazil.

Funding

This study was funded by The Bill and Melinda Gates Foundation through the Grand Challenge Exploration program, call 19 (research grant OPP1182749).

For peer review only

For peer review only - http://bmjopen.bmj.com/site/about/guidelines.xhtml

References

1. United Nations Development Programme (UNDP). Sustainable Development Goals. 2015. http://www.un.org/sustainabledevelopment/health/. Accessed 27 Apr 2016.

2. WHO, World Health Organization. Global Strategy. Global Strategy for Women's, Children's and Adolescents' Health, 2016 - 2030. 2015. http://www.who.int/life-course/partners/global-strategy/global-strategy-2016-2030/en/. Accessed 9 Jul 2016.

3. Pacagnella RC, Cecatti JG, Parpinelli MA, Sousa MH, Haddad SM, Costa ML, et al. Delays in receiving obstetric care and poor maternal outcomes: results from a national multicentre cross-sectional study. BMC Pregnancy Childbirth. 2014;14:159.

4. Haddad SM, Cecatti JG, Souza JP, Sousa MH, Parpinelli MA, Costa ML, et al. Applying the maternal near miss approach for the evaluation of quality of obstetric care: a worked example from a Multicenter Surveillance Study. Biomed Res Int. 2014;2014:989815.

5. Pacagnella RC, Cecatti JG, Osis MJ, Souza JP. The role of delays in severe maternal morbidity and mortality: expanding the conceptual framework. Reprod Health Matters. 2012;20:155–63.

6. Vogel JP, Souza JP, Mori R, Morisaki N, Lumbiganon P, Laopaiboon M, et al. Maternal complications and perinatal mortality: findings of the World Health Organization Multicountry Survey on Maternal and Newborn Health. BJOG. 2014;121 Suppl:76–88.

7. Souza JP, Gülmezoglu AM, Vogel J, Carroli G, Lumbiganon P, Qureshi Z, et al. Moving beyond essential interventions for reduction of maternal mortality (the WHO Multicountry Survey on Maternal and Newborn Health): a cross-sectional study. Lancet. 2013;381:1747–55.

8. Brosens I, Pijnenborg R, Vercruysse L, Romero R. The "Great Obstetrical Syndromes" are associated with disorders of deep placentation. Am J Obstet Gynecol. 2011;204:193–201.

9. Di Renzo GC. The great obstetrical syndromes. J Matern neonatal Med. 2009;22:633–5.

10. Loprinzi PD. The effects of objectively-measured, free-living daily ambulatory movement on mortality in a national sample of adults with diabetes. Physiol Behav. 2016;154:126–8.

11. Biswas A, Oh PI, Faulkner GE, Bajaj RR, Silver MA, Mitchell MS, et al. Sedentary Time and Its Association With Risk for Disease Incidence, Mortality, and Hospitalization in Adults. Ann Intern Med. 2015;162:123.

12. Hardin PE. From biological clock to biological rhythms. Genome Biol. 2000;1:reviews1023.1-1023.5.

13. Kizaki T, Sato S, Shirato K, Sakurai T, Ogasawara J, Izawa T, et al. Effect of Circadian Rhythm on Clinical and Pathophysiological Conditions and Inflammation. Crit Rev Immunol. 2015;35:261–75.

14. Baron KG, Reid KJ. Circadian misalignment and health. Int Rev Psychiatry. 2014;26:139–54.

15. Leproult R, Holmbäck U, Van Cauter E, McMenamin T, Suwazono Y, Dochi M, et al. Circadian misalignment augments markers of insulin resistance and inflammation,

For peer review only - http://bmjopen.bmj.com/site/about/guidelines.xhtml

independently of sleep loss. Diabetes. 2014;63:1860–9.

16. Diem SJ, Blackwell TL, Stone KL, Yaffe K, Tranah G, Cauley JA, et al. Measures of Sleep-Wake Patterns and Risk of Mild Cognitive Impairment or Dementia in Older Women. Am J Geriatr Psychiatry. 2016;24:248–58.

17. Haney A, Buysse DJ, Rosario BL, Chen Y-F, Okun ML. Sleep disturbance and cardiometabolic risk factors in early pregnancy: a preliminary study. Sleep Med. 2014;15:444–50.

18. Lynch BM, Boyle T, Winkler E, Occleston J, Courneya KS, Vallance JK. Patterns and correlates of accelerometer-assessed physical activity and sedentary time among colon cancer survivors. Cancer Causes Control. 2016;27:59–68.

19. Palagini L, Gemignani A, Banti S, Manconi M, Mauri M, Riemann D. Chronic sleep loss during pregnancy as a determinant of stress: impact on pregnancy outcome. Sleep Med. 2014;15:853–9.

20. Tsai S-Y, Lin J-W, Wu W-W, Lee C-N, Lee P-L. Sleep Disturbances and Symptoms of Depression and Daytime Sleepiness in Pregnant Women. Birth. 2016;43:176–83.

21. Tsai S-Y, Lee P-L, Lin J-W, Lee C-N. Cross-sectional and Longitudinal Associations between Sleep and Health-related Quality of Life in Pregnant Women: a Prospective Observational Study. Int J Nurs Stud. 2016;56:45–53.

22. Hayase M, Shimada M, Seki H. Sleep quality and stress in women with pregnancy-induced hypertension and gestational diabetes mellitus. Women Birth. 2014;27:190–5.

23. Mindell JA, Cook RA, Nikolovski J. Sleep patterns and sleep disturbances across pregnancy. Sleep Med. 2015;16:483–8.

24. JOHN D, FREEDSON P. ActiGraph and Actical Physical Activity Monitors. Med Sci Sport Exerc. 2012;44 1 Suppl 1:S86–9.

25. Martin JL, Hakim AD. Wrist actigraphy. Chest. 2011;139:1514–27.

26. Wood AC, Kuntsi J, Asherson P, Saudino KJ. Actigraph data are reliable, with functional reliability increasing with aggregation. Behav Res Methods. 2008;40:873–8.

27. Morgenthaler TI, Lee-Chiong T, Alessi C, Friedman L, Aurora RN, Boehlecke B, et al. Practice parameters for the clinical evaluation and treatment of circadian rhythm sleep disorders. An American Academy of Sleep Medicine report. Sleep. 2007;30:1445–59.

28. Sadeh A. The role and validity of actigraphy in sleep medicine: an update. Sleep Med Rev. 2011;15:259–67.

29. Sani M, Refinetti R, Jean-Louis G, Pandi-Perumal SR, Durazo-Arvizu RA, Dugas LR, et al. Daily activity patterns of 2316 men and women from five countries differing in socioeconomic development. Chronobiol Int. 2015;32:650–6.

30. Hart TL, Swartz AM, Cashin SE, Strath SJ. How many days of monitoring predict physical activity and sedentary behaviour in older adults? Int J Behav Nutr Phys Act. 2011;8:62.

31. Falck RS, Landry GJ, Brazendale K, Liu-Ambrose T. Measuring Physical Activity in Older Adults Using MotionWatch 8© Actigraphy: How Many Days are Needed? J Aging Phys Act. 2016.

For peer review only - http://bmjopen.bmj.com/site/about/guidelines.xhtml

32. Dillon CB, Fitzgerald AP, Kearney PM, Perry IJ, Rennie KL, Kozarski R, et al. Number of Days Required to Estimate Habitual Activity Using Wrist-Worn GENEActiv Accelerometer: A Cross-Sectional Study. PLoS One. 2016;11:e0109913.

33. Trost SG, Zheng Y, Wong W-K. Machine learning for activity recognition: hip versus wrist data. Physiol Meas. 2014;35:2183–9.

34. Ellis K, Kerr J, Godbole S, Staudenmayer J, Lanckriet G. Hip and Wrist Accelerometer Algorithms for Free-Living Behaviour Classification. Med Sci Sports Exerc. 2016;48:933–40.

35. Rowlands A V, Cliff DP, Fairclough SJ, Boddy LM, Olds TS, Parfitt G, et al. Moving Forward with Backward Compatibility: Translating Wrist Accelerometer Data. Med Sci Sports Exerc. 2016.

36. Koster A, Shiroma EJ, Caserotti P, Matthews CE, Chen KY, Glynn NW, et al. Comparison of Sedentary Estimates between activPAL and Hip- and Wrist-Worn ActiGraph. Med Sci Sports Exerc. 2016;48:1514–22.

37. Haddad SM, Cecatti JG, Parpinelli MA, Souza JP, Costa ML, Sousa MH, et al. From planning to practice: building the national network for the Surveillance of Severe Maternal Morbidity. BMC Public Health. 2011;11:283.

38. Fisher M. A revealing map of the world’s most and least ethnically diverse countries. Washinton Post. 2013. http://www.washingtonpost.com/blogs/worldviews/wp/2013/o5/16/a-revealing-map-of-the-worlds-most-and-least-ethnically-diverse-countries. Accessed 25 Jul 2016.

39. United Nations Development Programme (UNDP). Human Development Index (HDI). Brazil. 2010. http://www.pnud.org.br/Atlas.aspx.

40. Wu J, Sigmund CD. Hypertension: A Disease That Strikes Around the Clock. Hypertension. 2016;67:493–5.

41. Danilack VA, Nunes AP, Phipps MG. Unexpected complications of low-risk pregnancies in the United States. Am J Obstet Gynecol. 2015;212:809.e1-809.e6.

42. Luft CDB, Sanches S de O, Mazo GZ, Andrade A. [Brazilian version of the Perceived Stress Scale: translation and validation for the elderly]. Rev Saude Publica. 2007;41:606–15.

43. Pesce RP, Assis SG, Avanci JQ, Santos NC, Malaquias J V, Carvalhaes R. [Cross-cultural adaptation, reliability and validity of the resilience scale]. Cad Saude Publica. 21:436–48.

44. Passini R, Cecatti JG, Lajos GJ, Tedesco RP, Nomura ML, Dias TZ, et al. Brazilian Multicentre Study on Preterm Birth (EMIP): Prevalence and Factors Associated with Spontaneous Preterm Birth. PLoS One. 2014;9:e109069.

45. Lisonkova S, Joseph KS. Incidence of preeclampsia: risk factors and outcomes associated with early- versus late-onset disease. Am J Obstet Gynecol. 2013;209:544.e1-544.e12.

46. Belton S, O’Brien W, Wickel EE, Issartel J. Patterns of noncompliance in adolescent field-based accelerometer research. J Phys Act Health. 2013;10:1181–5.

47. Ellis K, Kerr J, Godbole S, Lanckriet G, Wing D, Marshall S, et al. A random forest classifier for the prediction of energy expenditure and type of physical activity from wrist and hip accelerometers. Physiol Meas. 2014;35:2191–203.

For peer review only - http://bmjopen.bmj.com/site/about/guidelines.xhtml

48. Staudenmayer J, He S, Hickey A, Sasaki J, Freedson P. Methods to estimate aspects of physical activity and sedentary behavior from high-frequency wrist accelerometer measurements. J Appl Physiol. 2015;119.

49. Kamada M, Shiroma EJ, Harris TB, Lee I-M. Comparison of physical activity assessed using hip- and wrist-worn accelerometers. Gait Posture. 2016;44:23–8.

50. Freedson PS, Melanson E, Sirard J. Calibration of the Computer Science and Applications, Inc. accelerometer. Med Sci Sports Exerc. 1998;30:777–81.

51. Tranquilli AL, Dekker G, Magee L, Roberts J, Sibai BM, Steyn W, et al. The classification, diagnosis and management of the hypertensive disorders of pregnancy: A revised statement from the ISSHP. Pregnancy Hypertens. 2014;4:97–104.

52. WHO recommendations on antenatal care for a positive pregnancy experience. World Health Organization. 2016. http://www.who.int/reproductivehealth/publications/maternal_perinatal_health/anc-positive-pregnancy-experience/en/.

53. North RA, McCowan LME, Dekker GA, Poston L, Chan EHY, Stewart AW, et al. Clinical risk prediction for pre-eclampsia in nulliparous women: development of model in international prospective cohort. BMJ. 2011;342:d1875.

54. Murphy NM, McCarthy FP, Khashan AS, Myers JE, Simpson NAB, Kearney PM, et al. Compliance with National Institute of Health and Care Excellence risk-based screening for Gestational Diabetes Mellitus in nulliparous women. Eur J Obstet Gynecol Reprod Biol. 2016;199:60–5.

55. McCowan LME, Roberts CT, Dekker GA, Taylor RS, Chan EHY, Kenny LC, et al. Risk factors for small-for-gestational-age infants by customised birthweight centiles: data from an international prospective cohort study. BJOG. 2010;117:1599–607.

56. Thaddeus S, Maine D. Too far to walk: maternal mortality in context. Soc Sci Med. 1994;38:1091–110.

57. von Elm E, Altman DG, Egger M, Pocock SJ, Gøtzsche PC, Vandenbroucke JP. The Strengthening the Reporting of Observational Studies in Epidemiology (STROBE) statement: guidelines for reporting observational studies. PLoS Med. 2007;4:e296.

58. Huberty J, Ehlers DK, Kurka J, Ainsworth B, Buman M, Gangwisch J, et al. Feasibility of three wearable sensors for 24 hour monitoring in middle-aged women. BMC Womens Health. 2015;15:55.

59. Wright SP, Hall Brown TS, Collier SR, Sandberg K. How consumer physical activity monitors could transform human physiology research. Am J Physiol Regul Integr Comp Physiol. 2017;:ajpregu.00349.2016.

60. Herring SJ, Foster GD, Pien GW, Massa K, Nelson DB, Gehrman PR, et al. Do pregnant women accurately report sleep time? A comparison between self-reported and objective measures of sleep duration in pregnancy among a sample of urban mothers. Sleep Breath. 2013;17:1323–7.

61. Okun ML, Kline CE, Roberts JM, Wettlaufer B, Glover K, Hall M. Prevalence of sleep deficiency in early gestation and its associations with stress and depressive symptoms. J Women's Heal. 2013;22:1028–37.

62. Herring SJ, Nelson DB, Pien GW, Homko C, Goetzl LM, Davey A, et al. Objectively measured sleep duration and hyperglycemia in pregnancy. Sleep Med.

For peer review only - http://bmjopen.bmj.com/site/about/guidelines.xhtml

2014;15:51–5.

For peer review only

For peer review only - http://bmjopen.bmj.com/site/about/guidelines.xhtml

Table 1. Participating centres in the Maternal Actigraphy Exploratory Study I (MAES-I)

• The Maternity of CAISM, University of Campinas, in Campinas, SP, Brazil;<br>○ Local Principal Investigator: Maria Laura Costa.
• Maternity of the Clinic Hospital, Federal University of RS, Porto Alegre, RS, Brazil;<br>○ Local Principal Investigator: Janete Vettorazzi.
• Maternity of the University Hospital, Jundiaí Medical School in Jundiaí, SP, Brazil;<br>○ Local Principal Investigator: Ricardo Porto Tedesco.
• Maternity of Clinic Hospital, Federal University of Pernambuco, in Recife, PE, Brazil;<br>○ Local Principal Investigator: Edilberto A Rocha Filho.
• MEAC – School Maternity of the Federal University of Ceará, in Fortaleza, CE, Brazil.<br>○ Local Principal Investigator: Francisco Edson de Lucena Feitosa.

For peer review only

For peer review only - http://bmjopen.bmj.com/site/about/guidelines.xhtml

Table 2. Inclusion and Exclusion Criteria of MAES – I

**Inclusion Criteria**
• Singleton pregnancy<br>• Nulliparous (who had never given birth before)<br>• Between 19+0 – 21+0 weeks of gestation

**Exclusion Criteria**	
• Unsure LMP and unwilling to date the Ultrasound	• Major Uterine Anomaly
• ≥ 3 Miscarriages	• Cervical Suture
• Major Fetal Anomaly/Abnormal Karyotype*	• Knife cone biopsy
• Essential Hypertension treated before pregnancy	• Ruptured membranes
• Mod-Severe Hypertension at booking (≥ 160/100 mmHg) or Chronic hypertension using antihypertensive medication	• Use of long-term steroids
• Pre-pregnancy Diabetes	• Use of Low-dose Aspirin
• Renal Disease	• Use of Calcium (> 1g/24h)
• Systemic Lupus Erythematosus	• Use of Eicosapentaenoic acid (fish oil) > 2,7g
• Anti-phospholipid Syndrome	• Use of Vit. C ≥ 1000mg & Vit. E ≥ 400 UI
• Sickle Cell Disease	• Use of Heparin/LMW Heparin
• HIV or Hep B or Hep C positive	• Untreated Thyroid disease
• Any condition that limits the performance of physical activity	• Use of antidepressant and/or anxiolytic agents

* All information on fetal anomalies will be properly recorded

For peer review only

For peer review only - http://bmjopen.bmj.com/site/about/guidelines.xhtml

Table 3. Measurement frequency and maximum return periods according to gestational age – MAES I

Gestational age	Measurement frequency	Maximum return period (weeks)
19 – 32 weeks	20Hz	4
33 – 36 weeks	30Hz	2
37 – 42 weeks	50Hz	1

For peer review only

For peer review only - http://bmjopen.bmj.com/site/about/guidelines.xhtml

Table 4. Performance of wrist and hip actigraphy methods according to different activities

	Hip			Wrist		
	**Sens**	**Spec**	**BA**	**Sens**	**Spec**	**BA**
Sitting	0.894	0.923	0.908	0.883	0.870	0.876
Vehicle	0.870	0.987	0.929	0.823	0.964	0.893
Walking/running	0.687	0.981	0.834	0.574	0.983	0.779
Standing	0.797	0.929	0.851	0.687	0.904	0.795
Average	0.812	0.955	0.881	0.742	0.930	0.836

BA: balanced accuracy; sens: sensitivity; spec: specificity

Adapted from Ellis K *et al*. Hip and Wrist Accelerometer Algorithms for Free-Living Behaviour Classification. Med. Sci. Sports Exerc. 2016.

For peer review only

For peer review only - http://bmjopen.bmj.com/site/about/guidelines.xhtml

Table 5. Severe Neonatal Morbidity definition according to term/preterm status

**Preterm**	**Term**
Grade III and IV intraventricular haemorrhage	Grade II or III hypoxic ischemic encephalopathy
Chronic lung disease (home O_2 therapy or O_2 therapy at 36 weeks gestation	Ventilation>24 hours
Necrotizing enterocolitis	Neonatal intensive care admission >4 days
Retinopathy of prematurity, stage 3 or 4	Apgar score <4 at 5 mins
Sepsis (blood or CSF culture proven)	Cord arterial pH<7.0 and/or base excess>-15
Cystic peri-ventricular leukomalacia	Neonatal seizures

For peer review only

For peer review only - http://bmjopen.bmj.com/site/about/guidelines.xhtml

Figure legends:

Figure 1. Set points of MAES-I study

Figure 2. Estimated prevalence of preterm birth and preeclampsia according to gestational age (RED represents the majority of cases) and evaluation period of PA and sleep patterns (in GREY)

For peer review only

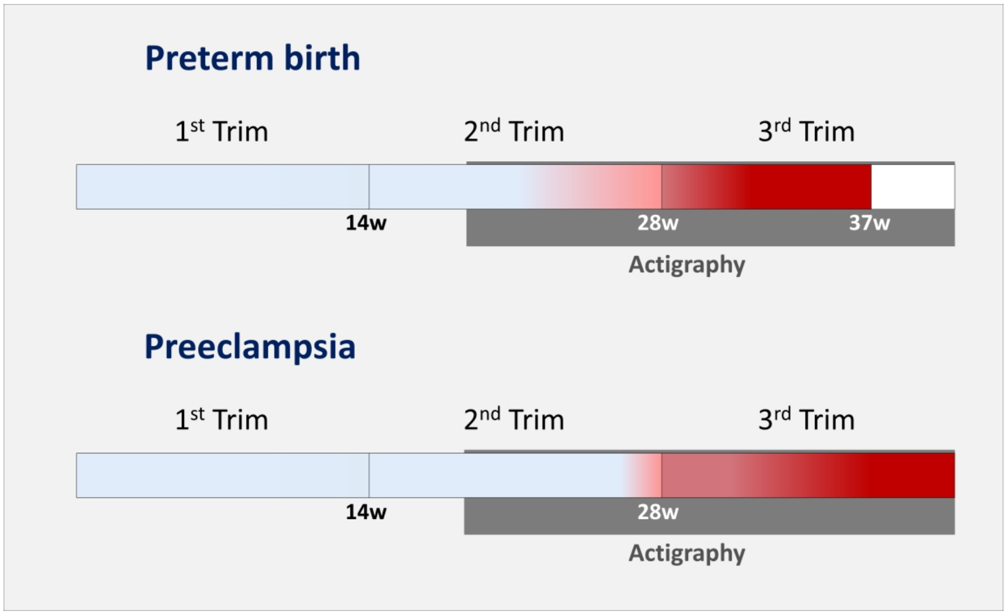

Figure 1

402x244mm (300 x 300 DPI)

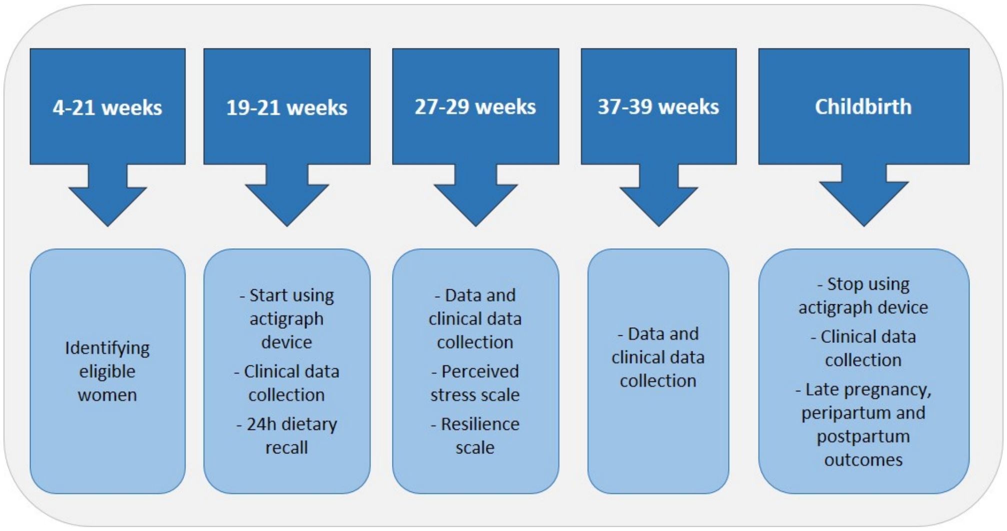

Figure 2

90x47mm (300 x 300 DPI)

STROBE 2007 (v4) checklist of items to be included in reports of observational studies in epidemiology*
Checklist for cohort, case-control, and cross-sectional studies (combined)

Section/Topic	Item #	Recommendation	Reported on page #
**Title and abstract**	1	(*a*) Indicate the study's design with a commonly used term in the title or the abstract	1,2
		(*b*) Provide in the abstract an informative and balanced summary of what was done and what was found	2
**Introduction**			
Background/rationale	2	Explain the scientific background and rationale for the investigation being reported	4-6
Objectives	3	State specific objectives, including any pre-specified hypotheses	6
**Methods**			
Study design	4	Present key elements of study design early in the paper	1,2,7
Setting	5	Describe the setting, locations, and relevant dates, including periods of recruitment, exposure, follow-up, and data collection	7
Participants	6	(*a*) *Case-control study*—Give the eligibility criteria, and the sources and methods of case ascertainment and control selection. Give the rationale for the choice of cases and controls<br>*Cross-sectional study*—Give the eligibility criteria, and the sources and methods of selection of participants	7,8
		(*b*) *Case-control study*—For matched studies, give matching criteria and the number of controls per case	
Variables	7	Clearly define all outcomes, exposures, predictors, potential confounders, and effect modifiers. Give diagnostic criteria, if applicable	11-15
Data sources/ measurement	8*	For each variable of interest, give sources of data and details of methods of assessment (measurement). Describe comparability of assessment methods if there is more than one group	11-15
Bias	9	Describe any efforts to address potential sources of bias	16,17
Study size	10	Explain how the study size was arrived at	15
Quantitative variables	11	Explain how quantitative variables were handled in the analyses. If applicable, describe which groupings were chosen and why	13
Statistical methods	12	(*a*) Describe all statistical methods, including those used to control for confounding	16,17
		(*b*) Describe any methods used to examine subgroups and interactions	16,17
		(*c*) Explain how missing data were addressed	17
		(*d*) *Case-control study*—If applicable, explain how matching of cases and controls was addressed<br>*Cross-sectional study*—If applicable, describe analytical methods taking account of sampling strategy	n/a
		(*e*) Describe any sensitivity analyses	n/a
**Results**			

For peer review only

Participants	13*	(a) Report numbers of individuals at each stage of study—eg numbers potentially eligible, examined for eligibility, confirmed eligible, included in the study, completing follow-up, and analysed	n/a
		(b) Give reasons for non-participation at each stage	n/a
		(c) Consider use of a flow diagram	n/a
Descriptive data	14*	(a) Give characteristics of study participants (eg demographic, clinical, social) and information on exposures and potential confounders	7,8
		(b) Indicate number of participants with missing data for each variable of interest	n/a
Outcome data	15*	*Case-control study*—Report numbers in each exposure category, or summary measures of exposure	n/a
		Cross-sectional study—Report numbers of outcome events or summary measures	n/a
Main results	16	(*a*) Give unadjusted estimates and, if applicable, confounder-adjusted estimates and their precision (eg, 95% confidence interval). Make clear which confounders were adjusted for and why they were included	n/a
		(*b*) Report category boundaries when continuous variables were categorized	n/a
		(*c*) If relevant, consider translating estimates of relative risk into absolute risk for a meaningful time period	
Other analyses	17	Report other analyses done—eg analyses of subgroups and interactions, and sensitivity analyses	17
**Discussion**			
Key results	18	Summarise key results with reference to study objectives	n/a
Limitations	19	Discuss limitations of the study, taking into account sources of potential bias or imprecision. Discuss both direction and magnitude of any potential bias	21
Interpretation	20	Give a cautious overall interpretation of results considering objectives, limitations, multiplicity of analyses, results from similar studies, and other relevant evidence	19-21
Generalisability	21	Discuss the generalisability (external validity) of the study results	21
**Other information**			
Funding	22	Give the source of funding and the role of the funders for the present study and, if applicable, for the original study on which the present article is based	24

*Give information separately for cases and controls in case-control studies and, if applicable, for exposed and unexposed groups in cohort and cross-sectional studies.
Note: An Explanation and Elaboration article discusses each checklist item and gives methodological background and published examples of transparent reporting. The STROBE checklist is best used in conjunction with this article (freely available on the Web sites of PLoS Medicine at http://www.plosmedicine.org/, Annals of Internal Medicine at http://www.annals.org/, and Epidemiology at http://www.epidem.com/). Information on the STROBE Initiative is available at www.strobe-statement.org.

For peer review only
